# Novel 4-alkoxy Meriolin Congeners Potently Induce Apoptosis in Leukemia and Lymphoma Cells

**DOI:** 10.3390/molecules29246050

**Published:** 2024-12-23

**Authors:** Karina S. Krings, Tobias R. Wassenberg, Pablo Cea-Medina, Laura Schmitt, Ilka Lechtenberg, Tanya R. Llewellyn, Nan Qin, Holger Gohlke, Sebastian Wesselborg, Thomas J. J. Müller

**Affiliations:** 1 Institute for Molecular Medicine I, Medical Faculty and University Hospital Düsseldorf, Heinrich Heine University Düsseldorf, Universitätsstraße 1, D-40225 Düsseldorf, Germany; karina.krings@uni-duesseldorf.de (K.S.K.); laura-schmitt9@web.de (L.S.); ilka.hinxlage@uni-duesseldorf.de (I.L.); 2 Institute of Organic Chemistry and Macromolecular Chemistry, Faculty of Mathematics and Natural Sciences, Heinrich Heine University Düsseldorf, Universitätsstraße 1, D-40225 Düsseldorf, Germany; towas100@uni-duesseldorf.de; 3 Institute for Pharmaceutical and Medicinal Chemistry, Heinrich Heine University Düsseldorf, Universitätsstraße 1, D-40225 Düsseldorf, Germany; pablo.cea.medina@hhu.de (P.C.-M.); gohlke@uni-duesseldorf.de (H.G.); 4 Department of Hematology, Oncology and Clinical Immunology, Medical Faculty and University Hospital Düsseldorf, Heinrich Heine University Düsseldorf, Moorenstraße 5, D-40225 Düsseldorf, Germany; tanyarose.llewellyn@med.uni-duesseldorf.de (T.R.L.); nan.qin@med.uni-duesseldorf.de (N.Q.); 5 Center for Integrated Oncology Aachen-Bonn-Cologne-Düsseldorf (CIO ABCD), University Hospital Düsseldorf, Moorenstraße 5, D-40225 Düsseldorf, Germany; 6 Institute of Bio- and Geosciences (IBG-4: Bioinformatics), Forschungszentrum Jülich GmbH, Wilhelm-Johnen-Straße, D-52425 Jülich, Germany

**Keywords:** apoptosis inducers, 7-azaindoles, biheteroaryls, cyclin dependent kinases (CDK), leukemia cell lines, lymphoma cell lines, *meriolins*, one-pot synthesis, palladium catalysis, *Suzuki* coupling

## Abstract

*Meriolins* (3-(pyrimidin-4-yl)-7-azaindoles) are synthetic hybrids of the naturally occurring alkaloids *variolin* and *meridianin* and display a strong cytotoxic potential. We have recently shown that the novel derivative *meriolin* **16** is highly cytotoxic in several lymphoma and leukemia cell lines as well as in primary patient-derived lymphoma and leukemia cells and predominantly targets cyclin-dependent kinases (CDKs). Here, we efficiently synthesized nine novel 2-aminopyridyl *meriolin* congeners (**3a**–**3i**), i.e., *pyrimeriolins*, using a one-pot *Masuda* borylation-*Suzuki* coupling (MBSC) sequence, with eight of them bearing lipophilic alkoxy substituents of varying length, to systematically determine the influence of the alkoxy sidechain length on the biological activity. All the synthesized derivatives displayed a pronounced cytotoxic potential, with six compounds showing IC_50_ values in the nanomolar range. Derivatives **3b**–**3f** strongly induced apoptosis and activated caspases with rapid kinetics within 3–4 h in Jurkat leukemia and Ramos lymphoma cells. The induction of apoptosis by the most potent derivative **3e** was mediated by the intrinsic mitochondrial death pathway, as it was blocked in caspase-9 deficient and Apaf-1 knockdown Jurkat cells. However, as recently shown for *meriolin* **16,** derivative **3e** was able to induce apoptosis in the Jurkat cells overexpressing the antiapoptotic protein Bcl-2. Since tumor cells often inactivate the intrinsic mitochondrial apoptosis pathway (e.g., by overexpression of Bcl-2), these *meriolin* congeners represent promising therapeutic agents for overcoming therapeutic resistance.

## 1. Introduction

**Figure 1:**
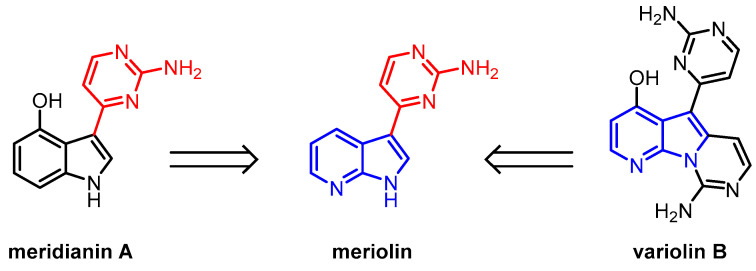
*Meridianin A* and *variolin B* alongside the semi-synthetic hybrid structure of the *meriolins* [10].

Indoles and azaindoles are privileged structural motifs in a variety of natural products and synthetic compounds with pronounced biological activity [1,2,3]. Naturally occurring alkaloids that have often been isolated from marine organisms [4], are for instance *meridianins* and *variolins* (Figure 1). *Variolins* were first isolated from the Antarctic sponge *Kirkpatrickia varialosa* in 1994 and their structure was elucidated [5,6]. *Meridianins* were isolated from the tunicate *Aplidium meridianum* near the South Georgian Islands in 1998 [7,8]. In biological tests, both substance classes have shown a wide variety of biological activity, particularly, cytotoxic, antiplasmodial, and antitumoral properties [4]. The synthetic substance class of *meriolins* can be considered hybrids of *meridianins* and *variolins,* where the latter structural element is simplified to 7-azaindole [9,10]. *Meriolins* were first synthesized alongside *meridianin* derivatives in 2001, although their latent potential as an active substance class was to be discovered later [11,12,13].

**Scheme 1:**
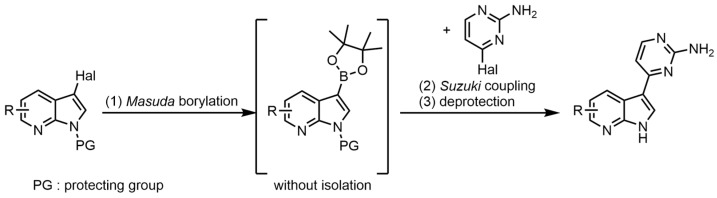
Schematic synthesis of *meriolins* via one-pot MBSC-sequence [10,29,30].

While *meriolins* were found to have analogous properties to those of *meridianins* and *variolins*, it was discovered that they are significantly more active in vitro and in vivo than the latter [14,15]. Additionally, they have been shown to induce apoptosis and inhibit multiple kinases [10,16]. The measured IC_50_ values of effective *meriolins* appear in the low nanomolar range and surpass the activity of *meridianins* and *variolins* [10,17,18]. The change in activity is primarily caused by a change in the binding mode at the hinge region of kinases [16]. Compared to *meridianins* and *variolins*, *meriolins* can form hydrogen bonds in the enzyme pocket via the 7-azaindole moiety with a significantly increased binding affinity [19,20]. Due to their very strong apoptotic and kinase-inhibiting properties, and despite their relatively simple structure compared to other drugs such as *imatinib*, *meriolins* have proven to be a highly interesting substance class for potential therapy [15,21]. Especially regarding their kinase-inhibiting properties, they have shown a broad spectrum of activity and are particularly effective against CDKs 1, 2, 4, 5, and 9 [9,11,15,17,20,22,23]. To a lesser extent, they are also effective against kinases such as glycogen synthase kinase-3 (GSK-3), casein kinase 1 (CK1), dual specificity tyrosine–phosphorylation-regulated kinase 1A (DYRK1A), sphingosine kinase 1 and 2 as well as phosphoinositide-dependent protein kinase 1 (PDK1) [10,14,24]. It has also been shown that they induce cell cycle arrest and activate the intrinsic mitochondrial death pathway [17,21,25]. They exhibit potent antitumor activity in several xenograft cancer models, including Ewing’s sarcoma, LS174T colorectal carcinoma, and U87 glioblastoma [11,15]. Given the frequent dysregulation of the cell cycle and proliferation in cancer, targeting CDKs offers a promising therapeutic approach [26,27]. In this regard, *meriolin* **3**, which inhibits CDK1, CDK2, CDK5, and CDK9, has even advanced into preclinical trials [28]. Due to their broad spectrum of potent biological properties, diversity-oriented and easy synthetic access to a large substance library of *meriolins* is highly desirable, not least to identify an ideal substitution pattern for maximized biological activity [4]. While the first linear syntheses of *meriolins* were multistep with mediocre overall yields and sometimes limited in the mode of functionalization, one-pot *Masuda* borylation-*Suzuki* coupling sequence (MBSC) has emerged as the most efficient synthetic tool for the preparation of *meriolins* from scratch (Scheme 1) [10,29,30].

This one-pot sequence allows concise ligation of substituted heteroaryl fragments starting from two heteroaryl halides [31]. The corresponding heteroaryl halides are readily available or can usually be easily accessed from basic building blocks. Thus, with prior functionalization, novel *meriolin* derivatives are accessible in three steps [10,30,32]. Previous work has shown that the free azaindole hydrogen and the amino group on the heterocycle are vital for any activity of *meriolins* [14,33]. Variations in the azaindole-bound and nitrogen-containing heterocycle appeared to lead to essentially no major changes, although the 2,6-diamino structural motif was found to further reduce the IC_50_ values [10]. Introducing different alkoxy substituents at the 4-position on the azaindole also appears to increase the activity, thus further lowering the IC_50_ values [34]. Despite the known influence of alkoxy substitution on *meriolins*, detailed studies to elucidate the extent of the influence of the sidechain length have not been conducted so far.

Herein, we present a systematic study of the influence of various 4-alkoxy side chains on the biological activity of a series of *meriolin* congeners, which is also accompanied by the simplification of the ligated heterocycle to a 2-aminopyridine moiety. We investigated the apoptotic potential of nine novel *pyrimeriolin* (pyridyl-analogous *meriolins*) derivatives in leukemia and lymphoma cells. The cytotoxicity was evaluated by viability assays, the formation of apoptotic hypodiploid nuclei, and caspase activity assays. In addition, the binding of two novel derivatives to the known target protein CDK9 was modeled. As previously shown for *meriolin* **16**, **31**, and **36** [35], the most potent *pyrimeriolin* derivative **3e** also activated the intrinsic mitochondrial apoptosis pathway in the presence of Bcl-2. Thus, we identified **3e** as a promising therapeutic option for the treatment of leukemia and lymphoma.

## 2. Results and Discussion

### 2.1. Synthesis

A survey on the syntheses of *variolins*, *meridianins*, and their synthetic hybrids shows that the *Masuda* borylation–*Suzuki* coupling (MBSC) not only represents a practical one-pot process for the syntheses of bi(hetero)aryls, but also employs the concept of sequential palladium catalysis, whereby a single catalyst source consecutively catalyzes two reactions [29,36,37]. Even more so, starting from (hetero)aryl halides renders MBSC highly convergent and particularly favorable for synthesizing the substance libraries of bi(hetero)aryls. Besides *merdianins* and *scalaridine A*, we have also successfully applied this one-pot process for the synthesis of *meriolin* libraries [10,21,30,32,35,38] for identifying and studying their mode of action in leukemia and lymphoma cancer cell lines.

Methodological refinement of the *N*-protecting group, the scavenger of excessive pinacolylborane after the borylation step, and deprotection after the *Suzuki* step have finally led to a versatile protocol providing access to the targeted compounds mostly in the good-to-excellent yield after a single chromatographic purification [10]. In particular, it turned out that the yet elegant employment of the Boc-protecting group often could cleave prior to the *Suzuki* step, which was then completely hampered. Tosyl protection, however, is more robust and actually can be cleaved either en route, i.e., as the terminal step of the one-pot sequence, or separately after purification of the *N*-tosylated target molecule [10].

**Scheme 2:**
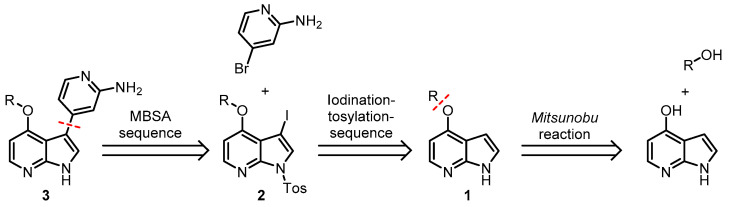
Retrosynthetic breakdown of the alkoxy substituted *pyrimeriolins* **3** [10,14].

Following the general retrosynthetic concept of MBSC of *pyrimeriolins* **3** as targets, the synthesis of 4-substituted *N*-tosyl-3-iodo-7-azaindoles **2** as substrates commences from commercially available 7-azaindoles in multistep synthesis (Scheme 2). Starting with readily available 4-hydroxy-7-azaindole the alkoxy functionality of choice can be introduced via a *Mitsunobu* reaction to give 4-alkoxy 7-azaindoles **1** [14]. Afterward, the iodo functionality and tosyl-protecting group can be introduced in a one-pot fashion following our established protocol [10].

**Scheme 3:**
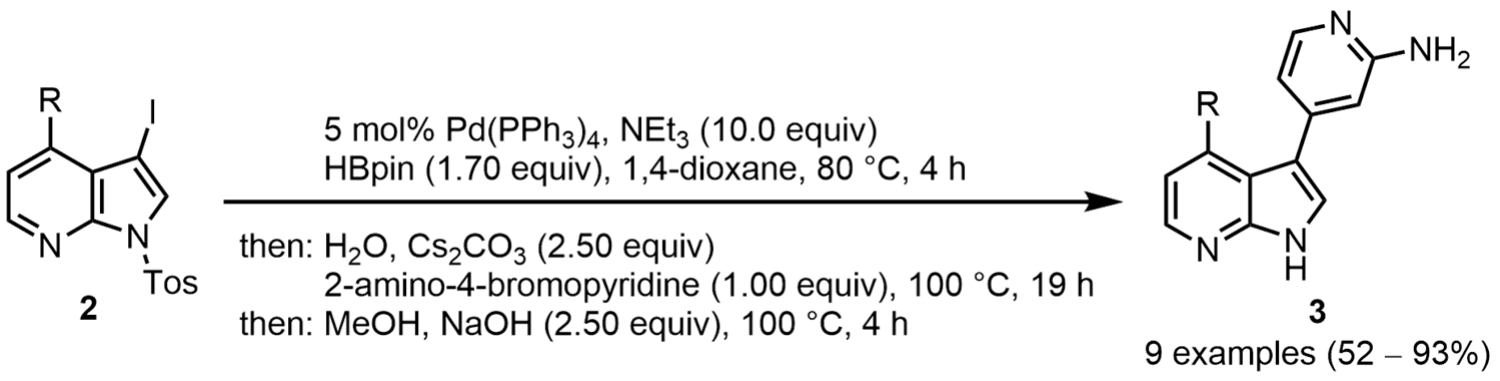
MBSC-sequence conditions for the synthesis of *pyrimeriolins* **3** [10].

**Figure 2:**
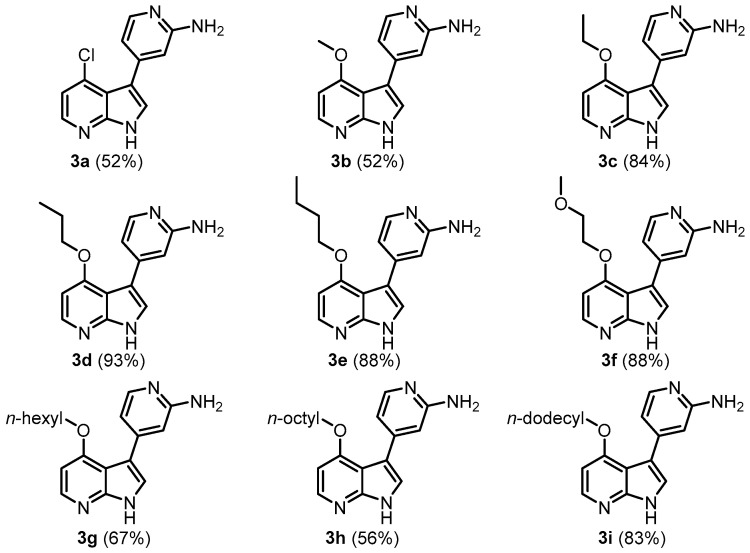
Synthesized *pyrimeriolins* **3** via the MBSC-sequence (yields are given in parenthesis).

Employing the optimized MBSC protocol, nine examples of 4-substituted 3-(2-aminopyrid-4-yl)-7-azaindoles **3**, *pyrimeriolins*, were obtained after chromatographic purification on silica gel in a moderate to excellent yield (Scheme 3). Besides chloro compound **3a,** all the other congeners were 4-alkoxy derivatives with variable alkyl chain length and even a glycol ether moiety (compound **3f**). With this consanguineous series of 4-alkoxy *pyrimeriolins*, structure–activity studies can be conducted to investigate the influence of this distinct structural modification on the *pyrimeriolin* pharmacophore (Figure 2).

### 2.2. Structure

*Pyrimeriolins* possess the characteristic structure of a substituted 7-azaindole moiety connected to an aminopyridine in 3-position. These newly synthesized compounds contain a 2-amine-pyridinyl ligation and are strictly substituted in the 4-position of the 7-azaindole moiety. *Pyrimeriolins* contain six aromatic hydrogen atoms, three each for the azaindole and pyridine substructure, as well as a unique hydrogen atom for the azaindole nitrogen atom and two for the amine group. The aliphatic hydrogen atoms of the alkoxy sidechain can also be easily identified. Therefore, the molecular structure of *pyrimeriolins* **3** was unambiguously assigned by extensive ^1^H and ^13^C NMR spectroscopy and mass spectrometry, and the molecular composition was determined by combustion analysis and/or high-resolution mass spectrometry.

### 2.3. Biological Activity and Influence of Sidechain Length

**Figure 3:**
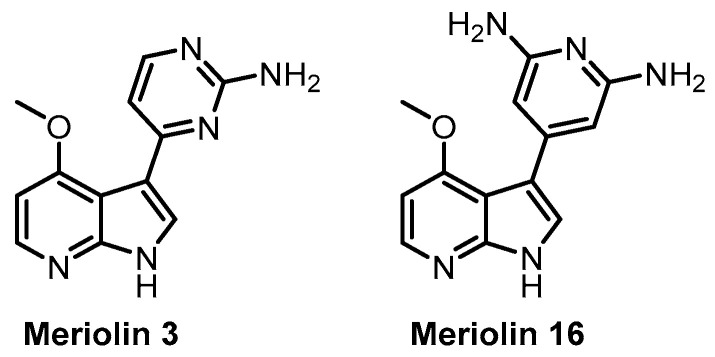
Reference substances for the assays (synthesized according to our previous protocol) [35].

It is well known in the literature that *meriolins* have strong cytotoxic and apoptotic properties [4]. In order to find out more about the structure–activity relationship of the new *pyrimeriolins* **3** and to further investigate the influence of the different alkoxy sidechains, all the compounds were tested for their biological activity in cytotoxicity assays. These tests were carried out against the Ramos cells (Burkitt lymphoma) [39] and the Jurkat J16 cells (acute T-cell leukemia) [40] in a resazurin reduction assay (also known as AlamarBlue^®^ viability assay) [10]. Therefore, the Jurkat T cell leukemia and Ramos Burkitt B cell lymphoma cells were treated for 24 h with the *pyrimeriolins* **3a**–**3i** and compared to the previously described potent derivatives *meriolin* **3** and **16** (Figure 3) [10,15,21].

**Figure 4:**
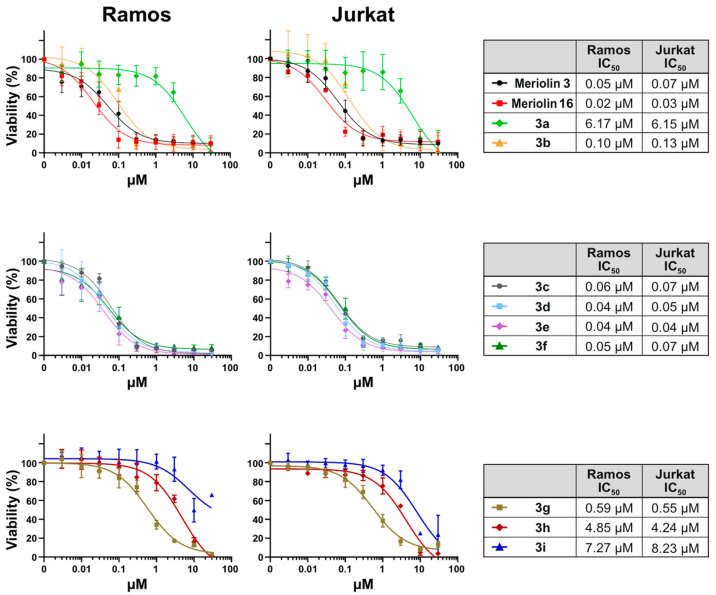
The synthesized *pyrimeriolin* derivatives show prominent cytotoxicity in human Burkitt lymphoma (Ramos) cells and T-cell acute leukemia (Jurkat) cell lines. Ramos and Jurkat cells were incubated for 24 h with increasing concentrations of *meriolin* **3** and **16** and the newly synthesized *pyrimeriolins* **3a**–**3i**. The cell viability was determined with the resazurin reduction assay (AlamarBlue^®^ assay). Data points shown represent the mean (±SD) of three independent experiments. The respective values were normalized to the DMSO control (0.1% *v*/*v*) so that the control was set to 100%. Non-linear regression curve fitting was used to calculate IC_50_ (IC_50_ = half maximum inhibitory concentration) values using Prism 6 (GraphPad software).

Comparing the measured IC_50_ values of the *pyrimeriolins* against the Ramos and Jurkat cell lines, the chlorine substitution, in contrast to the alkoxy substituents, does not seem to be beneficial for the biological activity, as the measured IC_50_ values are very high at around 6.15 µM (Figure 4). This could be due to the varying electronic properties of the aromatic system, as it possesses significantly lower electron density due to the chlorine substituent, which weakens the π-π stacking effect in the enzymes [14]. Decreased electron density in the pyridyl nitrogen atom of the azaindole moiety might also weaken hinge binding in general. This electronic influence on the azaindole could already be observed in the Masuda borylation, where the borylation time of the 4-chloro substituted azaindole was significantly higher compared to the alkoxy substituted compounds (for more details, see Supporting Information).

All alkoxy substituted *pyrimeriolins* **3** exhibited a widely varying range of measured IC_50_ values depending on the length of the alkyl side chain. For compounds **3b**–**3e**, as the alkyl chain length increased, a decrease in the IC_50_ value could be seen from 130 nM for the methoxy compound **3b** to 40 nM for the butoxy-substituted compound **3e**. The introduction of additional heteroatoms seemed to lead to a slight reduction in activity (**3f**). This effect can be attributed to the varying electronic properties of the sidechain. As the chain length increased beyond the butoxy substituent, a large increase in the IC_50_ values could be observed (**3g**–**3i**). The IC_50_ value of compound **3g** was an order of magnitude higher compared to compound **3e**. A further order of magnitude increase could be observed for the change to the octoxy sidechain in compound **3h**. In addition, further extension of the side chain to dodecoxy apparently did not further lower the IC_50_ values. Comparing *pyrimeriolin* **3b** with the thoroughly studied *meriolin* **3**, which was measured as a reference, the data indicate that altering the structure from aminopyrimidinyl to aminopyridyl was accompanied by a decrease in the biological activity.

In general, for short alkoxy chain lengths, increasing the chain length leads to an increase in biological activity (**3b**–**3e**). This effect reaches its optimum for the butoxy sidechain and further extension of the side chain length leads to an even stronger decrease in biological activity (**3g**–**3i**). Echalier et al. already investigated the effects of certain alkoxy side chain lengths and attributed the rising biological activity to a better interaction with the glycine-rich loop and ribose binding region in the CDKs [14,34]. Incidentally, this effect could also be attributed to a change in cell permeability along with the change in solubility due to increasing lipophilicity.

**Figure 5:**
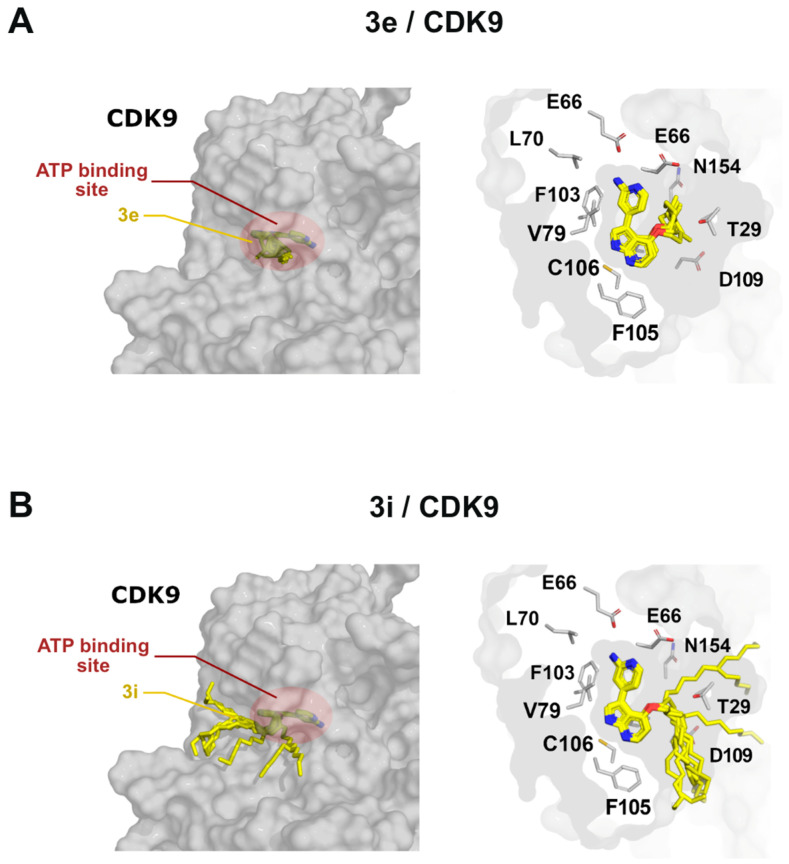
Binding of *pyrimeriolin* **3e** and **3i** to CDK9. Left panels show a general view of the 10 best binding poses of compounds **3e** (**A**) and **3i** (**B**) placed in the active site of CDK9. The right panels show slices through the binding site. The sidechains of the binding site amino acids are depicted as white sticks, whereas the best 10 scored binding poses for *pyrimeriolins* **3e** and **3i** are shown as yellow sticks.

Molecular modeling of compound **3e,** bound to the ATP binding site of CDK9, confirmed that the alkyl group did not seem to interfere with binding to CDK9but integrated completely into the pocket (Figure 5A). However, longer alkyl groups, as in the case of compound **3i**, protruded from the ATP binding site of CDK9 but did not compromise the binding mode (Figure 5B). Integration of the sidechain into the pocket was unexpected, since the molecular modeling of *meriolin* **16** in our previous studies indicated a protrusion of the smaller methoxy sidechain out of the pocket, hinting at a difference in sidechain orientation for varying lengths [21].

**Figure 6:**
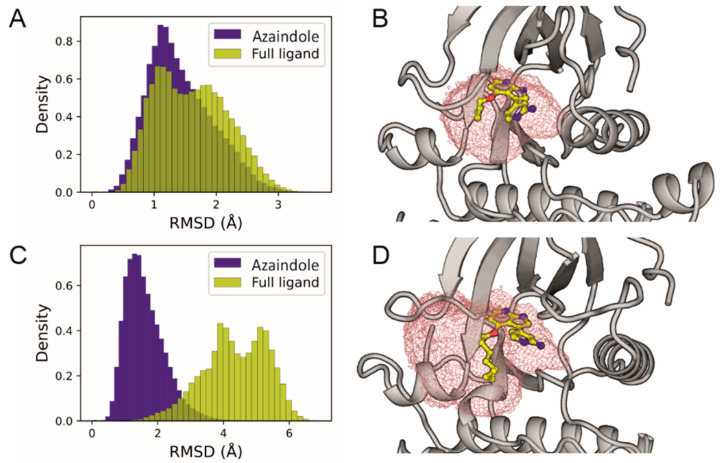
Molecular dynamics simulations starting from the binding poses of *meriolin* **3e** and **3i** in CDK9. (**A**) Histogram of the non-fitted RMSD obtained for the full *pyrimeriolin* **3e** ligand (yellow) or only its 7-azaindolyl moiety (blue). (**B**) Active site of CDK9 (shown as gray cartoon) showing the effective volume occupied by *pyrimeriolin* **3e** (red grid), the binding pose of the ligand in the last simulation frame is shown as yellow sticks. (**C**) Same as in (**A**) but for **3i**. (**D**) Same as in (**B**) but for **3i**.

The robustness of the obtained docking poses was further assessed by performing molecular dynamic simulations of both protein-ligand complexes. Ten replicas of 500 ns each were performed for each system. To evaluate whether the ligands remained stably bound in the active site throughout the sampled simulation time, we measured the non-fitted RMSD of the 7-azaindolyl moiety or the full ligand and also calculated the effective volume occupied by the ligand during the simulations (Figure 6). The results show that *pyrimeriolin* **3e** remained structurally invariant throughout the entire simulation, with low RMSD values observed for the 7-azaindolyl core and slightly higher values when the alkyl tail is considered (Figure 6A); this is also reflected in a small effective occupied volume (Figure 6B). For *pyrimeriolin* **3i**, we also observed a robust binding of the 7-azaindolyl moiety, with similarly small RMSD values (Figure 6C). As expected, when the larger alkyl tail of **3i** was considered, the RMSD reached higher values, indicating that this segment was highly flexible during the simulations. This was confirmed by the volumetric assessment, as there was a large effective occupied volume at the edge of the active site where the alkyl tail was located (Figure 6).

**Figure 7:**
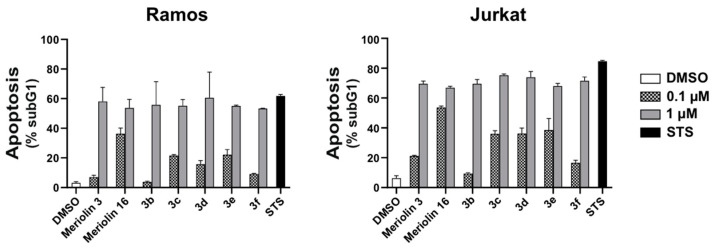
Apoptosis-induced DNA fragmentation by the (*pyri*)*meriolin* derivatives. Ramos or Jurkat cells were treated with 0.1 µM or 1.0 µM of the respective *(pyri)meriolin* derivatives, 0.1% *v*/*v* DMSO (diluent control), or 2.5 µM staurosporine (STS; as a positive control for apoptosis induction) for 24 h. Apoptosis-induced DNA degradation was measured via flow-cytometric measurement of propidium iodide stained apoptotic hypodiploid nuclei [41]. The experiment was performed in triplicates (error bars = mean ± SD).

**Figure 8:**
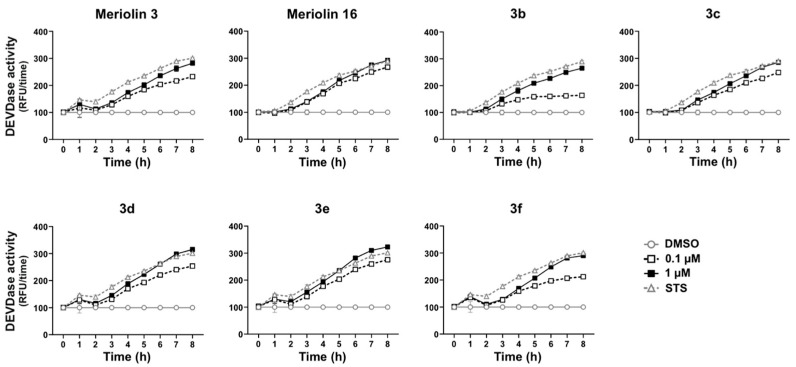
Compounds **3b**–**3f** induce caspase activation in rapid kinetics in Ramos cells. Ramos cells were treated with 0.1 µM or 1.0 µM of the respective *(pyri)meriolin* derivatives for up to 8 h. As a positive control, the potent apoptotic stimulus staurosporine (2.5 µM; STS) was included in each kinetic. Subsequently, caspase-3 activity was determined by measurement of the fluorescence of the profluorescent caspase-3 substrate DEVD-AMC in a spectrofluorometer. Error bars represent the mean ± SD of one experiment performed in triplicates.

**Figure 9:**
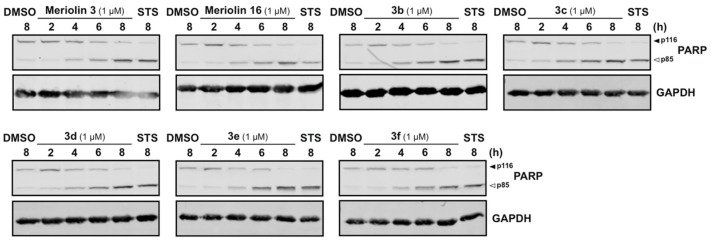
Compounds **3b**–**3f** induce the cleavage of the caspase substrate PARP in rapid kinetics in Ramos cells. 1 × 10^6^ Ramos cells were treated with 1 µM of the respective *(pyri)meriolin* derivatives for 2, 4, 6 and 8 h. In addition, the cells were treated for 8 h with DMSO (0.1% *v*/*v*) as a diluent control or for 8 h with 2.5 µM staurosporine. The resulting cells were prepared for immunoblot analysis and antibodies of caspase-3 substrate PARP were used to visualize the cleavage of this substrate. The cleavage of the caspase substrate PARP was detected by immunoblotting. Solid arrowheads indicate the uncleaved form of PARP (p116); open arrowheads indicate the cleaved form (p85). Immunoblotting for glyceraldehyde 3-phosphate dehydrogenase (GAPDH) was used as loading control.

Next, we focused on the most potent compounds **3b**–**3f** concerning their apoptotic potential. Therefore, we monitored the apoptosis-related DNA degradation by flow-cytometric measurement of propidium iodide stained apoptotic hypodiploid nuclei [41] in Ramos and Jurkat cells after 24 h. As shown in Figure 7, all the tested compounds induced apoptosis to a similar extent as *meriolin* **3** and **16** and were almost as potent as the broad kinase inhibitor and potent apoptotic stimulus staurosporine (STS), which was used as positive control. Subsequently, we analyzed the catalytic caspase activity using the profluorescent caspase-3 substrate Ac-DEVD-AMC. An 8 h kinetic analysis revealed rapid activation of caspase-3 as early as 3–4 h in the Ramos cells upon treatment with compound **3b**–**3f**. However, the *meriolin*-induced caspase-3 activation was not as fast as upon treatment with staurosporine (Figure 8). Likewise, significant cleavage of the caspase substrate poly(ADP-ribose) polymerase 1 (PARP) was observed in the Ramos cells, with kinetics similar to those detected in the caspase activity assay (Figure 9).

Next, we investigated which apoptosis signaling pathway was affected by the most cytotoxic derivative **3e**. There existed two major apoptosis signaling pathways—the extrinsic death receptor pathway and the intrinsic mitochondrial apoptosis pathway. Death receptor-mediated apoptosis was initiated by the stimulation of receptors (such as CD95/Apo-1/Fas, TRAIL-R1, or TRAIL-R2) with their respective ligands (e.g., CD95L/Apo-1L/FasL or TRAIL). This led to the activation of the initiator caspase-8, which subsequently activated the effector caspase-3. The mitochondrial death pathway was triggered by cellular stress, such as DNA damage caused by radiotherapy and chemotherapy. This pathway was initiated by the mitochondrial release of cytochrome c, a process mediated by proapoptotic Bcl-2 family proteins, including Bax and Bak. Antiapoptotic Bcl-2 proteins, such as Bcl-2, Bcl-xL, and Mcl-1, inhibited this release by neutralizing the proapoptotic Bcl-2 members, thereby blocking the mitochondrial death pathway [42]. Once in the cytosol, cytochrome c bound to the adapter protein Apaf-1, leading to the formation of the so-called apoptosome, a high molecular weight complex that activates initiator caspase-9. Activated caspase-9 then cleaved and activated effector caspases-3 and -7, which executed apoptosis [43,44].

**Figure 10:**
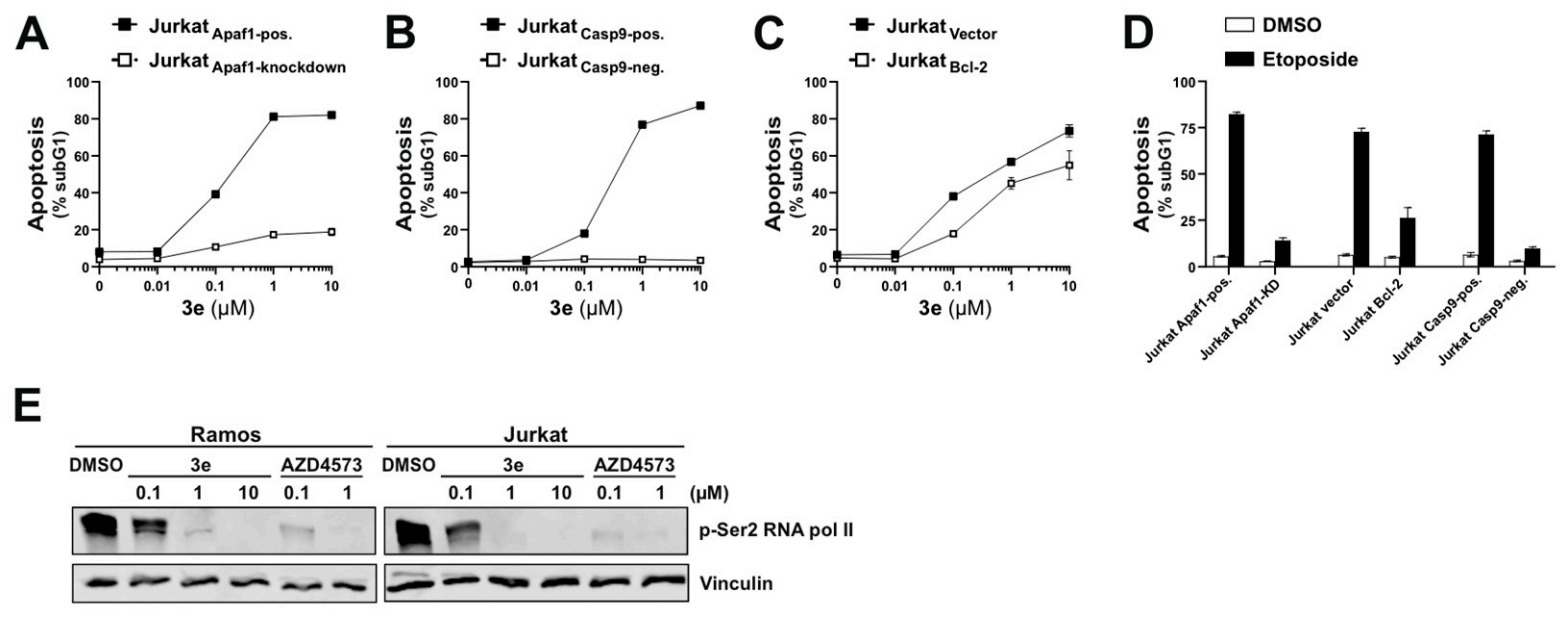
*Meriolin* derivate **3e** activates the mitochondrial apoptosis pathway in Bcl-2 overexpressing Jurkat cells. (**A**) **3e**-induced apoptosis requires Apaf-1. The CRISPR inhibition system was used to achieve a stable knockdown of Apaf-1 [35]. Apaf-1 proficient (Jurkat-Apaf1-pos.; black squares) and Apaf-1 knockdown Jurkat cells (Jurkat-Apaf1-knockdown; white squares) were treated with increasing concentrations of compound **3e**. After 24 h, apoptosis was assessed by the flow cytometric measurement of apoptotic hypodiploid nuclei. (**B**) **3e**-induced apoptosis requires caspase-9. Caspase-9-proficient Jurkat cells (Jurkat Casp9-pos., black squares) or caspase-9-deficient Jurkat cells (Jurkat Casp9-neg., white squares) [45] were treated with increasing concentrations of compound **3e**. After 24 h, apoptosis was assessed by the flow cytometric measurement of apoptotic hypodiploid nuclei. (**C**) **3e** induces apoptosis in the presence of antiapoptotic Bcl-2. Jurkat cells stably transfected with vectors encoding Bcl-2 (Jurkat Bcl-2; white squares) [46] or empty vector (Jurkat vector; black squares) were treated with increasing concentrations of compound **3e**. After 24 h, apoptosis was assessed by the flow cytometric measurement of apoptotic hypodiploid nuclei. (**D**) The induction of apoptosis by the DNA-damaging anticancer drug etoposide (used as a control for the activation of the mitochondrial apoptosis pathway) was blocked in caspase-9 deficient and Apaf-1 knockdown Jurkat cells, as well as in Bcl-2 overexpressing Jurkat. Apaf-1 proficient (Jurkat-Apaf1-pos.) or Apaf-1 knockdown Jurkat cells (Jurkat-Apaf1-knockdown), caspase-9 proficient Jurkat cells (Jurkat Casp9-pos.) or caspase-9 deficient (Jurkat Casp9-neg.) Jurkat cells or Jurkat cells overexpressing Bcl-2 (Jurkat Bcl-2) or empty vector (Jurkat vector) were treated with 50 µM etoposide or 0.1% *v*/*v* DMSO (diluent control). After 24 h, apoptosis was assessed by flow cytometric measurement of the apoptotic hypodiploid nuclei. (**E**) Ramos and Jurkat cells were incubated with *pyrimeriolin* derivative **3e** (0.1, 1.0 or 10 µM) or DMSO (0.1% *v*/*v*) as a diluent control for 6 h. Treatment with 0.1 or 1.0 µM of the CDK9-specific inhibitor AZD4573 [47] served as positive control. Inhibition of CDK9-mediated phosphorylation of RNA polymerase II at Ser2 (p-Ser2 of RNA pol II) was monitored by immunoblotting. Vinculin served as a loading control.

Since activation of the mitochondrial apoptosis pathway depends on the Apaf-1-mediated activation of caspase-9 within the apoptosome, we investigated whether compound **3e** could induce apoptosis in the Jurkat cells with stable transcriptional silencing of Apaf-1 achieved by CRISPR interference [35]. As shown in Figure 10A, **3e-**induced apoptosis was blocked in the Apaf-1 knockdown cells. This finding that **3e** apparently required apoptosome formation was further supported by the observation that **3e**-induced apoptosis was completely abolished in the caspase-9-deficient Jurkat cells (Figure 10B).

Tumor cells frequently acquire therapy resistance through the overexpression of anti-apoptotic Bcl-2 proteins (such as Bcl-2, Bcl-xL, or Mcl-1). Interestingly, *pyrimeriolin* **3e** mediated apoptosis was only slightly attenuated but not blocked in the Jurkat cells overexpressing Bcl-2 (Figure 10C) compared to the respective vector control cells. As expected, the induction of apoptosis by the DNA-damaging anticancer drug etoposide was prevented in the Jurkat cells overexpressing Bcl-2 or in Jurkat cells with Apaf-1 knockdown or deficient for caspase-9 (Figure 10D). The observation that *pyrimeriolin* **3e** activates the mitochondrial apoptosis pathway even in the presence of antiapoptotic Bcl-2, is consistent with our previous results for *meriolins* **16**, **31**, and **36** [21].

We have previously shown in a kinome screen that *meriolin* **16** and **36** inhibit (with the exception of CDK4 and 6) almost all cyclin-dependent kinases, including CDK9 [21]. Inhibition of transcriptional CDKs (such as CDK9) has been demonstrated to trigger apoptosis via the inhibition of RNA polymerase II and subsequent transcriptional downregulation of the short-lived antiapoptotic Bcl-2 protein Mcl-1 [47]. Therefore, we investigated the effect of **3e** on the CDK9-mediated phosphorylation of RNA polymerase II at Ser2, which is crucial for transcription initiation. As shown in Figure 10E, compound **3e** inhibited the phosphorylation of RNA polymerase II at Ser2 in Jurkat and Ramos cells to a similar extent as the CDK9-specific inhibitor AZD4573, which served as a positive control.

Taken together, we could show that the most potent derivatives, **3b**–**3f**, induced apoptosis, activated caspases, and led to the cleavage of the caspase substrate PARP, with rapid kinetics (within 3–4 h) in the Ramos cells (Figure 7, Figure 8 and Figure 9). The most potent *pyrimeriolin* derivative, **3e**, was found to be more active than *meriolin* **3** and was therefore subjected to further investigation concerning its apoptosis signaling. As previously shown for *meriolin* **16** [21], compound **3e** activated the intrinsic mitochondrial apoptosis pathway, as apoptosis was abrogated in Jurkat cells deficient for caspase-9 and Apaf-1 knockdown Jurkat cells (Figure 10A–D). As already shown for *meriolin* **16** and **36** [35], compound **3e** also prevented the CDK9-mediated phosphorylation of RNA polymerase II at Ser2 which was crucial for transcription initiation. Intriguingly, though compound **3e** induced apoptosis via the intrinsic mitochondrial death pathway, overexpression of the antiapoptotic protein Bcl-2 in the Jurkat cells did not inhibit apoptosis induction. This corroborates our earlier findings with *meriolins* **16**, **31**, and **36**, which were also able to induce apoptosis in Bcl-2 overexpressing Jurkat cells [35]. However, when we used Jurkat cells overexpressing antiapoptotic Bcl-xL or the Bax- and Bak-deficient human B cell Burkitt lymphoma cell line DG75, apoptosis induction by *meriolin* 16, 31, and 36 was blocked [35]. Usually, antiapoptotic Bcl-2 proteins (such as Bcl-2, Bcl-xL or Mcl-1) counteract proapoptotic Bcl-2 members (such as Bax, Bak), and the ratio between pro- and antiapoptotic Bcl-2 proteins determines the outcome of cell death or survival.

The apparent conundrum of why *meriolins* can induce apoptosis in Jurkat cells overexpressing Bcl-2, but not in Bcl-xL overexpressing cells can be resolved by considering the mechanism of apoptosis induction by CDK inhibitors. In this context, Cidado et al. could show that the CDK9-specific inhibitor AZD4573 inhibits the CDK9-mediated activation of RNA polymerase II, which in turn leads to a reduction in transcriptional activity and thereby to the downregulation of short-lived proteins, such as Mcl-1. In addition, they could show that Bak appears to be primarily responsible for apoptosis-induction via CDK9-inhibition [47]. In contrast to Bcl-2, which can only bind to Bax—Mcl-1 and Bcl-xL, it can interact with both Bax and Bak [48,49,50]. Since CDK9-inhibition obviously induces apoptosis via Bak (due to the downregulation of short-lived Mcl-1) this explains why the overexpression of Bcl-2 does not inhibit *meriolin*-induced apoptosis (in contrast to overexpression of Bcl-xL or Bak-knockdown).

As tumor cells frequently overexpress the antiapoptotic protein Bcl-2 in order to acquire therapy resistance [42], this renders *pyrimeriolins* as versatile candidates for anticancer drugs to overcome therapy resistance, particularly due to their ability to inhibit the CDKs involved in both tumor cell proliferation and cell survival.

## 3. Materials and Methods

### 3.1. Syntheses

#### 3.1.1. General Procedure *Mitsunobu* Reaction (Compound **1e**) [14]

4-hydroxy-7-azaindole (671 mg, 5.00 mmol) and *n*-butanol (445 mg, 6.00 mmol) were placed in a dry Schlenk tube with magnetic stir bar under nitrogen and dissolved in dry THF (50 mL). In parallel, triphenylphosphane (3.15 g, 12.0 mmol) was placed in a 50 mL round-bottom flask with a septum. After evacuation and filling the vessel with nitrogen three times, a solution of diethyl azodicarboxylate (1.74 g, 10.0 mmol) in toluene was added, followed by dry THF (30 mL). This solution was then added to the dissolved 4-hydroxy-7-azaindole solution and stirred at room temp for 2.5 h. After the reaction was completed, the solvents were removed in vacuo and the residue was adsorbed on Celite^®^. After column chromatography on silica gel (98:2 dichloromethane/methanol) the isolated product was further purified by recrystallization in dichloromethane, furnishing 4-butoxy-1*H*-pyrrolo[2,3-b]pyridine (**1e**) (342 mg, 36%) as colorless crystals (for full experimental and analytical details, see the Supporting Information).

#### 3.1.2. General Procedure Iodination–Tosylation Sequence (Compound **2e**) [10]

4-butoxy-1*H*-pyrrolo[2,3-b]pyridine (**1e**) (951 mg, 5.00 mmol) and finely powdered potassium hydroxide (701 mg, 12.5 mmol) were dissolved in *N*,*N*-dimethylformamide (30 mL) in a 250 mL round-bottom flask with a magnetic stir bar and a dropping funnel and stirred for 10 min. Afterwards, a solution of iodine (1.28 g, 5.05 mmol) in *N*,*N*-dimethylformamide (30 mL) was added to the dropping funnel and added to the reaction mixture at room temp over 10 min. After the addition was completed, the reaction mixture was stirred at room temp for 45 min. A solution of *p*-toluenesulfonyl chloride (2.00 g, 10.5 mmol) in *N*,*N*-dimethylformamide (30 mL) was then added dropwise at 0 °C over 20 min. The reaction mixture was then stirred at room temp for 3 h. After the completed reaction a cooled aqueous sodium thiosulfate solution (100 mL) was added dropwise to the solution and the mixture was stirred at 0 °C for another 30 min. The dropping funnel was exchanged for a stopper and the solution was stored in a refrigerator for 18 h. The precipitate was filtered and washed with ice water. The crude product was then dissolved in dichloromethane, adsorbed on Celite^®^ and purified by column chromatography on silica gel (9:1 *n*-hexane/acetone). After drying in vacuo at 80 °C for 8 h compound **2e** (1.98 g, 84%) was isolated as a beige solid (for full experimental and analytical details, see the Supporting Information).

#### 3.1.3. General Procedure MBSC (Compound **3e**) [10]

4-butoxy-3-iodo-1-tosyl-1*H*-pyrrolo[2,3-b]pyridine (**2e**) (470 mg, 1.00 mmol) was added to a dry Schlenk tube with magnetic stir bar together with tetrakis(triphenylphosphane)palladium(0) (57.8 mg, 0.05 mmol) in dry 1,4-dioxane (5 mL). After degassing the solution with nitrogen for 10 min, triethylamine (1.40 mL, 100 mmol) and 4,4,5,5-tetramethyl-1,3,2-dioxaborolane (0.25 mL, 1.70 mmol) were added and the solution was stirred at 80 °C for 4 h. The reaction mixture was cooled to room temp and deionized water (5 mL) was carefully added. Then, cesium carbonate (815 mg, 2.50 mmol) and 4-amino-2-bromopyridine (173 mg, 1.00 mmol) were added to the mixture and the mixture was stirred at 100 °C for 19 h. After cooling to room temp methanol (5 mL) and finely powdered sodium hydroxide (100 mg, 2.50 mmol) were added to the reaction mixture before stirring at 100 °C (preheated oil bath) for 4 h. After cooling to room temp, the solvent was removed in vacuo and the residue was adsorbed on Celite^®^. The product was purified by column chromatography on silica gel (98:2:1 dichloromethane/methanol/aqueous ammonia). The product was triturated with *n*-hexane in the ultrasound bath for 30 min. The supernatant was removed and the purified product dried in vacuo at 80 °C for 8 h to give *pyrimeriolin* **3e** (0.88 mmol, 88%) as a beige solid.

The details are as follows: ^1^H NMR (DMSO-d_6_, 300 MHz): *δ* 0.89 (t, ^3^*J_HH_* = 7.4 Hz, 3H), 1.29–1.45 (m, 2H), 1.69–1.82 (m, 2H), 4.12 (t, ^3^*J_HH_* = 6.3 Hz, 2H), 5.67 (s, 2H), 6.44–6.86 (m, 3H), 7.52 (s, 1H), 7.82 (d, ^3^*J_HH_* = 5.4 Hz, 1H), 8.11 (d, ^3^*J_HH_* = 5.5 Hz, 1H), 11.87 (s, 1H). ^13^C NMR (DMSO-d_6_, 75 MHz): *δ* 13.65 (CH_3_), 18.78 (CH_2_), 30.42 (CH_2_), 67.58 (CH_2_) 98.91 (CH), 106.79 (C_quat_), 107.26 (CH), 112.90 (CH), 114.01 (C_quat_), 123.09 (CH), 143.66 (C_quat_), 145.09 (CH), 146.61 (CH), 150.89 (C_quat_), 159.30 (C_quat_), 159.57 (C_quat_). MS (EI, *m*/*z* (%)): 283 (18), 282 ([M]^+^, 78), 227 ([C_12_H_11_N_4_O]^+^, 17), 226 ([C_12_H_10_N_4_O]^+^, 100), 198 ([C_11_H_10_N_4_]^+^, 11); the ESI HRMS calcd. for [C_16_H_19_N_4_O]^+^: 283.1559; found: 283.1534. HPLC *t_r_*: 1.5 min (>99% purity). (For full experimental and analytical details, see the Supporting Information).

### 3.2. Reagents

Staurosporine (STS) (#9300) was obtained from LC Laboratories (Woburn, MA, USA). *Meriolin*-3 (445821) was purchased from Merck Millipore (Darmstadt, Germany). All the other substances for which a manufacturer is not explicitly specified were obtained from Carl Roth.

### 3.3. Cell Lines and Cell Culture

The Jurkat cells (human T cell leukemia; #ACC-282) were obtained from DSMZ. Ramos cells (human Burkitt B cell lymphoma) were kindly provided by Michael Engelke (Institute of Cellular and Molecular Immunology, University Hospital Göttingen, Göttingen, Germany). The cells were maintained in RPMI 1640 media with 10% FCS, 100 U/mL penicillin and 100 µg/mL streptomycin at 5% CO_2_ at 37 °C and stable humidity. Jurkat cells with caspase-9-deficiency were generously supplied by Klaus Schulze-Osthoff (Interfaculty Institute for Biochemistry, University of Tübingen, Germany) [45] and retrovirally transduced with either empty pMSCVpuro (Clontech, Heidelberg, Germany) or pMSCVpuro containing cDNAs encoding for untagged human wild-type caspase-9, which was previously described [51]. Bcl-2 overexpressing Jurkat cells and corresponding vector control cells were kindly provided by Claus Belka (Ludwig-Maximilians University, Munich, Germany) and have been previously described [46]. Jurkat cells with Apaf-1 knockdown were generated using CRISPR/Cas as previously described [21].

### 3.4. Cytotoxicity Measurements

For cytotoxicity determination of the (*pyri*)*meriolin* derivates, resazurin reduction assays (also known as AlamarBlue^®^ assays) were performed. Suspension cells were seeded at a density of 5 × 10^5^ cells per well in a 96-well plate and immediately treated with increasing concentration of the respective substance. After 22 h of incubation, resazurin (Sigma-Aldrich, St.Louis, MO, USA, #R7017) was added to a final concentration of 40 µM and incubated for an additional two hours. Therefore, after 24 h, the measurement of the fluorescence of resorufin (excitation (Ex): 560 nm, emission (EM); 590 nm) could be measured in a microplate spectrophotometer (BioTek, Winooski, VT, USA, Synergy Mix plate reader). When added to living cells, resazurin was modified by the reduction environment of the living cells and reduced to resorufin, which is a measure of aerobic respiration and thus for cell viability. The viability of the treated cells was normalized by the cells treated with the DMSO (0.1% *v*/*v*) control, and thus the DMSO control was set to 100% viability. Data points were then visualized using dose–response curves from PRISM v7.01 (GraphPad Software, La Jolla, CA, USA).

### 3.5. FACS-Based Analysis of Apoptotic Cell Death

The method described in Nicoletti et al. was used to measure apoptosis-induced fragmented DNA [41]. For this purpose, the nuclei were isolated by lysing the cells in a hypotonic lysis buffer (1% sodium citrate, 0.1% Triton X-100, 50 µg/mL propidium iodide) and staining it with propidium iodide, followed by flow cytometric analysis. By this staining method, it can be distinguished between the cells with a DNA content of 2N and more or less so that all the cell nuclei with less than 2N of DNA content were identified as apoptotic. All flow cytometric analyses were performed on an LSR-Fortessa™ (Becton Dickinson, Heidelberg, Germany).

### 3.6. Fluorometric Caspase-3 Activity Assay

For the caspase-3 activity assay, Ramos cells were seeded at a density of 5 × 10^5^ cells per well in a 96-well plate and treated with the *meriolin* derivatives or the respective control for the specified time (kinetics 0–8 h). After treatment, the cells were harvested by centrifugation at 600× *g*, the supernatant was removed, and the cells were lysed on ice for 10 min with 50 µL of cold lysis buffer (20 mM HEPES, 84 mM KCl, 10 mM MgCl_2_, 200 µM EDTA, 200 µM EGTA, 0.5% NP40, 1 µg/mL leupeptin, 1 µg/mL pepstatin, 5 µg/mL aprotinin). Lysates were mixed with 150 µL of ice-cold reaction buffer (50 mM HEPES, 100 mM NaCl), 10% sucrose, 0.1% CHAPS, 2 mM CaCl2, 13.35 mM DTT) containing 70 µM of the profluorescent caspase substrate Ac-DEVD-AMC (Biomol GmbH, Hamburg, Germany, #ABD-13420). A Synergy Mix microplate reader was used to measure the kinetics of AMC release by measuring the AMC fluorescence intensity (excitation (Ex): 360 nm; emission (EM): 450 nm) every 2 min over 120 min. The slope of the linear range of the fluorescence increase (Δrfu/min) served as a measure of the DEVDase activity and was subsequently normalized to the DMSO control. Measurement of the fluorescence of the profluorescent caspase-3 substrate DEVD-AMC, and thus DEVDase activity, was considered a marker of caspase-3 activity.

### 3.7. Protein Immunoblotting

Protein immunoblotting was performed to analyze the protein content and cleavage of the caspase substrate PARP. For this purpose, Ramos cells were seeded at a density of 2 × 10^6^ cells/mL, treated as indicated, and harvested by centrifugation (3000× *g*, 5 min) followed by freezing. The cell pellets were thawed on ice and then the pellets were lysed with lysis buffer (20 mM Tris-HCl, 150 mM NaCl, 1% *v*/*v* Triton X-100, 0.5 mM EDTA, 0.5% sodium deoxycholate, protease inhibitors (Sigma-Aldrich, St.Louis, MO, USA, #P2714) and PhosStop (Roche, Basel, Switzerland, 04906837001)) for 30 min on ice with vortexing. In the case of immunoblots for p-Ser2-RNA polymerase II (Figure 10E), benzonase (10 U/mL, Merck, Darmstadt, Germany, #70664-3) was added to the lysis buffer to dissolve the DNA for examination of DNA-bound proteins. Subsequently, the dissolved proteins in the cell lysate were purified by centrifugation (13,300× *g*, 15 min) and subsequent removal of the pellet from cell debris. The Bradford assay was used to determine the protein concentration and samples were then uniformly diluted with lysis buffer and Lämmli buffer. Then, SDS-Page and immunoblots were performed, and specific antibodies were used for the detection of respective proteins (anti-PARP: 2000 (Enzo, New York, NY, USA, #BML-SA250); p-Ser2-RNA polymerase II 1:1000 (Abcam, Cambridge, UK, #ab5095); anti-GAPDH 1:5000 (Abcam, Cambridge, UK, #ab8245); anti-vinculin 1:2000 (Sigma-Aldrich, St.Louis, MO, USA, #V4139)). In addition, detection of the target proteins on the PVDF membrane using the LI-COR Odyssey^®^ Imaging System was performed by using fluorescence-coupled secondary antibodies (LI-COR Biosciences, Lincoln, NE, USA).

### 3.8. Molecular Modeling of CDK-Pyrimeriolin Complexes

The crystal structure of CDK9 (PDB 3TNH) was prepared for docking using the protein preparation wizard implemented in Schrödinger’s Maestro suite, setting the pH for titratable residues at 7.0. The structures of the *meriolin* derivatives were prepared using LigPrep, predicting the most likely protonation state with Epik [52]. Molecular docking was performed using Glide in extra precision mode [53]. A grid box of 35 Å was placed in the active site by first aligning the structure of CDK9 to the homologous CDK2 (PDB 3BHT) and then using the crystallized *meriolin* derivative positioned in the active site of CDK2 as a reference for the center of the box. Fifty poses per ligand were generated using the OPLS_2005 force-field [54]. The 10 poses with the highest docking scores were analyzed using PyMOL [55].

For molecular dynamics simulations, the charges for *pyrimeriolin* derivatives **3e** and **3i** were parametrized by performing a quantum mechanical optimization at the Hartree-Fock 6-31G* level of theory with Gaussian16 [56], and then fitting point charges using the RESP method implemented in antechamber [57]. All the simulations were performed using Amber24, with the ff19SB [58] force field for proteins and the general amber force field 2 for ligands (GAFF2). The systems were solvated with the TIP3P water model in a truncated octahedron extending 14 Å beyond any solute atom. All the simulations were performed using the GPU implementation of the Particle Mesh Ewald Method [59], with a cut-off of 10 Å. All the bonds involving hydrogen atoms were constrained using the SHAKE algorithm. All the minimization, thermalization, and pressurization steps were performed as previously described elsewhere [21].

The RMSD of the ligand was calculated using the *rms* command in cpptraj [60]. First, the trajectory was fitted to the first frame using only the backbone atoms of the protein. Then, the non-fitted RMSD of the *pyrimeriolin* ligand was calculated specifying either all heavy atoms or all heavy atoms belonging to the 7-azaindolyl moiety. The spatial density occupied by the ligand was also calculated in cpptraj, by first defining the boundaries of a grid using the *bounds* command with a spacing of 0.5 Å and setting the protein atoms in the mask, then a density map was generated using the command *crdaction* over the defined grid, setting the ligand as mask.

## 4. Conclusions

The MBSC is an invaluable tool for the construction of bis(hetero)aryl systems in the synthesis of natural compounds or their analogs. We concisely synthesized nine novel *pyrimeriolins,* mostly containing alkoxy side chains of varying length, and investigated their effect on the biological activity. We could show that *pyrimeriolins* **3b**–**3f** are highly cytotoxic in Jurkat leukemia and Ramos lymphoma cells with IC_50_ values in the nanomolar range and that they induce apoptosis in rapid kinetics. In particular, compound **3e** with a butoxy substituent showed lower IC_50_ values than the established *meriolin* **3** and triggered the mitochondrial death pathway even in the presence of the antiapoptotic protein Bcl-2. Additionally, compound **3e** also inhibited the CDK9-mediated phosphorylation of RNA polymerase II. The molecular modeling of *pyrimeriolin* **3e** demonstrated complete integration of the sidechain into the binding pocket, whereas longer sidechains from other compounds were protruding from the binding pocket (**3i**). The butoxy sidechain exhibited an optimal sidechain length for maximized biological activity, and *pyrimeriolin* **3e** served as a lead compound for further optimization of the *pyrimeriolin* structure. Additional functionalization of the alkoxy sidechain seemed promising for maximizing interactions in the enzyme pocket.

## Data Availability

Data were generated by the authors and are included in the article and the Supporting Information.

## Supplementary Materials

The following supporting information can be downloaded at https://www.mdpi.com/article/10.3390/molecules29246050/s1, synthetic procedures, analytics, and ^1^H and ^13^C NMR spectra of precursors **1**, intermediates **2**, and title compounds **3**. References [10,14] are cited in the Supplementary Materials.
