# Supplementary material for: Novel 4-alkoxy Meriolin Congeners Potently Induce Apoptosis in Leukemia and Lymphoma Cells
Source: Molecules. 2024 Dec 23;29(24):6050. doi: 10.3390/molecules29246050 (PMC11676355; doi:10.3390/molecules29246050)
Supplement: Supplementary file 1 [file molecules-29-06050-s001.zip › molecules-3318440-supplementary.pdf]

Supporting Information

Novel 4-alkoxy meriolin congeners potently induce apoptosis in leukemia and lymphoma cells

Karina S. Krings,[1,#] Tobias R. Wassenberg,[2,#] Pablo Cea-Medina,[3] Laura Schmitt,[1] Ilka Lechtenberg,[1] Tanya R. Llewellyn,[4] Nan Qin,[4,5] Holger Gohlke,[3,6] Sebastian Wesselborg,[1,5,*] and Thomas J. J. Müller[2,*]

[1] Institute for Molecular Medicine I, Medical Faculty and University Hospital Düsseldorf, Heinrich Heine University Düsseldorf, Universitätsstraße 1, D-40225 Düsseldorf, Germany; sebastian.wesselborg@uni-duesseldorf.de (S.W.), Karina.Krings@uni-duesseldorf.de (K.S.K.), Laura-schmitt9@web.de (L.S.), Ilka.Hinxlage@uni-duesseldorf.de (I.L.)

[2] Institute of Organic Chemistry and Macromolecular Chemistry, Faculty of Mathematics and Natural Sciences, Heinrich Heine University Düsseldorf, Universitätsstraße 1, D-40225 Düsseldorf, Germany; ThomasJJ.Mueller@uni-duesseldorf.de (T.J.J.M.), towas100@uni-duesseldorf.de (T.R.W.)

[3] Institute for Pharmaceutical and Medicinal Chemistry, Heinrich Heine University Düsseldorf, Universitätsstraße 1, D-40225 Düsseldorf, Germany; Pablo.Cea.Medina@hhu.de (P.C.-M.), Gohlke@uni-duesseldorf.de (H.G.)

[4] Department of Hematology, Oncology and Clinical Immunology, Medical Faculty and University Hospital Düsseldorf, Heinrich Heine University Düsseldorf, Moorenstraße 5, D-40225 Düsseldorf, Germany; TanyaRose.Llewellyn@med.uni-duesseldorf.de (T.R.L.); Nan.Qin@med.uni-duesseldorf.de (N.Q.)

[5] Center for Integrated Oncology Aachen-Bonn-Cologne-Düsseldorf (CIO ABCD), Düsseldorf, Universitätsklinikum Düsseldorf, Moorenstr. 5, D-40225 Düsseldorf, Germany; Nan.Qin@med.uni-duesseldorf.de (N.Q.), sebastian.wesselborg@uni-duesseldorf.de (S.W.)

[6] Institute of Bio- and Geosciences (IBG-4: Bioinformatics), Forschungszentrum Jülich GmbH, Wilhelm-Johnen-Straße, D-52425 Jülich, Germany; Gohlke@uni-duesseldorf.de (H.G.)

* Correspondence: ThomasJJ.Mueller@uni-duesseldorf.de; Tel.: +49 (0)211 81 12298 (T.J.J.M.); sebastian.wesselborg@uni-duesseldorf.de; Tel.: +49 (0)211 81 12298 (S.W.)

[#] Karina S. Krings and Tobias R. Wassenberg contributed equally to this work.

Table of Contents

1. **General Information** 3
2. **Preparation of the 4-alkoxy-substituted azaindoles 1 (GP 1)[1]** 5
2.1 4-Ethoxy-1*H*-pyrrolo[2,3-b]pyridine (1c) 8
2.2 4-Propoxy-1*H*-pyrrolo[2,3-b]pyridine (1d) 9
2.3 4-Butoxy-1*H*-pyrrolo[2,3-b]pyridine (1e) 10
2.5 4-(Hexyloxy)-1*H*-pyrrolo[2,3-b]pyridine (1g) 12
2.6 4-(Octyloxy)-1*H*-pyrrolo[2,3-b]pyridine (1h) 13
2.7 4-(Dodecyloxy)-1*H*-pyrrolo[2,3-b]pyridine (1i) 14
3. **Preparation of iodinated and tosylated azaindoles 2 (GP 2)[2]** 15
3.1 4-Chloro-3-iodo-1-tosyl-1*H*-pyrrolo[2,3-b]pyridine (2a) 19
3.2 3-Iodo-4-methoxy-1-tosyl-1*H*-pyrrolo[2,3-b]pyridine (2b) 20
3.3 4-Ethoxy-3-iodo-1-tosyl-1*H*-pyrrolo[2,3-b]pyridine (2c) 21
3.4 3-Iodo-4-propoxy-1-tosyl-1*H*-pyrrolo[2,3-b]pyridine (2d) 22
3.5 4-Butoxy-3-iodo-1-tosyl-1*H*-pyrrolo[2,3-b]pyridine (2e) 23
3.6 3-Iodo-4-(2-methoxyethoxy)-1-tosyl-1*H*-pyrrolo[2,3-b]pyridine (2f) 24
3.7 4-(Hexyloxy)-3-iodo-1-tosyl-1*H*-pyrrolo[2,3-b]pyridine (2g) 25
3.8 3-Iodo-4-(octyloxy)-1-tosyl-1*H*-pyrrolo[2,3-b]pyridine (2h) 26
3.9 4-(Dodecyloxy)-3-iodo-1-tosyl-1*H*-pyrrolo[2,3-b]pyridine (2i) 27
4. **Synthesis of *pyrimeriolins* 3 via the MBSA-sequence (GP 3)[2]** 28
4.1 4-(4-Chloro-1*H*-pyrrolo[2,3-b]pyridin-3-yl)pyridin-2-amine (3a) 32
4.2 4-(4-Methoxy-1*H*-pyrrolo[2,3-b]pyridin-3-yl)pyridin-2-amine (3b) 33
4.3 4-(4-Ethoxy-1*H*-pyrrolo[2,3-b]pyridin-3-yl)pyridin-2-amine (3c) 34
4.4 4-(4-Propoxy-1*H*-pyrrolo[2,3-b]pyridin-3-yl)pyridin-2-amine (3d) 35
4.5 4-(4-Butoxy-1*H*-pyrrolo[2,3-b]pyridin-3-yl)pyridin-2-amine (3e) 36
4.6 4-(4-(2-Methoxyethoxy)-1*H*-pyrrolo[2,3-b]pyridin-3-yl)pyridin-2-amine (3f) 37
4.7 4-(4-(Hexyloxy)-1*H*-pyrrolo[2,3-b]pyridin-3-yl)pyridin-2-amine (3g) 38
4.8 4-(4-(Octyloxy)-1*H*-pyrrolo[2,3-b]pyridin-3-yl)pyridin-2-amine (3h) 39
4.9 4-(4-(Dodecyloxy)-1*H*-pyrrolo[2,3-b]pyridin-3-yl)pyridin-2-amine (3i) 40
5. **NMR Spectra** 41
6. **References** 66

1. General Information

Unless otherwise stated, all reactions were carried out in heated Schlenk or multi-necked flasks under a nitrogen atmosphere and using septa and syringes. The solvents used were dried using the MB-SPS 800 solvent drying system from *M. Braun*. Reactions carried out at reduced temperatures were cooled with ice/water.

The column chromatography was carried out with silica gel M60 (grain size 0.040-0.063 nm) from *Macherey-Nagel*, Düren. The raw product was adsorbed on Celite®545 from *Carl Roth GmbH*, then applied to the slurry of silica gel and separated by column chromatography using flash technique at an overpressure of approx. 2 bar of compressed air. Mixtures of distilled solvents were used as eluents. The distilled solvents used include dichloromethane, *n*-hexane and acetone.

Silica-coated aluminum foils (60 F_{254} *Merck*, Darmstadt) were used to carry out thin-layer chromatography. Detection was carried out using UV light (λ = 254 nm).

All commercially available chemicals were purchased from *BLD Pharmatech GmbH, Carbolution, Fisher Scientific*, *J&K Scientific GmbH, Merck*, *Roth, Sigma-Aldrich*, *VWR*, and used without further purification.

BLD Pharmatech GmbH: 4-hydroxy-7-azaindole (97%), *n*-octanol (98%), dodecyl alcohol (98%); *Carbolution*: 2-amine-4-bromopyridine (98%); *Fisher Scientific:* sodium hydroxide (AR), potassium hydroxide (AR), propan-1-ol (LR), butan-1-ol (AR), dimethylformamide (AR)); *J&K Scientific GmbH: para*-toluenesulfonyl chloride (99%); *Merck:* ethylene glycol monomethyl ether (p.a.); *Roth:* iodine (99.5%), ethanol (>99.5%);

Sigma-Aldrich: tetrakis(triphenylphosphane) palladium(0) (99%), cesium carbonate (99%), 1-hexanol (95%), diethyl azodicarboxylate solution 40% in toluene (purum), triphenylphosphane (99%), *VWR:* ammonia 25% (AR), methanol (AR).

^{1}H, ^{13}C and 135-DEPT NMR spectra were recorded on *Avance DRX 500*, *AV III 600* or *AV III 300* instruments from *Bruker*. The deuterated solvent used was always used as a standard. DMSO-d_6 (δ_H 2.49, δ_C 39.5) was primarily used.

The spin multiplicities were abbreviated as s (singlet), d (doublet), t (triplet), q (quartet), dd (doublet of a doublet), ddt (doublet of a doublet of a triplet), dq (doublet of a quartet) and m (multiplet). Assignment of methyl (CH_3), methylene (CH_2), methine (CH) and quaternary carbon nuclei was made using 135-DEPT spectra.

All mass spectra were measured in the department of mass spectroscopy at Heinrich-Heine-University (HHuCeMSA). The ESI HRMS-spectra were measured on the *UHR-QTOF maXis 4G* device from *Bruker Daltonics*. All EI spectra were measured on the *JEOL JMS-Q1600GC single-quad mass spectrometer*. All peaks with an intensity >10% of the base peak and the molecular peak were indicated.

IR-spectra were recorded using the *IRAffinity-1* device from *Shimadzu*. The measurements were carried out using a single reflection ATR unit with a diamond ATR crystal. The intensities of the absorption bands are divided into s (strong), m (medium) and w (weak).

All melting points were measured using a *Melting Point B-540* from *Büchi* using the Kofler method.

The elemental analyses were carried out at the microanalytical laboratory of the Institute of Pharmaceutical Chemistry at Heinrich Heine University. The measurements were carried out using the *Perkin Elmer Series II Analyzer 2400* device or a *Vario Micro Cube* from *Analysenssysteme GmbH*.

2. Preparation of the 4-alkoxy-substituted azaindoles 1 (GP 1)[1]

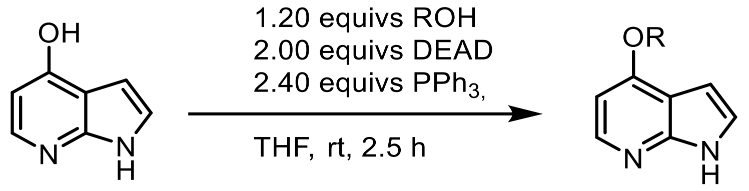

4-Hydroxy-7-azaindole (671 mg, 5.00 mmol) and the corresponding alcohol (6.00 mmol) were placed in an evacuated Schlenk tube with a magnetic stir bar and dissolved in dry THF (50 mL). In parallel, triphenylphosphane (3.15 g, 12.0 mmol) was placed in a 50 mL round-bottom flask with a septum. After evacuation and filling the vessel with nitrogen three times, a solution of azodicarboxalate (1.74 g, 10.0 mmol) in toluene was added, followed by dry THF (30 mL). This solution was then added to the dissolved 4-hydroxy-7-azaindole solution and stirred at room temperature for 2.5 h. After the reaction was completed, the solvent was removed in *vacuo* and the residue adsorbed on Celite®. After isolation by column chromatography on silica gel (98:2 dichloromethane/methanol) the product was further purified by recrystallization from dichloromethane, furnishing the desired compounds **7** as colorless crystals in yields between 36-61% (For experimental details, see **Table 1**).

Table 1: Overview of the alcohols used and the yields of the synthesized azaindoles **1**.

**Entry**	**Starting material**<br>**[Amount]**	**Product 7**<br>**[Yield]**
**1**	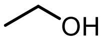<br>[276 mg, 6.00 mmol]	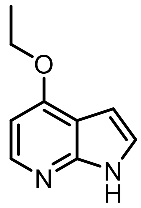<br>**1c**<br>37 %<br>[302 mg, 1.86 mmol]
**2**	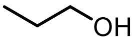<br>[361 mg, 6.00 mmol]	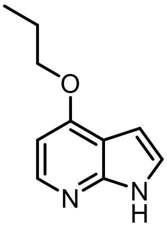<br>**1d**<br>53 %<br>[471 mg, 2.67 mmol]
**3**	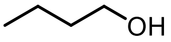<br>[445 mg, 6.00 mmol]	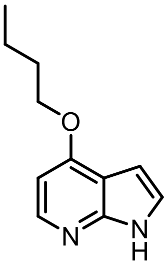<br>**1e**<br>36 %<br>[342 mg, 1.80 mmol]
**4**	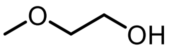<br>[457 mg, 6.00 mmol]	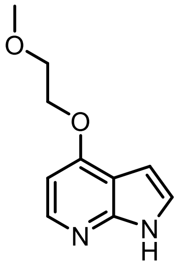<br>**1f**<br>44 %<br>[421 mg, 2.19 mmol]

Continuation Table 1: Overview of the alcohols used and the yields of the synthesized azaindoles **1**.

**Entry**	**Starting material [Amount]**	**Product 1 [Yield]**
**5**	*n*-Hex–OH [613 mg, 6.00 mmol]	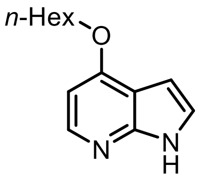 **1g** 52 % [569 mg, 2.61 mmol]
**6**	*n*-Oct–OH [781 mg, 6.00 mmol]	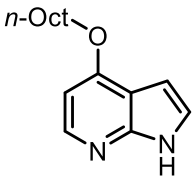 **1h** 61 % [757 mg, 3.07 mmol]
**7**	*n*-Dodec–OH [1118 mg, 6.00 mmol]	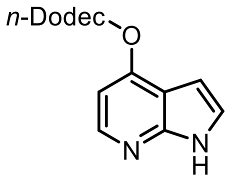 **1i** 52 % [784 mg, 2.59 mmol]

2.1 4-Ethoxy-1*H*-pyrrolo[2,3-b]pyridine (1c)

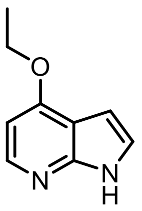

1c
[162.19]
$C_9H_{10}N_2O$

According to GP 1 product **1c** (302 mg, 1.86 mmol, 37%) was obtained as colorless crystals.

Mp 177 °C

R_f (dichloromethane/methanol (98:2)): 0.36.

^{1}H NMR (DMSO-d$_6$, 300 MHz): δ 1.40 (t, $^3J_{HH}$ = 7.0 Hz, 3H), 4.23 (q, $^3J_{HH}$ = 7.0 Hz, 2H), 6.40 (dd, $^3J_{HH}$ = 3.5 Hz, $^5J_{HH}$ = 1.5 Hz, 1H), 6.60 (d, $^3J_{HH}$ = 5.5 Hz, 1H), 7.25 (dd, $^3J_{HH}$ = 3.4 Hz, $^5J_{HH}$ = 2.1 Hz, 1H), 8.06 (d, $^3J_{HH}$ = 5.5 Hz, 1H), 11.52 (s, 1H).

^{13}C NMR (DMSO-d$_6$, 75 MHz): δ 14.58 (CH_3), 63.33 (CH_2), 97.07 (CH), 98.29 (CH), 109.62 (C_{quat}), 123.30 (CH), 144.48 (CH), 150.30 (C_{quat}), 158.17 (C_{quat}).

IR (neat): $\tilde{\nu}$ [cm^{-1}]: 3113 (w), 3090 (w), 3067 (w), 2995 (w), 2984 (w), 2974 (w), 2943 (w), 2880 (w), 2835 (w), 2778 (w), 1884 (w), 1581 (m), 1508 (m), 1491 (w), 1452 (w), 1443 (w), 1420 (m), 1389 (m), 1348 (m), 1331 (m), 1300 (m), 1275 (m), 1215 (m), 1144 (m), 1111 (m), 1094 (m), 1051 (m), 1016 (m), 943 (w), 895 (w), 868 (m), 824 (s), 800 (s), 783 (s), 721 (s), 654 (m), 637 (s).

MS (EI, *m/z* (%)): 163 (13), 162 ($[M]^+$, 100), 134 ($[C_7H_6N_2O]^+$, 96), 133 ($[C_7H_5N_2O]^+$, 12), 107 ($[C_6H_5NO]^+$, 10), 106 ($[C_6H_4NO]^+$, 14), 105 ($[C_6H_3NO]^+$, 37), 79 ($[C_5H_5N]^+$, 18), 78 ($[C_5H_4N]^+$, 11), 63 ($[C_5H_3]^+$, 10), 52 (14).

2.2 4-Propoxy-1*H*-pyrrolo[2,3-b]pyridine (1d)

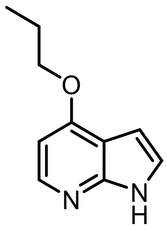

1d
[176.22]
$C_{10}H_{12}N_2O$

According to GP 1 product **1d** (302 mg, 2.67 mmol, 53%) was obtained as colorless crystals.

Mp 167 °C.

R_f (dichloromethane/methanol (98:2)): 0.42.

^{1}H NMR (DMSO-d$_6$, 300 MHz): δ 1.01 (t, $^3J_{HH}$ = 7.4 Hz, 3H), 1.80 (tq, $^3J_{HH}$ = 7.5 Hz, $^3J_{HH}$ = 6.5 Hz, 2H), 4.12 (t, $^3J_{HH}$ = 6.5 Hz, 2H), 6.41 (dd, $^3J_{HH}$ = 3.5 Hz, $^5J_{HH}$ = 1.4 Hz, 1H), 6.60 (d, $^3J_{HH}$ = 5.5 Hz, 1H), 7.25 (dd, $^3J_{HH}$ = 3.4 Hz, $^5J_{HH}$ = 1.9 Hz, 1H), 8.06 (d, $^3J_{HH}$ = 5.5 Hz, 1H), 11.52 (s, 1H).

^{13}C NMR (DMSO-d$_6$, 75 MHz): δ 10.36 (CH_3), 21.98 (CH_2), 69.05 (CH_2), 97.05 (CH_3), 98.35 (CH_3), 109.67 (C_{quat}), 123.29 (CH_3), 144.48 (CH_3), 150.30 (C_{quat}), 158.33 (C_{quat}).

IR (neat): $\tilde{\nu}$ [cm^{-1}]: 3188 (w), 3136 (w), 3084 (w), 2997 (w), 2967 (w), 2934 (w), 2876 (w), 2843 (w), 2779 (w), 1749 (w), 1694 (w), 1611 (w), 1584 (m), 1510 (w), 1499 (w), 1472 (w), 1449 (w), 1439 (w), 1420 (w), 1389 (w), 1350 (w), 1333 (m), 1294 (s), 1276 (w), 1219 (w), 1190 (w), 1148 (w), 1101 (m), 1084 (w), 1057 (m), 970 (m), 901 (w), 849 (m), 799 (m), 783 (m), 766 (m), 723 (s), 696 (w), 650 (w), 635 (s) .

MS (EI, *m/z* (%)): 176 ($[M]^+$, 57), 134 ($[C_7H_6N_2O]^+$, 100), 105 ($[C_6H_3NO]^+$, 18).

2.3 4-Butoxy-1*H*-pyrrolo[2,3-b]pyridine (1e)

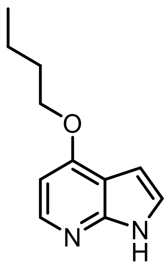

1e
[190.25]
$C_{11}H_{14}N_2O$

According to GP 1 product **1e** (342 mg, 1.80 mmol, 36%) was obtained as colorless crystals.

Mp 179 °C.

R_f (dichloromethane/methanol (98:2)): 0.36.

^{1}H NMR (DMSO-d$_6$, 300 MHz): δ 0.95 (t, $^3J_{HH}$ = 7.4 Hz, 3H), 1.28 – 1.57 (m, 2H), 1.64 – 1.86 (m, 2H), 4.17 (t, $^3J_{HH}$ = 6.5 Hz, 2H), 6.40 (dd, $^3J_{HH}$ = 3.6 Hz, $^5J_{HH}$ = 1.6 Hz, 1H), 6.61 (d, $^3J_{HH}$ = 5.5 Hz, 1H), 7.25 (dd, $^3J_{HH}$ = 3.5 Hz, $^5J_{HH}$ = 2.0 Hz, 1H), 8.06 (d, $^3J_{HH}$ = 5.5 Hz, 1H), 11.51 (s, 1H).

^{13}C NMR (DMSO-d$_6$, 75 MHz): δ 13.67 (CH_3), 18.71 (CH_2), 30.61 (CH_2), 67.31 (CH_2), 97.01 (CH), 98.31 (CH), 109.65 (C_{quat}), 123.25 (CH), 144.45 (CH), 150.26 (C_{quat}), 158.30 (C_{quat}).

IR (neat): $\tilde{\nu}$ [cm^{-1}]: 3094 (w), 3065 (w), 2870 (w), 2837 (w), 2778 (w), 1580 (m), 1558 (w), 1510 (m), 1464 (w), 1422 (w), 1348 (m), 1333 (m), 1300 (m), 1271 (m), 1144 (m), 1094 (m), 947 (m), 905 (m), 870 (s), 831 (m), 802 (s), 785 (s), 750 (m), 719 (s), 652 (m), 637 (s).

MS (EI, *m/z* (%)): 190 ($[M]^+$, 61), 134 ($[C_7H_6N_2O]^+$, 100), 105 ($[C_6H_3NO]^+$, 18).

Anal. calcd. for $C_{11}H_{14}N_2O$ [190.25]: C 69.45, H 7.42, N 14.73; **Found:** C 69.42, H 7.15, N 14.51.

2.4 4-(2-Methoxyethoxy)-1*H*-pyrrolo[2,3-b]pyridine (1f)

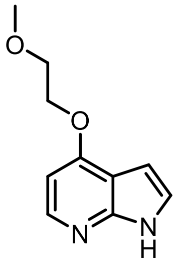

1f
[192.22]
$C_{10}H_{12}N_2O_2$

According to GP 1 product **1f** (421 mg, 2.19 mmol, 44%) was obtained as colorless crystals.

Mp 159 °C.

R_f (dichloromethane/methanol (98:2)): 0.39.

^{1}H NMR (DMSO-d$_6$, 300 MHz): δ 3.34 (s, 5H), 3.58 – 3.85 (m, 2H), 4.19 – 4.51 (m, 2H), 6.41 (dd, $^3J_{HH}$ = 3.4 Hz, $^3J_{HH}$ = 1.7 Hz, 1H), 6.63 (d, $^3J_{HH}$ = 5.6 Hz, 1H), 7.27 (dd, $^3J_{HH}$ = 3.5 Hz, $^5J_{HH}$ = 2.2 Hz, 1H), 8.06 (d, $^3J_{HH}$ = 5.7 Hz, 1H), 11.54 (s, 1H).

^{13}C NMR (DMSO-d$_6$, 75 MHz): δ 58.30 (CH_3), 67.20 (CH_2), 70.23 (CH_2), 97.06 (CH), 98.38 (CH), 109.59 (C_{quat}), 123.40 (CH), 144.44 (CH), 150.32 (C_{quat}), 158.14 (C_{quat}).

IR (neat): $\tilde{\nu}$ [cm^{-1}]: 3098 (w), 3071 (w), 2878 (w), 2837 (w), 2787 (w), 1589 (m), 1512 (m), 1449 (m), 1335 (m), 1296 (s), 1275 (m), 1238 (w), 1198 (w), 1148 (m), 1128 (m), 1107 (m), 1092 (m), 1055 (m), 1036 (m), 1022 (w), 893 (s), 860 (s), 839 (s), 824 (m), 800 (s), 781 (m), 721 (s), 712 (s), 640 (s).

MS (EI, *m/z* (%)): 193 (12), 192 ([M]$^+$, 74), 135 ([$C_7H_7N_2O$]$^+$, 10), 134 ([$C_7H_6N_2O$]$^+$, 100), 117 ([$C_7H_5N_2$]$^+$, 16), 105 ([C_6H_3NO]$^+$, 20), 59 ([C_3H_7O]$^+$, 25).

2.5 4-(Hexyloxy)-1*H*-pyrrolo[2,3-b]pyridine (1g)

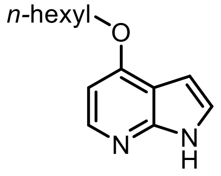

1g
[218.30]
$C_{13}H_{18}N_2O$

According to GP 1 product **1g** (569 mg, 2.61 mmol, 52%) was obtained as colorless crystals.

Mp 156 °C.

R_f (dichloromethane/methanol (98:2)): 0.21.

^{1}H NMR (DMSO-d$_6$, 600 MHz): δ 0.61 – 1.04 (m, 3H), 1.04 – 1.37 (m, 4H), 1.38 – 1.56 (m, 2H), 1.78 (m, 2H), 4.16 (t, $^3J_{HH}$ = 6.5 Hz, 2H), 6.39 (dd, $^3J_{HH}$ = 3.5 Hz, $^4J_{HH}$ = 1.7 Hz, 1H), 6.60 (d, $^3J_{HH}$ = 5.5 Hz, 1H), 7.25 (dd, $^3J_{HH}$ = 3.4 Hz, $^3J_{HH}$ = 2.2 Hz, 1H), 8.05 (d, $^3J_{HH}$ = 5.5 Hz, 1H), 11.51 (s, 1H).

^{13}C NMR (DMSO-d$_6$, 150 MHz): δ 13.89 (CH_3), 22.05 (CH_2), 25.15 (CH_2), 28.50 (CH_2), 30.96 (CH_2), 67.62 (CH_2), 97.01 (CH), 98.34 (CH), 109.66 (C_{quat}), 123.28 (CH), 144.47 (CH), 150.28 (C_{quat}), 158.31 (C_{quat}).

IR (neat): $\tilde{\nu}$ [cm^{-1}]: 3194 (w), 3119 (w), 3093 (w), 3067 (w), 2930 (w), 2856 (w), 2841 (w), 2780 (w), 2615 (w), 1585 (m), 1510 (w), 1491 (w), 1466 (w), 1422 (w), 1389 (w), 1333 (m), 1302 (m), 1273 (m), 1144 (m), 1094 (m). 982 (w), 947 (w), 918 (m), 901 (w), 864 (m), 826 (m), 802 (s), 785 (m), 719 (s), 652 (m), 638 (m).

MS (EI, *m/z* (%)): 218 ($[M]^+$, 7), 134 ($[C_7H_6N_2O]^+$, 19), 59 ($[C_4H_{11}]^+$, 25), 58 ($[C_4H_{10}]^+$, 100).

Anal. calcd. for $C_{11}H_{14}N_2O$ [218.30]: C 71.53, H 8.31, N 12.83; **Found:** C 71.88, H 8.10, N 12.86.

2.6 4-(Octyloxy)-1*H*-pyrrolo[2,3-b]pyridine (1h)

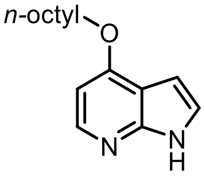

1h
[246.35]
$C_{15}H_{22}N_2O$

According to GP 1 product **1h** (757 mg, 3.07 mmol, 61%) was obtained as colorless crystals.

Mp 145 °C.

R_f (Dichlormethan/Methanol (7:3)): 0.38.

^{1}H NMR (DMSO-d$_6$, 300 MHz): δ 0.73 – 0.92 (m, 3H), 1.18 – 1.51 (m, 10H), 1.78 (p, $^3J_{HH}$ = 6.7 Hz, 2H), 4.15 (t, $^3J_{HH}$ = 6.5 Hz, 2H), 6.39 (dd, $^3J_{HH}$ = 3.4 Hz, $^4J_{HH}$ = 1.7 Hz, 1H), 6.60 (d, $^3J_{HH}$ = 5.5 Hz, 1H), 7.25 (dd, $^3J_{HH}$ = 3.4 Hz, $^3J_{HH}$ = 2.2 Hz, 1H), 8.05 (d, $^3J_{HH}$ = 5.4 Hz, 1H), 11.51 (s, 1H).

^{13}C NMR (DMSO-d$_6$, 75 MHz): δ 13.92 (CH_3), 22.05 (CH_2), 25.46 (CH_2), 28.50 (CH_2), 28.62 (CH_2), 28.68 (CH_2), 31.19 (CH_2), 67.59 (CH_2), 96.98 (CH), 98.30 (CH), 109.64 (C_{quat}), 123.25 (CH), 144.44 (CH), 150.24 (C_{quat}), 158.28 (C_{quat}).

IR (neat): $\tilde{\nu}$ [cm^{-1}]: 3119 (w), 3092 (w), 3065 (w), 2994 (w), 2949 (w), 2927 (m), 2778 (w), 1583 (m), 1510 (w), 1494 (w), 1466 (m), 1422 (w), 1399 (m), 1333 (m), 1300 (m) 1271 (s), 1217 (w), 1144 (m), 1094 (m), 1063 (w), 1043 (m), 1005 (w), 947 (m), 893 (m), 860 (m), 829 (m), 802 (s), 785 (m), 764 (w), 719 (s), 652 (w), 638 (m).

MS (EI, *m/z* (%)): 246 ($[M]^+$, 31), 134 ($[C_7H_6N_2O]^+$, 100).

ESI HRMS calc. für $[C_{15}H_{23}N_2O]^+$: 247.1805; **Found:** 247.1809.

HPLC t_r: 3.7 min (>99 % purity).

2.7 4-(Dodecyloxy)-1*H*-pyrrolo[2,3-b]pyridine (1i)

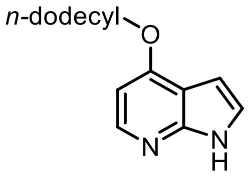

1i
[302.46]
$C_{19}H_{30}N_2O$

According to GP 1 product **1i** (784 mg, 2.59 mmol, 52%) was obtained as colorless crystals.

Mp 135 °C.

R_f (dichloromethane/methanol (7:3)): 0.40.

^{1}H NMR (DMSO-d$_6$, 300 MHz): δ 0.86 (t, $^3J_{HH}$ = 6.9 Hz, 3H), 1.12 – 1.31 (m, 16H), 1.39 – 1.50 (m, 2H), 1.69 – 1.86 (m, 2H), 4.18 (t, $^3J_{HH}$ = 6.5 Hz, 2H), 6.40 (d, $^3J_{HH}$ = 3.4 Hz, 1H), 6.59 (d, $^3J_{HH}$ = 5.5 Hz, 1H), 7.22 (d, $^3J_{HH}$ = 3.5 Hz, 1H), 8.05 (d, $^3J_{HH}$ = 5.4 Hz, 1H), 11.36 (s, 1H).

^{13}C NMR (DMSO-d$_6$, 75 MHz): δ 14.29 (CH_3), 22.47 (CH_2), 25.92 (CH_2), 29.05 (CH_2), 29.08 (CH_2), 29.14 (CH_2), 29.35 (CH_2), 29.36 (CH_2), 29.41 (CH_2), 29.42 (CH_2), 31.70 (CH_2), 68.24 (CH_2), 97.53 (CH), 98.99 (CH), 110.29 (C_{quat}), 123.65 (CH), 144.90 (CH), 150.92 (C_{quat}), 158.89 (C_{quat}).

IR (neat): $\tilde{\nu}$ [cm^{-1}]: 3094 (w), 3067 (w), 2995 (w), 2951 (w), 2918 (m), 2849 (m), 2779 (w), 1587 (m), 1510 (w), 1491 (w); 1466 (m), 1420 (w), 1389 (m), 1335 (m), 1302 (m), 1273 (s), 1218 (w), 1144 (m), 1092 (m), 1067 (w), 1047 (m), 986 (w), 947 (w), 903 (w), 870 (m), 844 (m), 829 (m), 802 (m), 785 (m), 719 (s), 652 (w), 638 (m).

ESI HRMS calcd. for $[C_{19}H_{31}N_2O]^+$: 303.2431; **Found:** 303.2427.

HPLC t_r: 5.4 min (>99 % purity).

3. Preparation of iodinated and tosylated azaindoles 2 (GP 2)[2]

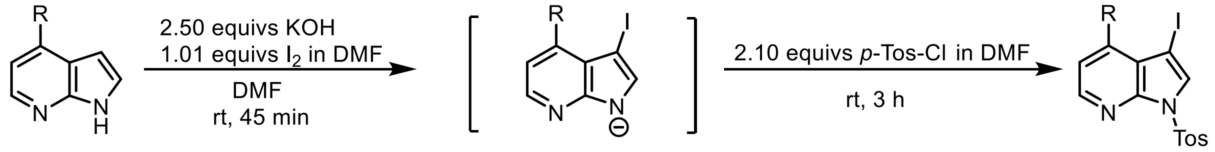

The appropriately substituted azaindole **1** (5.00 mmol) and finely crushed potassium hydroxide (701 mg, 12.5 mmol) were dissolved in *N*,*N*-dimethylformamide (30 mL) in a 250 mL round-bottom flask with a dropping funnel and magnetic stir bar. The mixture was then stirred for 10 min. Afterwards a solution of iodine (1.28 g, 5.05 mmol) in *N*,*N*-dimethylformamide (30 mL) was added to the dropping funnel and added to the reaction mixture at room temperature over 10 min. After the addition is completed, the reaction mixture was stirred at room temperature for 45 min. A solution of *para*-toluenesulfonyl chloride (2.00 g, 10.5 mmol) in *N*,*N*-dimethylformamide (30 mL) was then added dropwise at 0 °C over 20 min. The reaction mixture was then stirred at room temperature for 3 h. After completed reaction cooled thiosulfate solution (100 mL) was added dropwise to the solution and the mixture was stirred at 0 °C for another 30 min. The dropping funnel was exchanged for a shive and the solution stored in a refrigerator for 18 h. The precipitate was filtered and washed with ice water. The crude product was then dissolved in dichloromethane, adsorbed on Celite® and isolated by column chromatography on silica gel (9:1 *n*-hexane/aceton). After drying in *vacuo* at 80 °C for 8 h the desired compounds **2** were isolated as solids with yields between 46- 89% (For experimental details, see **Table 2**).

Table 2: Overview of the synthesized iodinated and tosylated azaindoles **2**.

**Entry**	**Azaindole 1** **[Amount]**	**Product 2** **[Yield]**
**1**	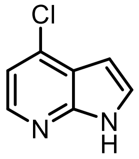 **1a** [763 mg, 5.00 mmol]	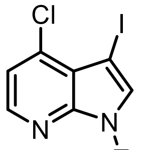 **2a** 64 % [1390 mg, 3.21 mmol]
**2**	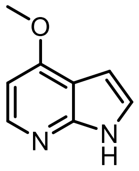 **1b** [741 mg, 5.00 mmol]	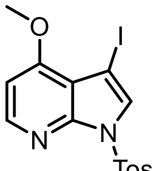 **2b** 57 % [1221 mg, 2.85 mmol]
**3**	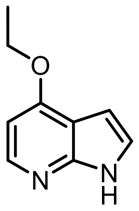 **1c** [298 mg,1.84 mmol]	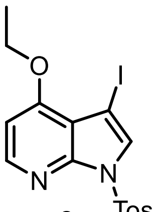 **2c** 46 % [375 mg, 0.85 mmol]

Continuation Table 2: Overview of the synthesized iodinated and tosylated azaindoles **2**.

Entry	Azaindole 1<br>[Amount]	Product 2<br>[Yield]
**4**	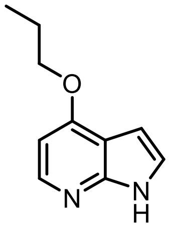<br>**1d**<br>[440 mg, 2.47 mmol]	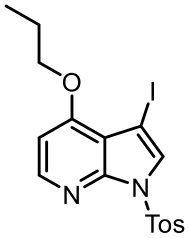<br>**2d**<br>62 %<br>[695 mg, 1.52 mmol]
**5**	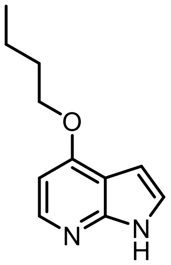<br>**1e**<br>[298 mg, 1.57 mmol]	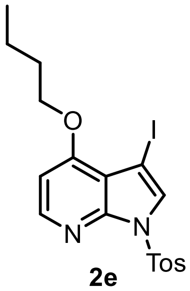<br>**2e**<br>84 %<br>[604 mg, 1.28 mmol]
**6**	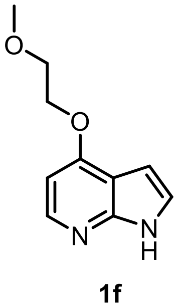<br>**1f**<br>[420 mg, 2.16 mmol]	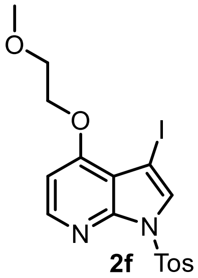<br>**2f**<br>51 %<br>[502 mg, 1.06 mmol]

Continuation Table 2: Overview of the synthesized iodinated and tosylated azaindoles **2**.

**Entry**	**Azaindole 1**<br>**[Amount]**	**Product 2**<br>**[Yield]**
**7**	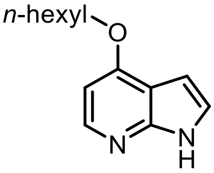<br>**1g**<br>[512 mg, 2.35 mmol]	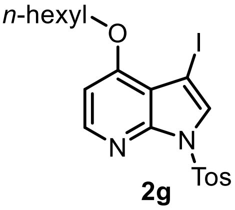<br>**2g**<br>89 %<br>[1040 mg, 2.09 mmol]
**8**	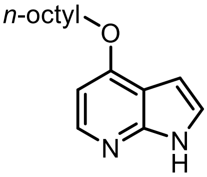<br>**1h**<br>[464 mg,1.88 mmol]	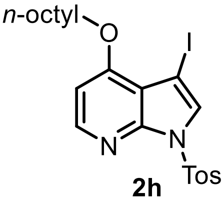<br>**2h**<br>81 %<br>[800 mg, 1.52 mmol]
**9**	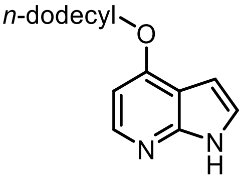<br>**1i**<br>[347 mg,1.15 mmol]	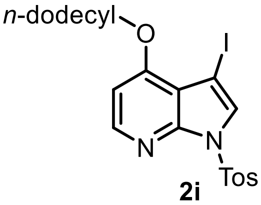<br>**2i**<br>87 %<br>[580 mg, 1.00 mmol] [1]

[1] With addition of DMAP to the *para*-toluenesulfonyl chloride solution.

3.1 4-Chloro-3-iodo-1-tosyl-1*H*-pyrrolo[2,3-b]pyridine (2a)

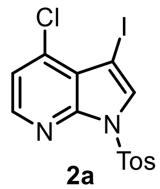

2a

[432.66]

$C_{14}H_{10}ClIN_2O_2S$

According to GP 2 product **2a** (1.22 g, 2.82 mmol, 52%) was obtained as brown solid.

Mp 190 °C.

R_f (*n*-hexane:acetone (7:3)): 0.48.

^{1}H NMR (DMSO-d$_6$, 300 MHz): δ 2.34 (s, 3H), 7.20 – 7.56 (m, 3H), 7.85 – 8.10 (m, 2H), 8.23 (s, 1H), 8.33 (d, $^3J_{HH}$ = 5.3 Hz, 1H).

^{13}C NMR (DMSO-d$_6$, 75 MHz): δ 21.13 (CH_3), 58.46 (C_{quat}), 118.97 (C_{quat}), 120.69 (CH), 127.92 (CH), 130.16 (CH), 132.27 (CH), 133.84 (C_{quat}), 136.67 (C_{quat}), 145.78 (CH), 145.91 (C_{quat}), 146.20 (C_{quat}).

IR (neat): $\tilde{\nu}$ [cm^{-1}]: 3136 (w), 2911 (w), 1649 (w), 1595 (w), 1582 (w), 1555 (m), 1493 (w), 1468 (w), 1445 (w), 1371 (m), 1356 (m), 1344 (m), 1308 (w), 1265 (w), 1229 (w), 1217 (w), 1171 (s), 1153 (m), 1121 (w), 1084 (m), 1078 (m), 1043 (m), 1018 (w), 962 (w), 937 (w), 814 (m), 799 (w), 764 (w), 710 (m), 664 (s), 642 (m), 631 (m).

MS (EI, *m/z* (%)): 434 ([^{37}ClM]$^+$, 13), 432 ([^{35}ClM]$^+$, 35), 370 ([^{37}ClIC$_9$H$_6$N$_2$O$_2$S]$^+$,16), 369 ([^{37}ClIC$_9$H$_5$N$_2$O$_2$S]$^+$,16), 368 ([^{35}ClIC$_9$H$_6$N$_2$O$_2$S]$^+$,49), 277 ([$C_7H_3ClIN_2$]$^+$, 14), 155 ([$C_7H_7O_2S$]$^+$,19), 150 ([$C_7H_3ClN_2$]$^+$, 10), 90 ([C_7H_6]$^+$, 100), 88 ([C_7H_4]$^+$, 12), 65 ([C_5H_5]$^+$,16).

3.2 3-Iodo-4-methoxy-1-tosyl-1*H*-pyrrolo[2,3-b]pyridine (2b)

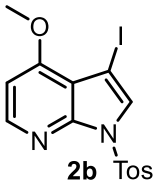

2b
[428.24]
$C_{15}H_{13}IN_2O_3S$

According to GP 2 product **2b** (1.22 g, 2.85 mmol, 57%) was obtained as beige solid.

Mp 169 °C.

R_f (*n*-hexane:acetone (7:3)): 0.46.

^{1}H NMR (DMSO-d$_6$, 300 MHz): δ 2.32 (s, 3H), 3.91 (s, 3H), 6.90 ($^3J_{HH}$, J = 5.8 Hz, 1H), 7.40 (d, $^3J_{HH}$ = 7.8 Hz, 2H), 7.88 (s, 1H), 7.98 (d, $^3J_{HH}$ = 8.5 Hz, 2H), 8.26 (d, $^3J_{HH}$ = 5.7 Hz, 1H).

^{13}C NMR (DMSO-d$_6$, 75 MHz): δ 21.11 (CH_3), 56.05 (CH_3), 57.03 (C_{quat}), 102.17 (CH), 112.10 (C_{quat}), 127.74 (CH), 128.51 (CH), 130.00 (CH), 134.21 (C_{quat}), 145.79 (C_{quat}), 147.20 (C_{quat}), 147.52 (CH), 159.98 (C_{quat}).

IR (neat): $\tilde{\nu}$ [cm^{-1}]: 3682 (w), 2988 (w), 1595 (m), 1572 (m), 1558 (w), 1489 (m), 1450 (w), 1435 (w), 1371 (s), 1325 (m), 1288 (m), 1269 (w), 1213 (m), 1180 (s), 1163 (m), 1117 (s), 1088 (s), 1015 (m), 970 (w), 955 (w), 868 (w), 808 (s), 787 (w), 719 (m), 702 (w), 685 (m), 664 (s), 625 (m).

MS (EI, *m/z* (%)): 429 (15), 428 ([M]$^+$, 75), 364 ([$C_{10}H_9IN_2O_3S$]$^+$, 54), 273 ([$C_8H_6IN_2O$]$^+$, 100), 131 ([$C_7H_3N_2O$]$^+$, 12), 125 (11), 123 (22), 116 ([$C_7H_4N_2$]$^+$, 24), 116 ([$C_7H_3N_2$]$^+$, 11), 113 (15), 111 (19), 109 (19), 101 (53), 99 (67), 97 (50), 85 (60), 58 (57).

3.3 4-Ethoxy-3-iodo-1-tosyl-1*H*-pyrrolo[2,3-b]pyridine (2c)

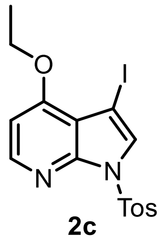

2c
[442.27]
$C_{16}H_{15}IN_2O_3S$

According to GP 2 product **2c** (375 mg, 0.85 mmol, 46%) was obtained as slight yellow solid.

Mp 150 °C.

R_f (*n*-hexane/acetone (7:3)): 0.56.

^{1}H NMR (DMSO-d$_6$, 300 MHz): δ 1.38 (t, $^3J_{HH}$ = 7.0 Hz, 3H), 2.33 (s, 3H), 4.19 (q, $^3J_{HH}$ = 7.0 Hz, 2H), 6.87 (d, $^3J_{HH}$ = 5.7 Hz, 1H), 7.40 (d, $^3J_{HH}$ = 7.9 Hz, 2H), 7.87 (s, 1H), 7.98 (d, $^3J_{HH}$ = 8.5 Hz, 2H), 8.23 (d, $^3J_{HH}$ = 5.7 Hz, 1H).

^{13}C NMR (DMSO-d$_6$, 75 MHz): δ 14.14 (CH_3), 21.09 (CH_3), 57.34 (CH_2), 64.36 (C_{quat}), 102.63 (CH), 111.87 (C_{quat}), 127.71 (CH), 128.45 (CH), 130.00 (CH), 134.23 (C_{quat}), 145.76 (C_{quat}), 147.24 (C_{quat}), 147.50 (CH), 159.16 (C_{quat}).

IR (neat): $\tilde{\nu}$ [cm^{-1}]: 3148 (w), 2988 (w), 2930 (w), 2884 (w), 1591 (w), 1572 (m), 1558 (w), 1537 (w), 1504 (w), 1487 (w), 1472 (m), 1439 (w), 1381 (m), 1372 (m), 1323 (m), 1290 (m), 1271 (w), 1209 (m), 1190 (m), 1175 (s), 1161 (m), 1101 (s), 1086 (s), 1032 (m), 1016 (w), 982 (m), 914 (w), 849 (w), 802 (m), 777 (m), 758 (w), 733 (m), 696 (m), 667 (s), 625 (s).

MS (EI, *m/z* (%)): 443 (20), 442 ([M]$^+$, 100), 378 ([$C_{11}H_{11}IN_2O_3S$]$^+$, 56), 350 ([$C_9H_8IN_2O_3S$]$^+$, 13), 288 ([$C_9H_9IN_2O$]$^+$, 11), 287 ([$C_9H_8IN_2O$]$^+$, 72), 277 (15), 260 ([$C_7H_5IN_2O$]$^+$, 13), ([$C_7H_4IN_2O$]$^+$, 96), 223 (19), 155 ([$C_7H_7O_2S$]$^+$, 11), 132 ([$C_7H_4N_2O$]$^+$, 31), 91 [(C_7H_7]$^+$, 54), 77 ([C_6H_5]$^+$, 14), 65 ([C_5H_5]$^+$, 23).

Anal. calcd. for $C_{16}H_{15}IN_2O_3S$ [442.27]: C 43.45, H 3.42, N 6.33, S 7.25; **Found:** C 43.08, H 3.47, N 6.26, S 7.60.

3.4 3-Iodo-4-propoxy-1-tosyl-1*H*-pyrrolo[2,3-b]pyridine (2d)

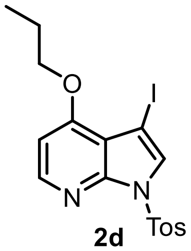

2d

[456.30]

$C_{17}H_{17}IN_2O_3S$

According to GP 2 product **2d** (695 mg, 1.52 mmol, 62%) was obtained as yellow solid.

Mp 134 °C.

R_f (*n*-hexane/acetone (7:3)): 0.39.

^{1}H NMR (DMSO-d$_6$, 300 MHz): δ 1.07 (t, $^3J_{HH}$ = 7.4 Hz, 3H), 1.61 – 1.91 (m, 2H), 2.33 (s, 3H), 4.09 (t, $^3J_{HH}$ = 6.0 Hz, 2H), 6.87 (d, $^3J_{HH}$ = 5.8 Hz, 1H), 7.40 (d, $^3J_{HH}$ = 8.7 Hz, 2H), 7.88 (s, 1H), 7.98 (d, $^3J_{HH}$ = 8.5 Hz, 2H), 8.23 (d, $^3J_{HH}$ = 5.7 Hz, 1H).

^{13}C NMR (DMSO-d$_6$, 75 MHz): δ 10.70 (CH_3), 21.09 (CH_3), 21.73 (CH_2), 57.30 (CH_2), 69.97 (C_{quat}), 102.55 (CH), 112.04 (C_{quat}), 127.70 (CH), 128.47 (CH), 129.99 (CH), 134.23 (C_{quat}), 145.75 (C_{quat}), 147.27 (C_{quat}), 147.49 (CH), 159.32 (C_{quat}).

IR (neat): $\tilde{\nu}$ [cm^{-1}]: 3136 (w), 2967 (w), 2934 (w), 2876 (w), 1657 (w), 1589 (w), 1572 (m), 1539 (w), 1489 (w), 1452 (w), 1395 (w), 1366 (m), 1327 (m), 1292 (w), 1267 (w), 1215 (w), 1190 (w), 1175 (s), 1165 (m), 1113 (s), 1088 (m), 1065 (w), 1042 (w), 1022 (w), 881 (w), 943 (w), 891 (w), 872 (w), 810 (w), 797 (w), 783 (w), 768 (w), 725 (m), 700 (m), 692 (m), 665 (S), 640 (w), 623 (s).

MS (EI, *m/z* (%)): 457 (20), 456 ([M]$^+$, 89), 392 ([$C_{12}H_{13}IN_2O_3S$]$^+$, 25), 350 ([$C_9H_7IN_2O_3S$]$^+$, 28), 301 ([$C_{10}H_{10}IN_2O$]$^+$, 29), 260 ([$C_7H_5IN_2O$]$^+$, 19), 259 ([$C_7H_4IN_2O$]$^+$, 100), 223 (19), 155 ([$C_7H_7O_2S$]$^+$, 12), 132 ([$C_7H_4N_2O$]$^+$, 18), 91 ([C_7H_7]$^+$, 56), 65 ([C_5H_5]$^+$, 21).

Anal. calcd. for $C_{17}H_{17}IN_2O_3S$ [456.30]: C 44.75, H 3.76, N 6.14, S 7.03; **Found:** C 44.58, H 3.75, N 6.06, S 7.39.

ESI HRMS calcd. for [$C_{17}H_{18}IN_2O_3S$]$^+$: 457.0077; **Found:** 457.0105.

HPLC t_r: 6.5 min (>99 % purity).

3.5 4-Butoxy-3-iodo-1-tosyl-1*H*-pyrrolo[2,3-b]pyridine (2e)

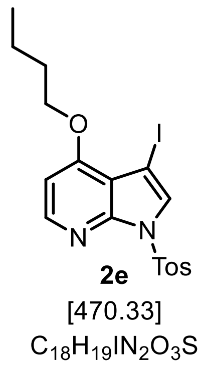

2e
[470.33]
$C_{18}H_{19}IN_2O_3S$

According to GP 2 product **2e** (604 mg, 1.28 mmol, 84%) was obtained as beige solid.

Mp 114 °C.

R_f (*n*-hexane/acetone (7:3)): 0.69.

^{1}H NMR (DMSO-d$_6$, 300 MHz): δ 0.93 (t, $^3J_{HH}$ = 7.3 Hz, 3H), 1.36 – 1.61 (m, 2H), 1.63 – 1.91 (m, 2H), 2.33 (s, 3H), 4.13 (t, $^3J_{HH}$ = 6.1 Hz, 2H), 6.88 (d, $^3J_{HH}$ = 5.8 Hz, 1H), 7.22 – 7.48 (m, 2H), 7.87 (s, 1H), 7.91 – 8.03 (m, 2H), 8.23 (d, $^3J_{HH}$ = 5.7 Hz, 1H).

^{13}C NMR (DMSO-d$_6$, 75 MHz): δ 13.60 (CH_2), 18.72 (CH_3), 21.08 (CH_2), 30.31 (CH_3), 57.29 (CH_2), 68.08 (C_{quat}), 102.56 (CH), 112.01 (C_{quat}), 127.70 (CH), 128.44 (CH), 129.98 (CH), 134.22 (C_{quat}), 145.74 (C_{quat}), 147.25 (C_{quat}), 147.47 (CH), 159.29 (C_{quat}).

IR (neat): $\tilde{\nu}$ [cm^{-1}]: 3136 (w), 3094 (w), 3028 (w), 2955 (w), 2928 (w), 2870 (w), 1595 (m), 1570 (m), 1489 (m), 1460 (w), 1369 (m), 1327 (m), 1314 (w), 1292 (m), 1215 (m), 1192 (m), 1177 (s), 1161 (m), 1113 (s), 1088 (m), 1030 (w), 1007 (m), 989 (w), 959 (w), 949 (w), 874 (w), 827 (m), 812 (m), 787 (m), 768 (w), 733 (m), 725 (w), 694 (m), 665 (s), 625 (s).

MS (EI, *m/z* (%)): 471 (19), 470 ([M]$^+$, 86), 406 ([$C_{13}H_{15}IN_2O_3S$]$^+$, 15), 350 ([$C_9H_7IN_2O_3S$]$^+$, 30), 315 ([$C_{11}H_{12I}N_2O$]$^+$,19), 279 (15), 260 ([$C_7H_5IN_2O$]$^+$, 14), 259 ([$C_7H_4IN_2O$]$^+$, 100), 223 (20), 155 ([$C_7H_7O_2S$]$^+$, 14), 132 ([$C_7H_4N_2O$]$^+$, 15), 91 ([C_7H_7]$^+$, 61), 65 ([C_5H_5]$^+$, 20), 57 (13).

Anal. calcd. for $C_{18}H_{19}IN_2O_3S$ [470.33]: C 45.97, H 4.07, N 5.96, S 6.82; **Found:** C 45.68, H 4.20, N 5.84, S 6.82.

3.6 3-Iodo-4-(2-methoxyethoxy)-1-tosyl-1*H*-pyrrolo[2,3-b]pyridine (2f)

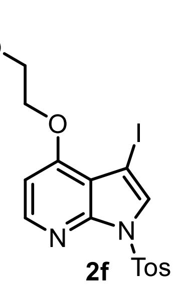

2f
[472.30]
$C_{17}H_{17}IN_2O_4S$

According to GP 2 product **2f** (502 mg, 1.06 mmol, 49%) was obtained as brown solid.

Mp 102 °C.

R_f (*n*-hexane/acetone (7:3)): 0.50.

^{1}H NMR (DMSO-d$_6$, 300 MHz): δ 2.33 (s, 3H), 3.34 (s, 3H), 3.65 – 3.80 (m, 2H), 4.21 – 4.31 (m, 2H), 6.91 (d, $^3J_{HH}$ = 5.7 Hz, 1H), 7.31 – 7.47 (m, 2H), 7.88 (s, 1H), 7.92 – 8.03 (m, 2H), 8.24 (d, $^3J_{HH}$ = 5.7 Hz, 1H).

^{13}C NMR (DMSO-d$_6$, 75 MHz): δ 21.10 (CH_3), 57.27 (CH_3), 58.40 (CH_2), 68.12 (CH_2), 69.94 (C_{quat}), 102.74 (CH), 112.02 (C_{quat}), 127.69 (CH), 128.27 (CH), 129.99 (CH), 134.21 (C_{quat}), 145.76 (C_{quat}), 147.27 (C_{quat}), 147.44 (CH), 159.15 (C_{quat}).

IR (neat): $\tilde{\nu}$ [cm^{-1}]: 3160 (w), 2935 (w), 2918 (w), 1593 (w), 1574 (m), 1489 (w), 1449 (w), 1356 (m), 1327 (m), 1294 (m), 1217 (w), 1171 (s), 1159 (m), 1132 (m), 1115 (s), 1088 (m), 1045 (w), 1016 (w), 986 (w), 949 (w), 856 (w), 806 (m), 781 (m), 737 (m), 704 (m), 665 (s), 627 (s).

MS (EI, *m/z* (%)): 473 (18), 472 ([M]$^+$, 79), 408 ([$C_{12}H_{13}IN_2O_4S$]+, 11), 350 ([$C_9H_7IN_2O_3S$]$^+$, 16), 260 ([$C_7H_5IN_2O$]$^+$, 12), 259 ([$C_7H_4IN_2O$]$^+$, 28), 254 (11), 224 ([$C_9H_8N_2O_3S$]$^+$, 19), 223 ([$C_9H_7N_2O_3S$]$^+$, 41), 190 ([$C_{10}H_{10}N_2O_2$]$^+$, 10), 155 ([$C_7H_7O_2S$]$^+$, 24), 134 ([$C_7H_6N_2O$]$^+$, 11), 91 ([C_7H_7]$^+$, 79), 65 ([C_5H_5]$^+$, 30), 63 ([C_5H_3]$^+$, 12), 59 ([C_3H_7O]$^+$, 100), 45 ([C_2H_5O]$^+$, 17).

Anal. calcd. for $C_{17}H_{17}IN_2O_4S$ [472.30]: C 43.23, H 3.63, N 5.93, S 6.79; **Found:** C 42.82, H 3.59, N 5.89, S 6.92.

ESI HRMS calcd. for [$C_{17}H_{18}IN_2O_4S$]$^+$: 473.0040; **Found:** 473.0038.

3.7 4-(Hexyloxy)-3-iodo-1-tosyl-1*H*-pyrrolo[2,3-b]pyridine (2g)

According to GP 2 product **2g** (1.04 g, 2.09 mmol, 89%) was obtained as slight yellow solid.

Mp 119 °C.

R_f (*n*-hexane/acetone (7:3)): 0.55.

^{1}H NMR (DMSO-d_6, 600 MHz): δ 0.86 (td, $^3J_{HH}$ = 5.9 Hz, $^3J_{HH}$ = 5.0 Hz, $^3J_{HH}$ = 2.9 Hz, 3H), 1.30 (h, $^3J_{HH}$ = 3.8 Hz, 4H), 1.43 – 1.58 (m, 2H), 1.66 – 1.81 (m, 2H), 2.34 (s, 3H), 4.13 (t, $^3J_{HH}$ = 6.1 Hz, 2H), 6.88 (d, $^3J_{HH}$ = 5.7 Hz, 1H), 7.41 (d, $^3J_{HH}$ = 8.3 Hz, 2H), 7.87 (s, 1H), 7.91 – 8.00 (m, 2H), 8.23 (d, $^3J_{HH}$ = 5.7 Hz, 1H).

^{13}C NMR (DMSO-d_6, 150 MHz): δ 14.40 (CH_3), 21.57 (CH_3), 22.53 (CH_2), 25.63 (CH_2), 28.68 (CH_2), 31.32 (CH_2), 57.68 (C_{quat}), 68.87 (CH_2), 103.06 (CH), 112.48 (C_{quat}), 128.18 (CH), 128.94 (CH), 130.47 (CH), 134.71 (C_{quat}), 146.23 (C_{quat}), 147.73 (C_{quat}), 147.96 (CH), 159.76 (C_{quat}).

IR (neat): $\tilde{\nu}$ [cm^{-1}]: 2945 (w), 2924 (w), 2864 (w), 1593 (m), 1568 (s), 1489 (m), 1458 (w), 1373 (s), 1325 (m), 1294 (m), 1282 (w), 1209 (m), 1179 (s), 1161 (m), 1109 (s), 1090 (s), 1038 (w), 1016 (w), 980 (w), 955 (w), 926 (w), 880 (w), 806 (m), 783 (m), 735 (w), 721 (m), 698 (m), 664 (s), 627 (s).

MS (EI, *m/z* (%)): 500 ([$H^{13}CM]^+$, 12), 499 ([HM]$^+$, 38), 498 ([M]$^+$, 94), 434 (22), 350 (32), 307 ([$C_7H_4IN_2O_2S)^+$, 28), 260 ([$C_7H_5IN_2O]^+$, 19), 259 ([$C_7H_4IN_2O]^+$, 100), 225 (12), 224 (23), 223 (26), 91 ([$C_7H_7]^+$, 80), 65 ([$C_5H_5]^+$, 20), 57 ([$C_4H_7]^+$, 16).

Anal. calcd. for $C_{20}H_{23}IN_2O_3S$ [490.38]: C 48.20, H 4.65, N 5.62, S 6.43; **Found:** C 48.33, H 4.67 N 5.55, S 6.50.

3.8 3-Iodo-4-(octyloxy)-1-tosyl-1*H*-pyrrolo[2,3-b]pyridine (2h)

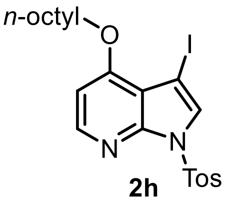

2h
[526.43]
$C_{22}H_{27}IN_2O_3S$

According to GP 2 product **2h** (800 mg, 1.52 mmol, 81%) was obtained as yellow solid.

Mp 93 °C.

R_f (*n*-hexane/acetone (7:3)): 0.60.

^{1}H NMR (DMSO-d$_6$, 300 MHz): δ 0.67 – 0.88 (m, 3H), 0.97 – 1.42 (m, 8H), 1.50 (q, $^3J_{HH}$ = 7.1 Hz, 2H), 1.76 (dd, $^3J_{HH}$ = 8.7 Hz, $^3J_{HH}$ = 5.9 Hz, 2H), 2.33 (s, 3H), 4.12 (t, $^3J_{HH}$ = 6.1 Hz, 2H), 6.88 (d, $^3J_{HH}$ = 5.7 Hz, 1H), 7.33 – 7.47 (m, 2H), 7.87 (s, 1H), 7.92 – 8.04 (m, 2H), 8.23 (d, $^3J_{HH}$ = 5.7 Hz, 1H).

^{13}C NMR (DMSO-d$_6$, 75 MHz): δ 13.92 (CH_3), 21.07 (CH_3), 22.04 (CH_2), 25.43 (CH_2), 28.35 (CH_2), 28.54 (CH_2), 28.61 (CH_2), 31.19 (CH_2), 57.15 (C_{quat}), 68.35 (CH_2), 102.55 (CH), 111.98 (C_{quat}), 127.68 (CH), 128.40 (CH), 129.95 (CH), 134.20 (C_{quat}), 145.71 (C_{quat}), 147.45 (C_{quat}), 147.22 (CH), 159.25 (C_{quat}).

IR (neat): $\tilde{\nu}$ [cm^{-1}]: 3156 (w), 2943 (w), 2918 (w), 2870 (w), 2851 (w), 1591 (m), 1568 (m), 1489 (m), 1470 (m), 1381 (m), 1325 (m), 1290 (m), 1209 (m), 1192 (m), 1175 (s), 1161 (m), 1111 (s), 1086 (m), 1040 (w), 1018 (w), 989 (w), 957 (w), 876 (w), 820 (m), 804 (m), 785 (m), 734 (m), 704 (m), 696 (m), 664 (s), 625 (s).

MS (EI, *m/z* (%)): 528 ([H^{13}CM]$^+$, 11), 527 ([HM]$^+$, 38), 526 ([M]$^+$, 100), 335 (18), 260 ([$C_7H_5IN_2O$]$^+$, 19), 223 (13), 91 ([C_7H_7]$^+$, 47), 69 (12), 57 ([C_4H_7]$^+$, 14), 55 (12).

ESI HRMS calcd. for [$C_{22}H_{28}IN_2O_3S$]$^+$: 527.0860; **Found:** 527.0868.

HPLC t_r: 8.4 min (96 % purity).

3.9 4-(Dodecyloxy)-3-iodo-1-tosyl-1*H*-pyrrolo[2,3-b]pyridine (2i)

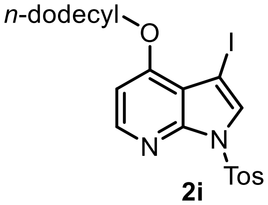

2i
[585.54]
$C_{26}H_{35}IN_2O_3S$

According to GP 2 product **2i** (520 mg, 1.00 mmol, 87%) was obtained as beige solid.

Mp 90 °C.

R_f (*n*-hexane/acetone (7:3)): 0.72.

^{1}H NMR (DMSO-d$_6$, 300 MHz): δ 0.81 – 0.85 (m, 3H), 1.17 – 1.33 (m, 16H), 1.46 – 1.53 (m, 2H), 1.71 – 1.80 (m, 2H), 4.12 (t, $^3J_{HH}$ = 6.0 Hz, 2H), 6.88 (d, $^3J_{HH}$ = 5.8 Hz, 1H), 7.32 – 7.47 (m, 2H), 7.87 (s, 1H), 7.92 – 8.03 (m, 2H), 8.23 (d, $^3J_{HH}$ = 5.7 Hz, 1H).

^{13}C NMR (DMSO-d$_6$, 75 MHz): δ 14.42 (CH_3), 21.56 (CH_3), 25.89 (CH_2), 28.68 (CH_2), 28.91 (CH_2), 29.03 (CH_2), 29.10 (CH_2), 29.15 (CH_2), 29.18 (CH_2), 29.41 (CH_2), 29.48 (CH_2), 31.76 (CH_2), 57.60 ($C_{quatquat}$), 68.83 (CH_2), 103.03 (CH), 112.48 (C_{quat}), 128.18 (CH), 128.91 (CH), 130.45 (CH), 134.71 (C_{quat}), 146.21 (C_{quat}), 147.72 (C_{quat}), 147.95 (CH), 159.75 (C_{quat}).

IR (neat): $\tilde{\nu}$ [cm^{-1}]: 2916 (m), 2849 (m), 1585 (m), 1570 (m), 1491 (m), 1468 (m), 1379 (w), 1364 (m), 1325 (m), 1288 (m), 1217 (m), 1188 (s), 1175 (s), 1165 (m), 1113 (s), 1088 (s), 1040 (w), 999 (s), 980 (w), 968 (w), 947 (w), 881 (w), 849 (w), 800 (s), 779 (w), 723 (m), 698 (m), 667 (s), 629 (s).

MS (EI, *m/z* (%)): 506 (11), 428 ($[C_{19}H_{29}IN_2O]^+$, 14), 333 (18), 332 (79), 302 ($[C_{19}H_{30}N_2O]^+$, 11), 260 ($[C_7H_5IN_2O]^+$. 51), 166 (88), 165 (37), 163 (18), 139 (13), 137 (35), 136 ($[C_7H_9N_2O]^+$, 60), 135 ($[C_7H_8N_2O]^+$, 19), 134 ($[C_7H_8N_2O]^+$, 76), 110 (24), 109 (32), 105 (11), 97 (15), 95 (12), 85 (11), 83 (24), 81 (16), 72 (16), 71 (23), 70 (13), 69 (51), 68 (20), 67 (20), 59 (15), 57 ($[C_4H_7]^+$, 69), 56 (19), 55 (100), 54 (11), 53 (12).

ESI HRMS calcd. for $[C_{26}H_{36}IN_2O_3S]^+$: 583.1486; **Found:** 583.1486.

HPLC t_r: 9.9 min (>99 % purity).

4. Synthesis of *pyrimeriolins* 3 via the MBSA-sequence (GP 3)[2]

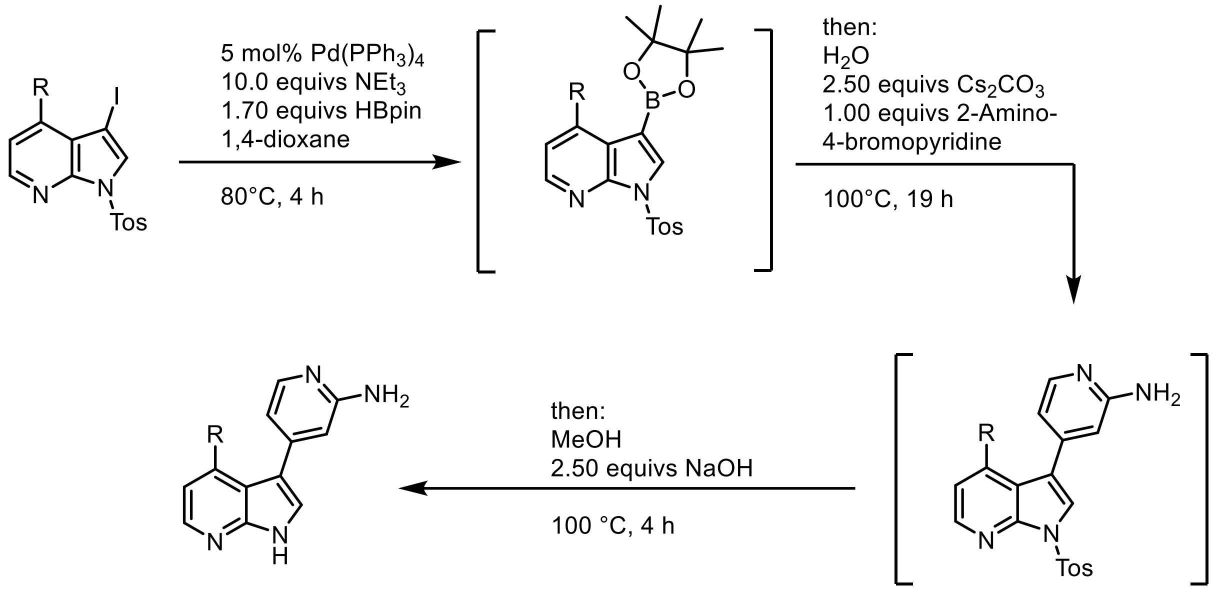

The corresponding compound **2** (1.00 mmol) was added to a dry Schlenk tube together with tetrakis(triphenylphosphane)palladium(0) (57.8 mg, 0.05 mmol) in dry 1,4-dioxane (5 mL). After degassing the solution with nitrogen for 10 min, triethylamine (1.40 mL, 100 mmol) and 4,4,5,5-tetramethyl-1,3,2-dioxaborolane (0.25 mL,1.70 mmol) were added and the solution was stirred in a preheated oil bath at 80 °C for 4 h. The reaction mixture was cooled to room temperature and distilled Water (5 mL) was carefully added. Additionally, cesium carbonate (815 mg, 2.50 mmol) and 4-amino-2-bromopyridine (173 mg, 1.00 mmol) were then added and the mixture was stirred at 100 °C for 19 h. After the reaction mixture was cooled to room temperature methanol (5 mL) and finely crushed sodium hydroxide (100 mg, 2.50 mmol) were then added to the solution before it was stirred again at 100 °C for 4 h. The solvent was removed in *vacuo* and the residue was adsorbed on Celite®. The product was isolated by column chromatography on silica gel (98:2:1 dichloromethane/methanol/ aqueous ammonia). For further purification the product was coated with *n*-hexane and treated with ultrasound. The protrusion was removed and the purified product dried in *vacuo* at 80 °C for 8 h. The *pyrimeriolins* **3** were obtained as solids with yields ranging between 52-93% (For experimental details, see **Table 3**).

Table 3: Overview of the synthesized *pyrimeriolins* **3.**

Entry	Azaindole 2<br>[Amount]	*Pyrimeriolin* 3<br>[Yield]
**1**	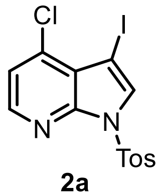<br>**2a**<br>[432 mg, 1.00 mmol]	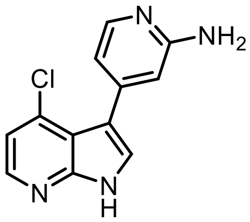<br>**3a**<br>52 %<br>[129 mg, 0.52 mmol] [2]
**2**	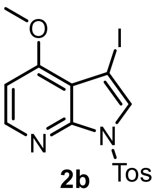<br>**2b**<br>[428 mg, 1.00 mmol]	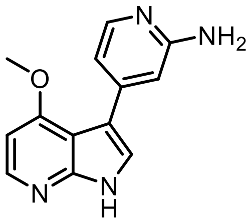<br>**3b**<br>52 %<br>[125 mg, 0.52 mmol]
**3**	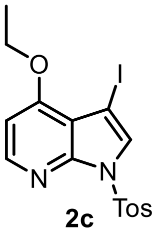<br>**2c**<br>[326 mg, 0.74 mmol]	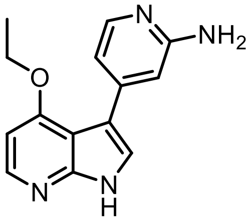<br>**3c**<br>84 %<br>[158 mg, 0.62 mmol]

[2] The reaction time of the *Masuda*-borylation was 22 h.

Continuation Table 3: Overview of the synthesized *pyrimeriolins* **3.**

**Entry**	**Azaindole 2** **[Amount]**	***Pyrimeriolin* 3** **[Yield]**
**4**	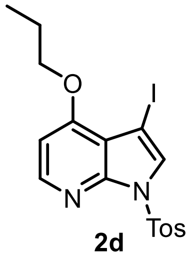 **2d** [456 mg, 1.00 mmol]	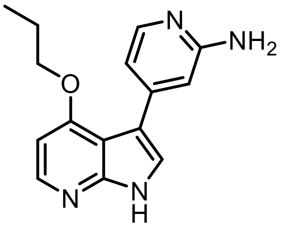 **3d** 93 % [250 mg, 0.93 mmol]
**5**	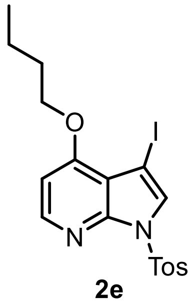 **2e** [241 mg, 0.51 mmol]	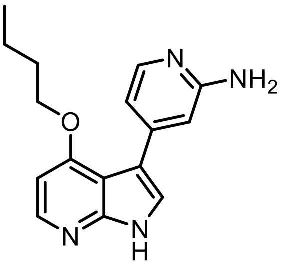 **3e** 88 % [127 mg, 0.45 mmol]
**6**	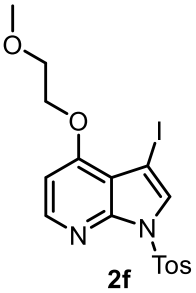 **2f** [360 mg, 0.76 mmol]	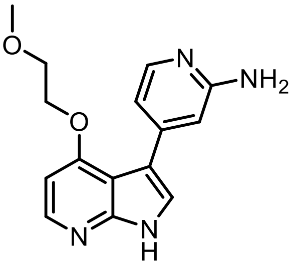 **3f** 88 % [191 mg, 0.67 mmol]

Continuation Table 3: Overview of the synthesized *pyrimeriolins* **3.**

**Entry**	**Azaindole 2** **[Amount]**	***Pyrimeriolin* 3** **[Yield]**
**7**	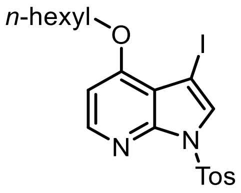 **2g** [498 mg, 1.00 mmol]	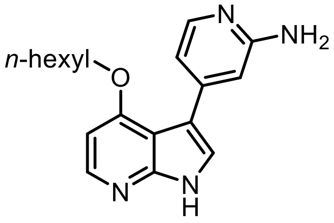 **3g** 67 % [209 mg, 0.67 mmol]
**8**	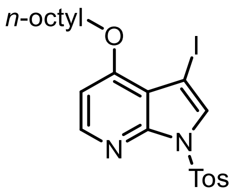 **2h** [526 mg, 1.00 mmol]	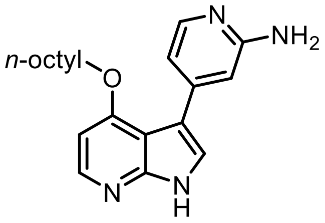 **3h** 56 % [191 mg, 0.56 mmol]
**9**	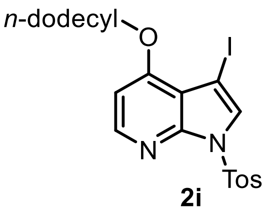 **2i** [495 mg, 0.85 mmol]	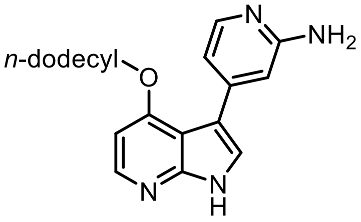 **3i** 83 % [278 mg, 0.70 mmol]

4.1 4-(4-Chloro-1*H*-pyrrolo[2,3-b]pyridin-3-yl)pyridin-2-amine (3a)

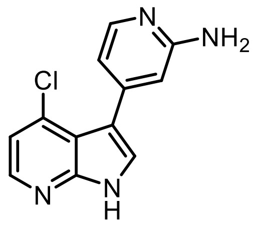

3a
[244.68]
$C_{12}H_9ClN_4$

According to GP 3 product **3a** (128 mg, 0.52 mmol, 52%) was obtained as beige solid.

Mp 239 °C.

R_f (dichloromethane/methanol/aqueous ammonia (95:5:1)): 0.13.

^{1}H NMR (DMSO-d$_6$, 300 MHz): δ 5.87 (s, 2H), 6.57 (s, 1H), 6.64 (dd, $^3J_{HH}$ = 5.3 Hz, $^4J_{HH}$ = 1.5 Hz, 1H), 7.22 (d, J = 5.1 Hz, 1H), 7.72 (d, $^3J_{HH}$ = 2.4 Hz, 1H), 7.89 (d, $^3J_{HH}$ = 5.3 Hz, 1H), 8.21 (d, $^3J_{HH}$ = 5.1 Hz, 1H), 12.36 (s, 1H).

^{13}C NMR (DMSO-d$_6$, 75 MHz): δ 108.97 (CH), 113.94 (C_{quat}), 114.16 (CH), 114.97 (C_{quat}), 117.07 (CH), 126.89 (CH), 134.37 (C_{quat}), 142.85 (C_{quat}), 143.51 (C_{quat}), 146.77 (CH), 149.72 (C_{quat}), 159.41 (C_{quat}).

IR (neat): $\tilde{\nu}$ [cm^{-1}]: 3466 (w), 3453 (w), 3302 (w), 3138 (w), 3021 (w), 2924 (w), 2868 (w), 2766 (w), 2716 (w), 2596 (w), 1639 (m), 1597 (m), 1560 (m), 1540 (m), 1522 (m), 1489 (w), 1452 (w), 1429 (m), 1400 (w), 1354 (w), 1329 (m), 1319 (m), 1296 (m), 1279 (w), 1263 (m), 1246 (w), 1192 (w), 1148 (w), 1072 (w), 1007 (w), 988 (w), 957 (w), 903 (w), 872 (m), 860 (w), 831 (m), 808 (s), 773 (m), 717 (m), 671 (m), 652 (m), 623 (s).

MS (EI, *m/z* (%)): 246 ([^{37}ClM]$^+$, 33), 245 (20), 244 ([^{35}ClM]$^+$, 100), 243 ([$C_{12}H_8ClN_4$]$^+$, 15), .

Anal. calcd. for $C_{12}H_9ClN_4$ [244.68]: C 58.91, H 3.71, N 22.90; **Found:** C 58.88, H 3.87, N 22.52.

4.2 4-(4-Methoxy-1*H*-pyrrolo[2,3-b]pyridin-3-yl)pyridin-2-amine (3b)

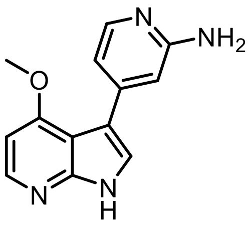

3b
[240.27]
$C_{13}H_{12}N_4O$

According to GP 3 product **3b** (125 mg, 0.52 mmol, 52%) was obtained as colorless solid.

Mp 255 °C.

R_f (dichloromethane/methanol/aqueous ammonia (95:5:1)): 0.13.

^{1}H NMR (DMSO-d$_6$, 300 MHz): δ 3.92 (s, 3H), 5.76 (s, 2H), 6.72 (d, $^3J_{HH}$ = 5.5 Hz, 1H), 6.75 – 6.84 (m, 2H), 7.58 (s, 1H), 7.84 (dd, $^3J_{HH}$ = 5.2 Hz, $^4J_{HH}$ = 0.9 Hz, 1H), 8.15 (d, $^3J_{HH}$ = 5.5 Hz, 1H), 11.94 (s, 1H).

^{13}C NMR (DMSO-d$_6$, 75 MHz): δ 55.36 (CH), 98.53 (CH), 106.75 (C_{quat}), 107.17 (CH), 112.47 (CH), 113.88 (C_{quat}), 123.45 (CH), 143.73 (C_{quat}), 145.19 (CH), 146.88 (CH), 150.90 (C_{quat}), 159.79 (C_{quat}), 159.93 (C_{quat}).

IR (neat): $\tilde{\nu}$ [cm^{-1}]: 3435 (w), 3148 (w), 3098 (w), 2841 (w), 1653 (w), 1628 (w), 1599 (m), 1578 (m), 1535 (m), 1522 (m), 1514 (w), 1479 (w), 1433 (w), 1410 (m), 1339 (w), 1319 (m), 1292 (w), 1279 (w), 1258 (m), 1150 (w), 1093 (m), 1019 (w), 989 (w), 968 (w), 880 (w), 862 (w), 849 (w), 814 (m), 802 (s), 777 (w), 741 (w), 719 (w), 696 (w), 673 (w), 650 (w), 633 (m).

MS (EI, *m/z* (%)): 241 (16), 240 ($[M]^+$, 100), 239 ($[C_{13}H_{11}NO]^+$, 31), 209 ($[C_{12}H_7N_3O]^+$, 11).

Anal. calc. for $C_{13}H_{12}N_4O$ [240.27]: C 64.99, H 5.03, N 23.32; **Found:** C 64.81, H 5.41, N 23.07.

4.3 4-(4-Ethoxy-1*H*-pyrrolo[2,3-b]pyridin-3-yl)pyridin-2-amine (3c)

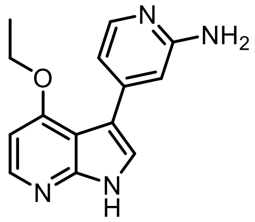

3c
[254.29]
$C_{14}H_{14}N_4O$

According to GP 3 product **3c** (158 mg, 0.62 mmol, 84%) was obtained as colorless solid.

Mp 270 °C.

R_f (dichloromethane/methanol/aqueous ammonia (95:5:1)): 0.28.

^{1}H NMR DMSO-d$_6$, 300 MHz): δ 1.40 (t, $^3J_{HH}$ = 7.0 Hz, 3H), 4.19 (q, $^3J_{HH}$ = 6.9 Hz, 2H), 5.71 (s, 2H), 6.68 (d, $^3J_{HH}$ = 5.6 Hz, 1H), 6.79 (d, $^3J_{HH}$ = 4.8 Hz, 2H), 7.55 (s, 1H), 7.75 – 7.98 (m, 1H), 8.12 (d, $^3J_{HH}$ = 5.5 Hz, 1H), 11.90 (s, 1H).

^{13}C NMR (DMSO-d$_6$, 75 MHz): δ 14.22 (CH_3), 63.49 (CH_2), 98.95 (CH), 106.71 (C_{quat}), 107.49 (CH), 112.65 (CH), 113.99 (C_{quat}), 123.24 (CH), 143.58 (C_{quat}), 145.12 (CH), 146.72 (CH), 150.96 (C_{quat}), 159.10 (C_{quat}), 159.60 (C_{quat}).

IR (neat): $\tilde{\nu}$ [cm^{-1}]: 3440 (w) 3173 (w), 3136 (w), 3092 (w), 3076 (w), 3011 (w), 2941 (w), 2886 (w), 2778 (w), 2723 (w), 2671 (w), 2604 (w), 1634 (m), 1597 (m), 1580 (m), 1543 (m), 1512 (m), 1468 (w), 1431 (m), 1402 (w), 1391 (w), 1354 (w), 1325 (m), 1302 (s), 1290 (m), 1260 (m), 1155 (m), 1109 (m), 1088 (m), 1005 (w), 991 (w), 937 (w), 895 (m), 864 (m), 847 (m), 797 (s), 775 (m), 733 (m), 708 (m), 664 (m), 648 (m), 629 (s), 611 (m).

MS (EI, *m/z* (%)): 255 (19), 254 ($[M]^+$, 100), 253 ($[C_{14}H_{13}N_4O]^+$, 15), 239 ($[C_{13}H_{11}N_4O]^+$, 16), 226 ($[C_{12}H_{10}N_4O]^+$, 226), ($[C_{12}H_9N_4O]^+$, 225), 198 ($[C_{12}H_{10}N_2O]^+$, 12), 186 ($[C_{11}H_{10}N_2O]^+$, 15).

Anal. calcd. for $C_{14}H_{14}N_4O$ [254.29]: C 66.13, H 5.55, N 22.03; **Found:** C 65.94, H 5.53, N 21.79.

4.4 4-(4-Propoxy-1*H*-pyrrolo[2,3-b]pyridin-3-yl)pyridin-2-amine (3d)

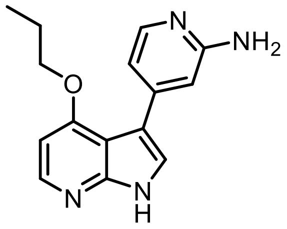

3d
[268.32]
$C_{15}H_{16}N_4O$

According to GP 3 product **3d** (250 mg, 0.62 mmol, 93%) was obtained as colorless solid.

Mp 216 °C.

R_f (dichloromethane/methanol/aqueous ammonia (95:5:1)): 0.20.

^{1}H NMR (DMSO-d$_6$, 300 MHz): δ 0.93 (t, $^3J_{HH}$ = 7.4 Hz, 3H), 1.55 – 1.95 (m, 2H), 4.08 (t, $^3J_{HH}$ = 6.4 Hz, 2H), 5.68 (s, 2H), 6.69 (d, $^3J_{HH}$ = 5.6 Hz, 1H), 6.74 (dd, $^4J_{HH}$ = 1.6 Hz, $^5J_{HH}$ = 0.8 Hz, 1H), 6.79 (dd, $^3J_{HH}$ = 5.4 Hz, $^4J_{HH}$ = 1.6 Hz, 1H), 7.53 (d, J = 1.4 Hz, 1H), 7.83 (dd, $^3J_{HH}$ = 5.4 Hz, $^5J_{HH}$ = 0.8 Hz, 1H), 8.11 (d, $^3J_{HH}$ = 5.5 Hz, 1H), 11.88 (s, 1H).

^{13}C NMR (DMSO-d$_6$, 75 MHz): δ 10.48 (CH_3), 21.78 (CH_2), 69.35 (CH_2), 98.93 (CH), 106.79 (C_{quat}), 107.32 (CH), 112.87 (CH), 114.03 (C_{quat}), 123.14 (CH), 143.70 (C_{quat}), 145.12 (CH), 146.69 (CH), 150.91 (C_{quat}), 159.30 (C_{quat}), 159.58 (C_{quat}).

IR (neat): $\tilde{\nu}$ [cm^{-1}]: 3500 (w), 3306 (w), 3194 (w), 3130 (w), 3090 (w), 3007 (w), 2963 (w), 2941 (w), 2878 (w), 2843 (w), 2729 (w), 2675 (w), 2610 (w), 1601 (m), 1582 (s), 1543 (m), 1524 (m), 1512 (m), 1485 (w), 1429 (m), 1404 (w), 1391 (w), 1358 (w), 1306 (m), 1279 (m), 1260 (m), 1240 (w), 1152 (w), 1088 (m), 1032 (w), 972 (w), 943 (m), 922 (w), 880 (w), 854 (m), 831 (m), 800 (s), 766 (m), 727 (w), 710 (w), 667 (w), 652 (w), 633 (s), 617 (m).

MS (EI, *m/z* (%)): 269 (23), 268 ($[M]^+$, 100), 227 ($[C_{12}H_{11}N_4O]^+$, 15), 226 ($[C_{12}H_{10}N_4O]^+$, 225 ($[C_{12}H_9N_4O]^+$, 44), 198 ($[C_{11}H_{10}N_4]^+$, 11).

Anal. calcd. for $C_{15}H_{16}N_4O$ [268.32]: C 67.15, H 6.01, N 20.88; **Found:** C 67.08, H 5.92, N 20.63.

4.5 4-(4-Butoxy-1*H*-pyrrolo[2,3-b]pyridin-3-yl)pyridin-2-amine (3e)

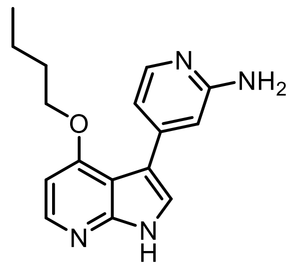

3e
[282.35]
$C_{16}H_{18}N_4O$

According to GP 3 product **3e** (127 mg, 0.45 mmol, 88%) was obtained as beige solid.

Mp 213 °C.

R_f (dichloromethane/methanol/aqueous ammonia (95:5:1)): 0.21.

^{1}H NMR (DMSO-d$_6$, 300 MHz): δ 0.89 (t, $^3J_{HH}$ = 7.4 Hz, 3H), 1.29 – 1.45 (m, 2H), 1.69 – 1.82 (m, 2H), 4.12 (t, $^3J_{HH}$ = 6.3 Hz, 2H), 5.67 (s, 2H), 6.44 – 6.86 (m, 3H), 7.52 (s, 1H), 7.82 (d, $^3J_{HH}$ = 5.4 Hz, 1H), 8.11 (d, $^3J_{HH}$ = 5.5 Hz, 1H), 11.87 (s, 1H).

^{13}C NMR (DMSO-d$_6$, 75 MHz): δ 13.65 (CH_3), 18.78 (CH_2), 30.42 (CH_2), 67.58 (CH_2) 98.91 (CH), 106.79 (C_{quat}), 107.26 (CH), 112.90 (CH), 114.01 (C_{quat}), 123.09 (CH), 143.66 (C_{quat}), 145.09 (CH), 146.61 (CH), 150.89 (C_{quat}), 159.30 (C_{quat}), 159.57 (C_{quat}).

IR (neat): $\tilde{\nu}$ [cm^{-1}]: 3327 (w), 3173 (w), 2961 (w), 2928 (w), 2716 (w), 2594 (w), 1601 (m), 1582 (m), 1545 (w), 1524 (m), 1458 (w), 1439 (w), 1412 (w), 1393 (w), 1314 (m), 1292 (w), 1275 (w), 1263 (m), 1155 (w), 1123 (w), 1090 (m), 999 (w), 964 (w), 895 (w), 854 (m), 827 (m), 812 (m), 800 (m), 775 (w), 739 (m), 698 (w), 669 (w), 652 (w), 635 (s), 613 (m).

MS (EI, *m/z* (%)): 283 (18), 282 ($[M]^+$, 78), 227 ($[C_{12}H_{11}N_4O]^+$, 17), 226 ($[C_{12}H_{10}N_4O]^+$, 100), 198 ($[C_{11}H_{10}N_4]^+$, 11).

ESI HRMS calcd. for $[C_{16}H_{19}N_4O]^+$: 283.1559; **Found:** 283.1534.

HPLC t_r: 1.5 min (>99 % purity).

4.6 4-(4-(2-Methoxyethoxy)-1*H*-pyrrolo[2,3-b]pyridin-3-yl)pyridin-2-amine (3f)

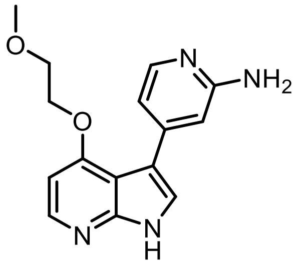

3f
[284.32]
$C_{15}H_{16}N_4O_2$

According to GP 3 product **3f** (191 mg, 0.67 mmol, 88%) was obtained as colorless solid.

Mp 213 °C.

R_f (dichloromethane/methanol/aqueous ammonia (95:5:1)): 0.22.

^{1}H NMR (DMSO-d$_6$, 300 MHz): δ 3.29 (s, 3H), 3.61 – 3.88 (m, 2H), 4.10 – 4.42 (m, 2H), 5.64 (s, 2H), 6.71 (d, $^3J_{HH}$ = 5.5 Hz, 1H), 6.83 (dd, $^4J_{HH}$ = 1.6 Hz, $^5J_{HH}$ = 0.8 Hz, 1H), 6.87 (dd, $^3J_{HH}$ = 5.4 Hz, $^4J_{HH}$ = 1.6 Hz, 1H), 7.59 (d, $^4J_{HH}$ = 1.4 Hz, 1H), 7.84 (dd, $^3J_{HH}$ = 5.4 Hz, $^5J_{HH}$ = 0.8 Hz, 1H), 8.13 (d, $^3J_{HH}$ = 5.5 Hz, 1H), 11.93 (s, 1H).

^{13}C NMR (DMSO-d$_6$, 75 MHz): δ 58.14 (CH_3), 67.09 (CH_2), 70.07 (CH_2), 99.01 (CH), 106.71 (CH), 107.28 (C_{quat}), 112.78 (CH), 113.95 (C_{quat}), 123.38 (CH), 143.51 (C_{quat}), 145.09 (CH), 146.76 (CH), 150.99 (C_{quat}), 158.98 (C_{quat}), 159.64 (C_{quat}).

IR (neat): $\tilde{\nu}$ [cm^{-1}]: 3500 (w), 3304 (w), 3130 (w), 3092 (w), 2999 (w), 2938 (w), 2882 (w), 2824 (w), 2793 (w), 2735 (w), 2604 (w), 1595 (m), 1580 (m), 1547 (m), 1522 (m), 1508 (m), 1447 (m), 1429 (m), 1400 (w), 1356 (w), 1329 (w), 1306 (m), 1279 (m), 1261 (m), 1244 (m), 1198 (w), 1153 (m), 1125 (m), 1099 (m), 1084 (m), 1022 (m), 1009 (w), 989 (w), 897 (m), 854 (s), 799 (s), 777 (w), 746 (w), 733 (w), 708 (w), 667 (w), 650 (m), 635 (s), 612 (m).

MS (EI, *m/z* (%)): 285 (14), 284 ([M]$^+$, 64), 227 ([$C_{12}H_{11}N_4O$]$^+$, 16), 226 ([$C_{12}H_{10}N_4O$]$^+$, 100), 225 ([$C_{12}H_9N_4O$]$^+$, 31), 186 ([$C_{10}H_{10}N_4$]$^+$, 11).

ESI HRMS calcd. for [$C_{15}H_{17}N_4O_2$]$^+$: 285.1346; **Found:** 285.1348.

HPLC t_r: 0.8 min (no retention, >99 % purity).

4.7 4-(4-(Hexyloxy)-1*H*-pyrrolo[2,3-b]pyridin-3-yl)pyridin-2-amine (3g)

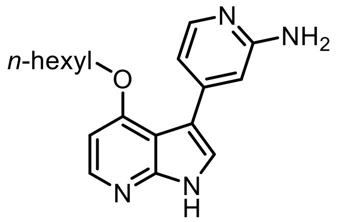

3g
[310.40]
$C_{18}H_{22}N_4O$

According to GP 3 product **3g** (191 mg, 0.67 mmol, 67%) was obtained as beige solid.

Mp 199 °C.

R_f (dichloromethane/methanol/aqueous ammonia (95:5:1)): 0.32.

^{1}H NMR (DMSO-d$_6$, 300 MHz): δ 0.74 – 0.88 (m, 3H), 1.22 – 1.28 (m, 4H), 1.32 – 1.39 (m, 2H), 1.59 – 1.83 (m, 2H), 4.11 (t, $^3J_{HH}$ = 6.4 Hz, 2H), 5.68 (s, 2H), 6.68 (d, $^3J_{HH}$ = 5.5 Hz, 1H), 6.72 (d, $^3J_{HH}$ = 1.5 Hz, 1H), 6.79 (dd, $^3J_{HH}$ = 5.3 Hz, $^4J_{HH}$ = 1.5 Hz, 1H), 7.52 (d, $^4J_{HH}$ = 1.7 Hz, 1H), 7.81 (d, $^3J_{HH}$ = 5.3 Hz, 1H), 8.11 (d, $^3J_{HH}$ = 5.4 Hz, 1H), 11.88 (s, 1H).

^{13}C NMR (DMSO-d$_6$, 75 MHz): δ 14.39 (CH_3), 22.53 (CH_2), 28.86 (CH_2), 31.52 (CH_2), 68.33 (CH_2), 99.40 (CH), 107.28 (C_{quat}), 107.73 (CH), 113.39 (CH), 114.52 (C_{quat}), 123.55 (CH), 144.14 (C_{quat}), 145.58 (CH), 147.09 (CH), 151.37 (C_{quat}), 159.78 (C_{quat}), 160.07 (C_{quat}).

IR (neat): $\tilde{\nu}$ [cm^{-1}]: 2839 (w), 2733 (w), 2673 (w), 2608 (w), 1626 (m), 1598 (s), 1580 (m), 1545 (m), 1520 (m), 1512 (m), 1462 (m), 1435 (m), 1414 (m), 1387 (w), 1346 (w), 1314 (s), 1294 (m), 1269 (m), 1153 (m), 1092 (m), 988 (m), 918 (m), 880 (m), 858 (m), 800 (s), 723 (m), 671 (m), 694 (w), 652 (m), 634 (s), 617 (m).

MS (EI, *m/z* (%)): 312 ([H^{13}CM]$^+$. 19), 311 ([HM]$^+$. 75), 310 ([M]$^+$, 53), 239 ([$C_{13}H_{11}N_4O$]$^+$, 20), 227 ([$C_{12}H_{14}N_4O$]$^+$, 22), 226 ([$C_{12}H_{13}N_4O$]$^+$, 100), 225 ([$C_{12}H_{12}N_4O$]$^+$, 35), 198 (10).

ESI HRMS calcd. for [$C_{18}H_{23}N_4O$]$^+$: 311.1866; **Found:** 311.1867.

HPLC t_r: 2.2 min (>99 % purity).

4.8 4-(4-(Octyloxy)-1*H*-pyrrolo[2,3-b]pyridin-3-yl)pyridin-2-amine (3h)

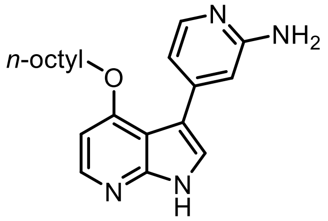

3h
[338.46]
$C_{20}H_{26}N_4O$

According to GP 3 product **3h** (191 mg, 0.56 mmol, 56%) was obtained as beige solid.

Mp 175 °C.

R_f (dichloromethane/methanol/aqueous ammonia (95:5:1)): 0.26.

^{1}H NMR (DMSO-d$_6$, 600 MHz): δ 0.85 (t, $^3J_{HH}$ = 7.0 Hz, 3H), 1.16 – 1.30 (m, 8H), 1.30 – 1.38 (m, 2H), 1.77 (p, $^3J_{HH}$ = 6.6 Hz, 2H), 4.11 (t, $^3J_{HH}$ = 6.4 Hz, 2H), 5.69 (s, 2H), 6.69 (d, $^3J_{HH}$ = 5.5 Hz, 2H), 6.72 (d, $^3J_{HH}$ = 1.5 Hz, 1H), 6.79 (dd, $^3J_{HH}$ = 5.3 Hz, $^4J_{HH}$ = 1.6 Hz, 1H), 7.52 (d, $^4J_{HH}$ = 2.3 Hz, 1H), 7.81 (d, $^3J_{HH}$ = 5.3 Hz, 1H), 8.11 (d, $^3J_{HH}$ = 5.5 Hz, 1H), 11.49 – 12.10 (m, 1H).

^{13}C NMR (DMSO-d$_6$, 150 MHz): δ 14.44 (CH_3), 22.56 (CH_2), 26.09 (CH_2), 28.89 (CH_2), 29.14 (CH_2), 29.23 (CH_2), 31.70 (CH_2), 68.32 (CH_2), 99.41 (CH), 107.28 (C_{quat}), 107.72 (CH), 113.38 (CH), 114.49 (C_{quat}), 123.60 (CH), 144.18 (C_{quat}), 145.58 (CH), 146.98 (CH), 151.38 (C_{quat}), 159.77 (C_{quat}), 160.01 (C_{quat}).

IR (neat): $\tilde{\nu}$ [cm^{-1}]: 3476 (w), 3304 (w), 3150 (w), 2920 (m), 2849 (w), 2723 (w), 1643 (w), 1609 (s), 1585 (s), 1539 (m), 1522 (m), 1466 (m), 1437 (m), 1410 (m), 1391 (m), 1317 (s), 1294 (m), 1265 (s), 1169 (w), 1150 (w), 1092 (s), 995 (m), 962 (m), 880 (m), 847 (s), 810 (s), 793 (s), 762 (m), 731 (m), 694 (w), 671 (m), 646 (m), 635 (s).

MS (EI, *m/z* (%)): 339 ([HM]$^+$, 11), 338 ([M]$^+$, 38), 227 ([$C_{12}H_{14}N_4O$]$^+$, 18), 226 ([$C_{12}H_{13}N_4O$]$^+$, 100), 225 ([$C_{12}H_{12}N_4O$]$^+$, 32), 198 (10), 186 (15).

ESI HRMS calcd. for [$C_{20}H_{27}N_4O$]$^+$: 339.2179; **Found:** 339.2183.

HPLC t_r: 2.7 min (>99 % purity).

4.9 4-(4-(Dodecyloxy)-1*H*-pyrrolo[2,3-b]pyridin-3-yl)pyridin-2-amine (3i)

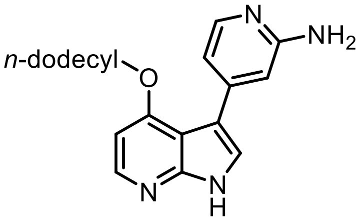

3i
[394.56]
$C_{24}H_{34}N_4O$

According to GP 3 product **3i** (278 mg, 0.70 mmol, 83%) was obtained as beige solid.

Mp 150 °C.

R_f (dichloromethane/methanol/aqueous ammonia (95:5:1)): 0.21.

^{1}H NMR (DMSO-d$_6$, 600 MHz): δ 0.84 (t, $^3J_{HH}$ = 6.9 Hz, 3H), 1.14 – 1.30 (m, 16H), 1.31 – 1.36 (m, 2H), 1.76 (p, $^3J_{HH}$ = 6.6 Hz, 2H), 4.10 (t, $^3J_{HH}$ = 6.4 Hz, 2H), 5.67 (s, 2H), 6.68 (d, $^3J_{HH}$ = 5.5 Hz, 1H), 6.73 (d, $^4J_{HH}$ = 1.5 Hz, 1H), 6.79 (dd, $^3J_{HH}$ = 5.3, $^4J_{HH}$ = 1.5 Hz, 1H), 7.52 (d, $^4J_{HH}$ = 1.9 Hz, 1H), 7.81 (d, $^3J_{HH}$ = 5.3 Hz, 1H), 8.11 (d, $^3J_{HH}$ = 5.4 Hz, 1H), 11.88 (s, 1H).

^{13}C NMR (DMSO-d$_6$, 150 MHz): δ 14.42 (CH_3), 22.57 (CH_2), 26.07 (CH_2), 28.88 (CH_2), 29.20 (CH_2), 29.26 (CH_2), 29.47 (CH_2), 29.48 (CH_2), 29.50 (CH_2), 29.53 (CH_2), 31.78 (CH_2), 68.29 (CH_2), 99.39 (CH), 107.28 (C_{quat}), 107.71 (CH), 113.38 (CH), 114.52 (C_{quat}), 123.55 (CH), 144.13 (C_{quat}), 145.56 (CH), 147.07 (CH), 151.39 (C_{quat}), 159.77 (C_{quat}), 160.07 (C_{quat}).

IR (neat): $\tilde{\nu}$ [cm^{-1}]: 3140 (w), 2920 (m), 2847 (m), 1634 (m), 1585 (s), 1545 (m), 1514 (m), 1464 (m), 1433 (m), 1341 (w), 1323 (m), 1308 (s), 1290 (m), 1279 (m), 1265 (m), 1155 (m), 1090 (m), 868 (m), 860 (m), 826 (m), 793 (s), 770 (m), 721 (m), 710 (m), 667 (m), 635 (s), 615 (m).

MS (EI, *m/z* (%)): 395 ([HM]$^+$, 11), 394 ([M]$^+$, 27), 365 (12), 337 (14), 323 (11), 309 (11), 295 (14), 281 (12), 239 (33), 227 ([$C_{12}H_{14}N_4O$]$^+$, 23), 226 ([$C_{12}H_{13}N_4O$]$^+$, 100), 225 ([$C_{12}H_{12}N_4O$]$^+$, 29), 186 (13), 57 (11), 55 (16).

Analyse calcd. for $C_{24}H_{34}N_4O$ [394.56]: C 73.06, H 8.69, N 14.20, S 6.43; **Found:** C 73.25, H 8.51 N 14.04.

5. NMR Spectra

4-Ethoxy-1*H*-pyrrolo[2,3-b]pyridine (1c)

^{1}H NMR (DMSO-d_6, 300 MHz, 298 K)

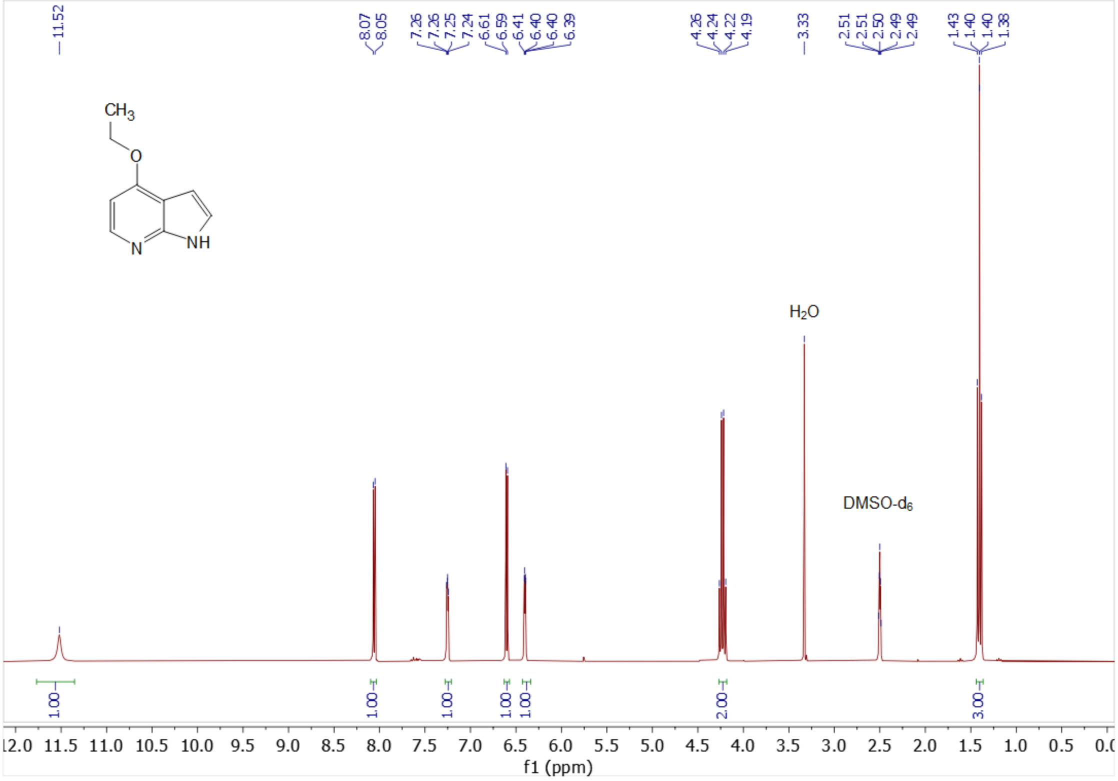

^{13}C NMR (DMSO-d_6, 75 MHz, 298 K)

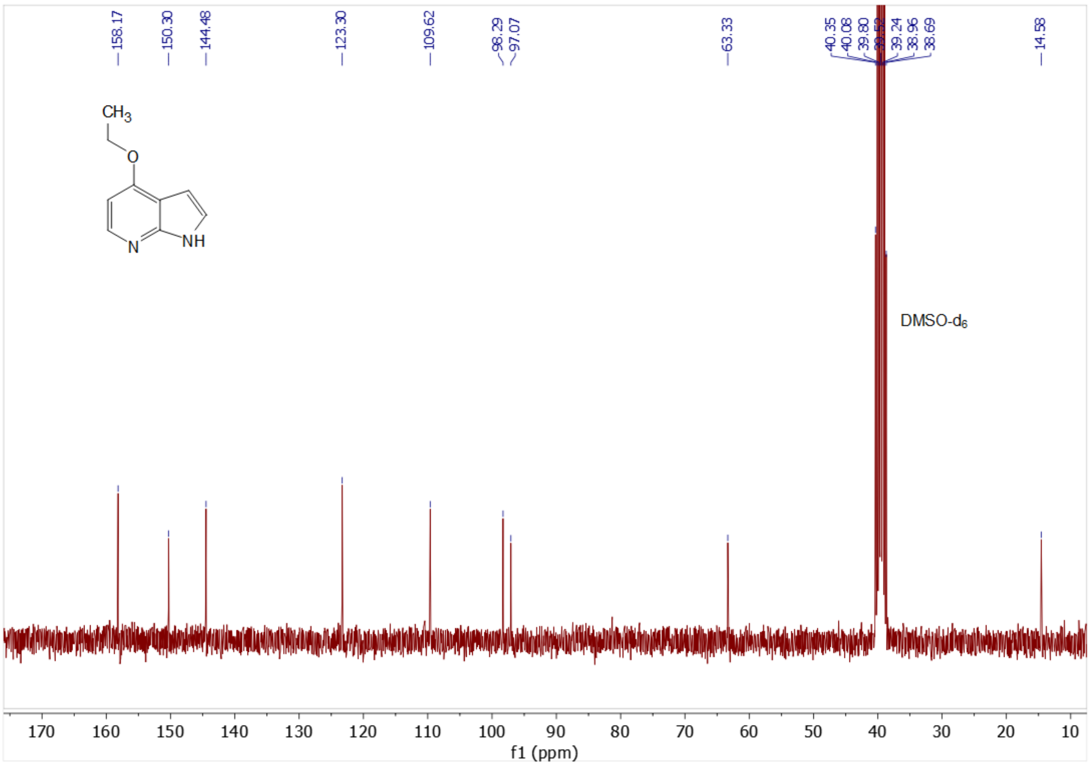

4-Propoxy-1*H*-pyrrolo[2,3-b]pyridine (1d)

^{1}H NMR (DMSO-d$_6$, 300 MHz, 298 K)

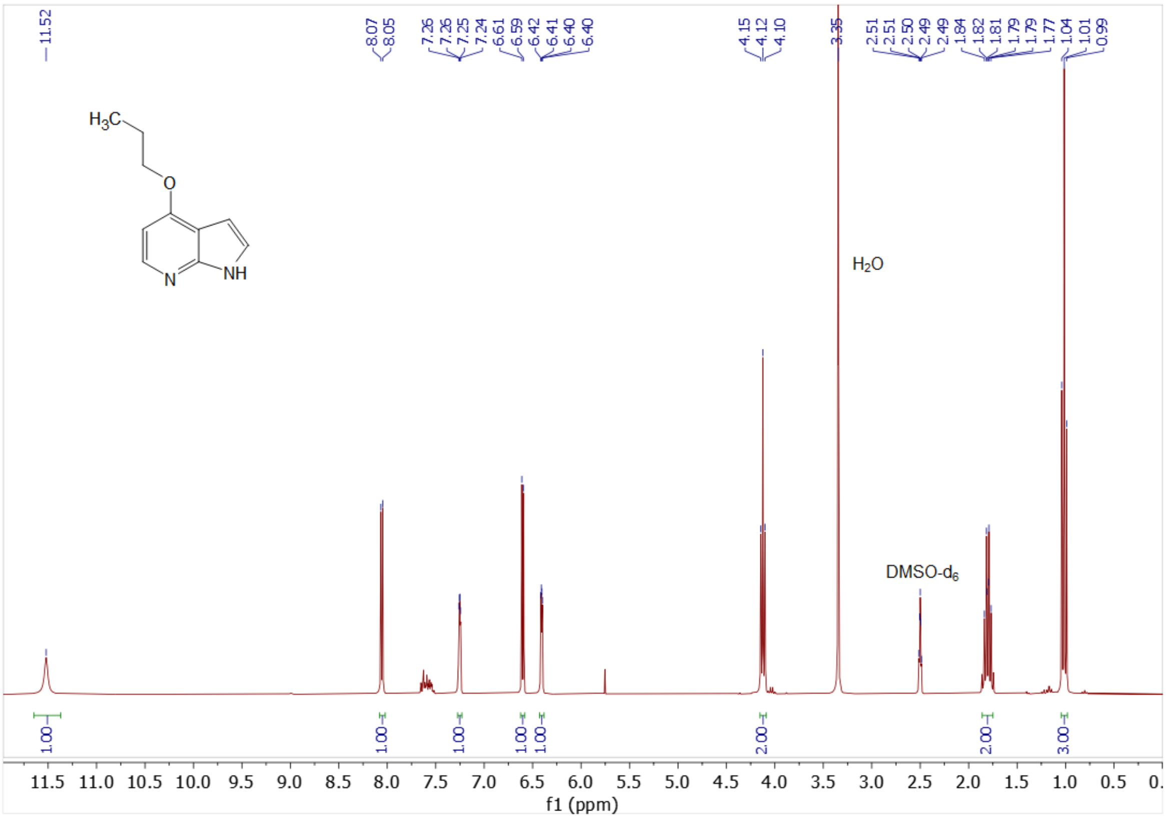

^{13}C NMR (DMSO-d$_6$, 75 MHz, 298 K)

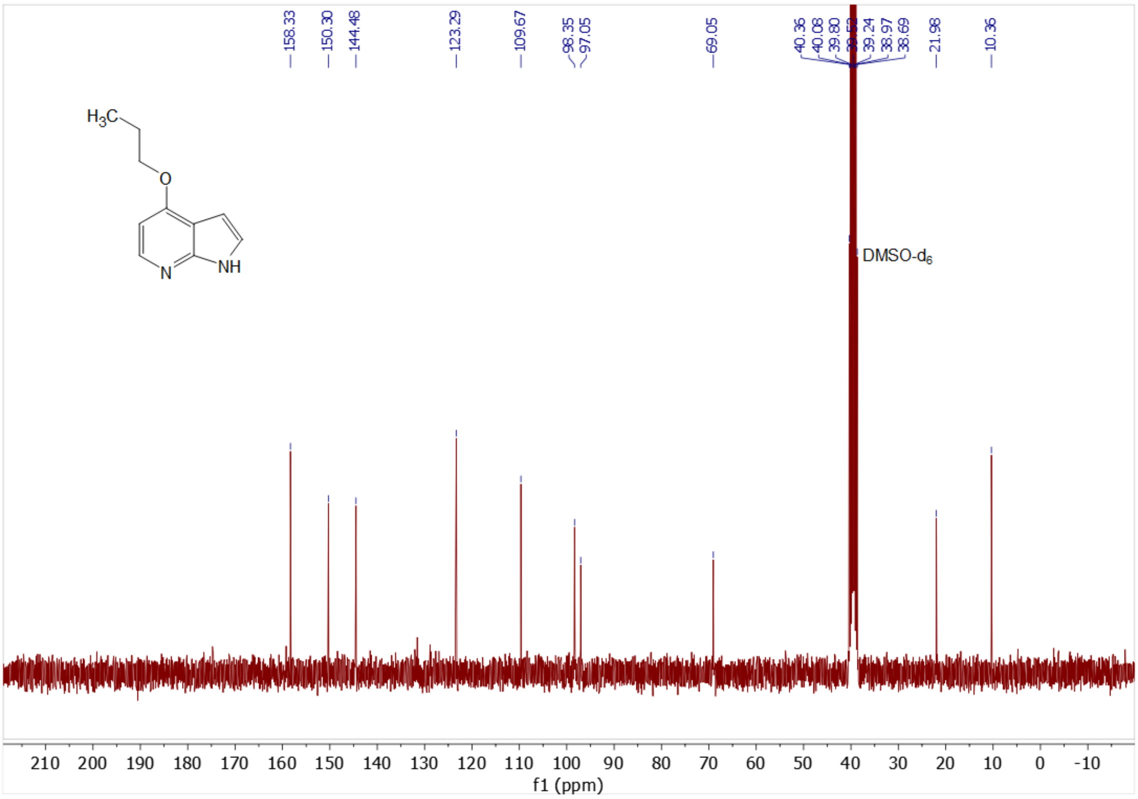

4-Butoxy-1*H*-pyrrolo[2,3-b]pyridine (1e)

^{1}H NMR (DMSO-d$_6$, 300 MHz, 298 K)

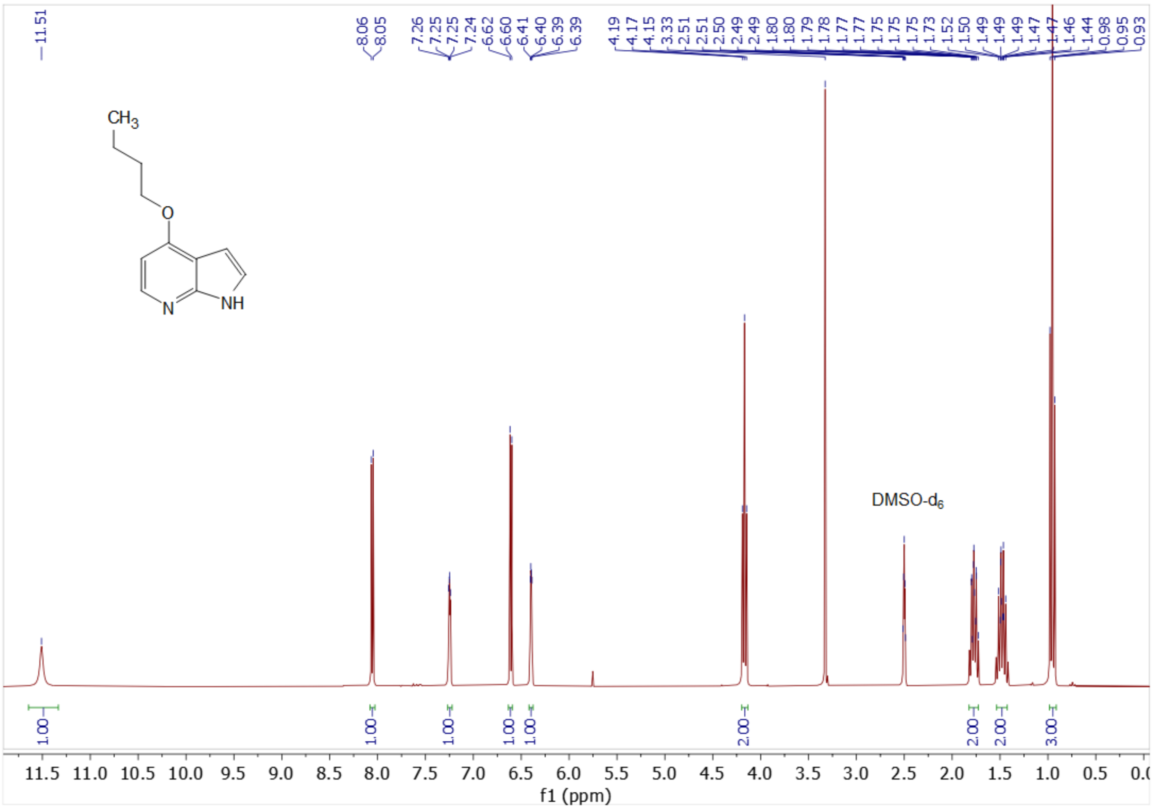

^{13}C NMR (DMSO-d$_6$, 75 MHz, 298 K)

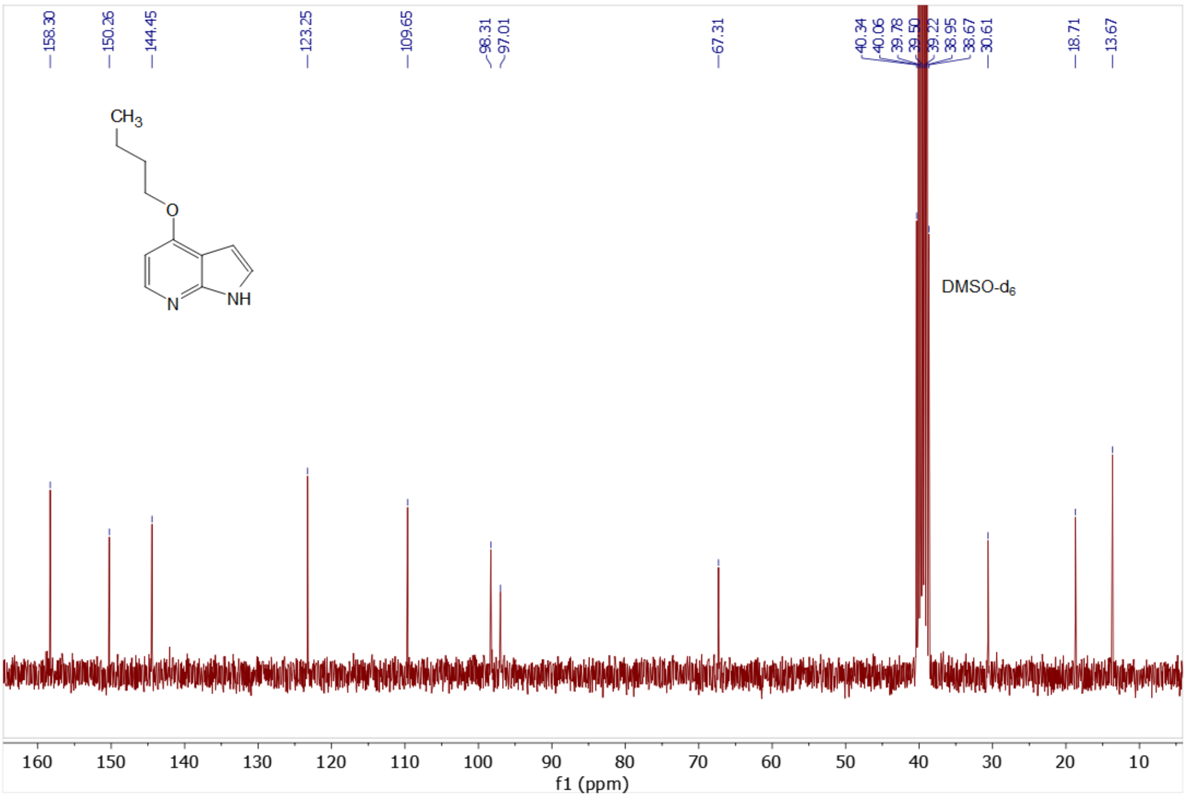

4-(2-methoxyethoxy)-1H-pyrrolo[2,3-b]pyridine (1f)

^{1}H NMR (DMSO-d_6, 300 MHz, 298 K)

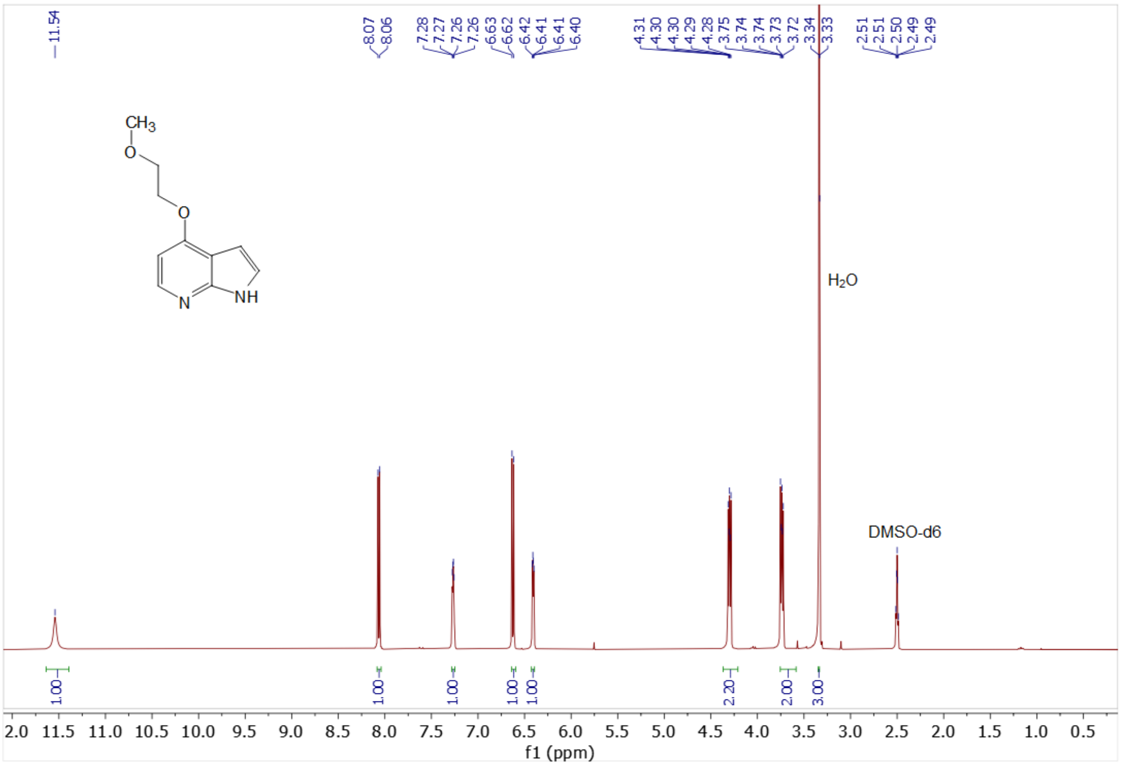

^{13}C NMR (DMSO-d_6, 75 MHz, 298 K)

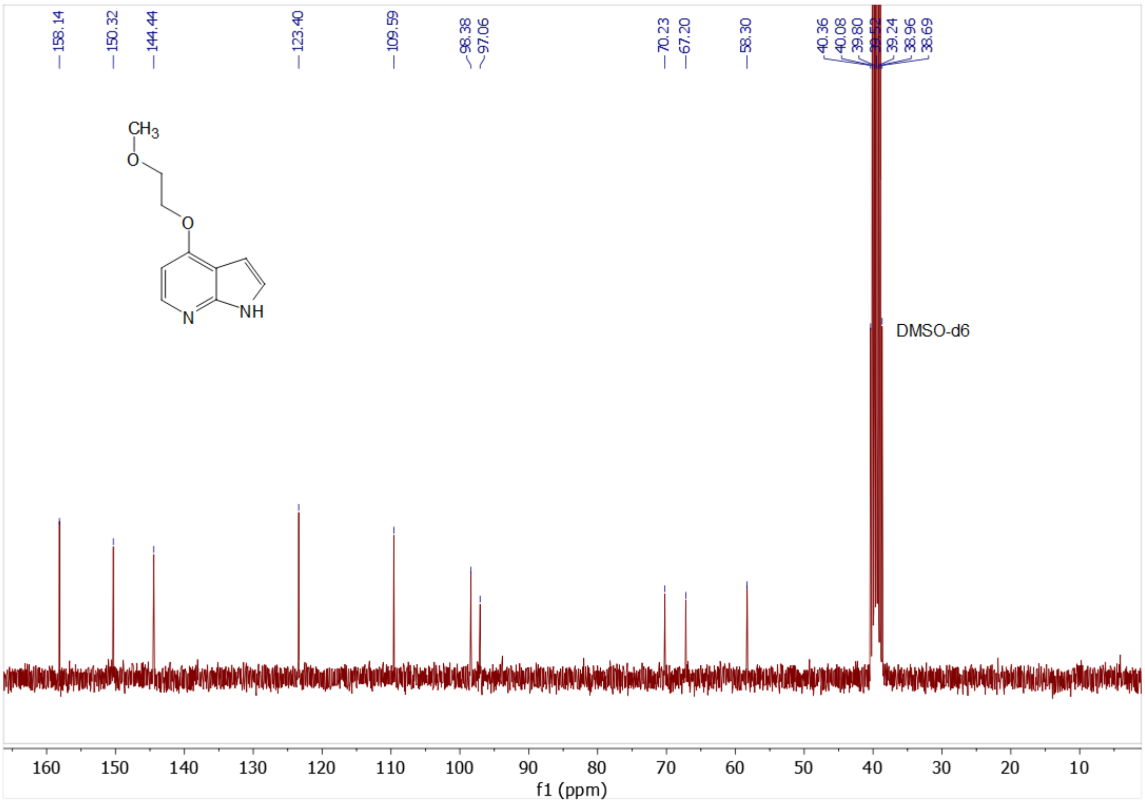

4-(Hexyloxy)-1*H*-pyrrolo[2,3-b]pyridine (1g)

^{1}H NMR (DMSO-d$_6$, 600 MHz, 298 K)

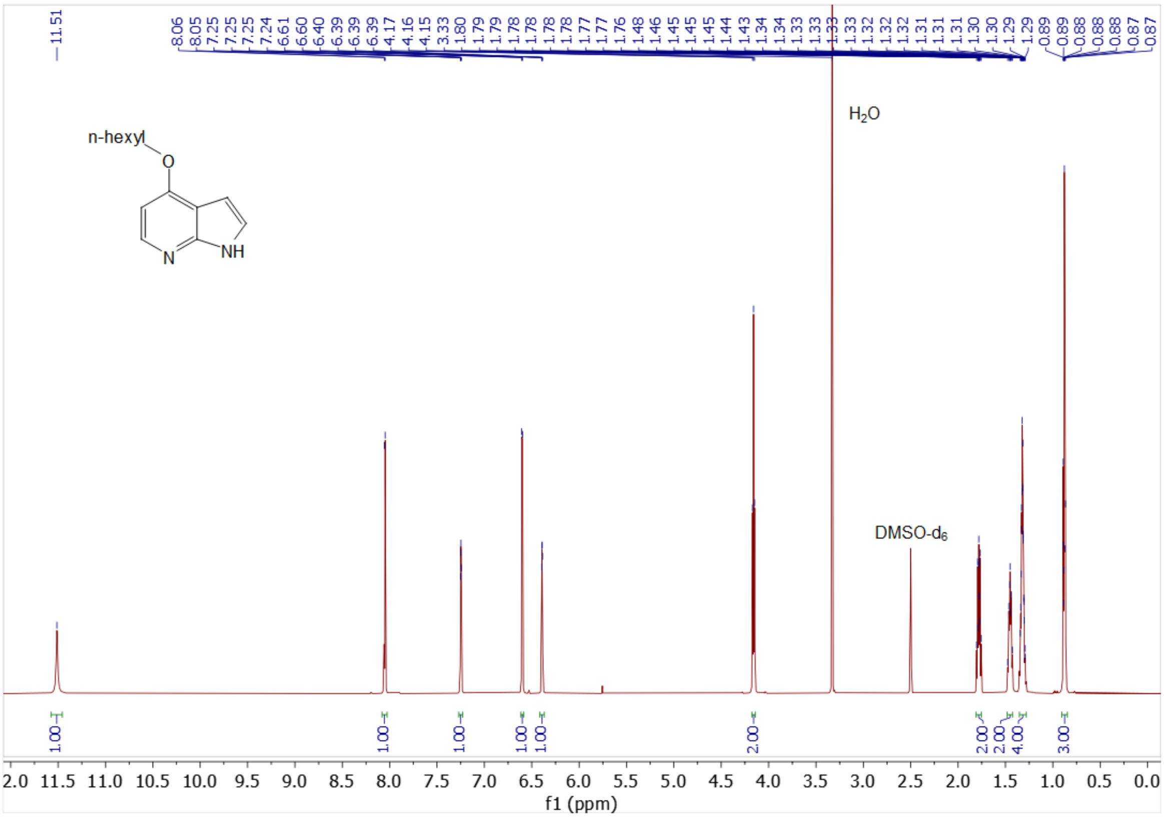

^{13}C NMR (DMSO-d$_6$, 150 MHz, 298 K)

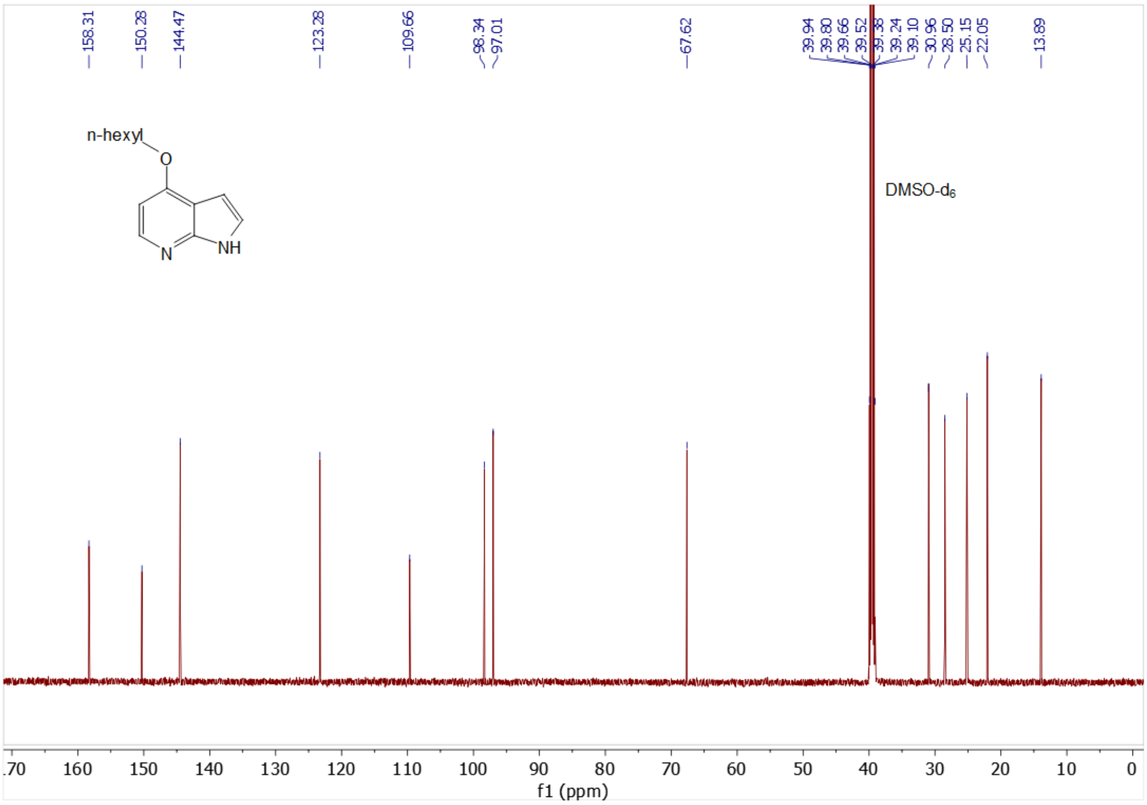

4-(Octyloxy)-1*H*-pyrrolo[2,3-b]pyridine (1h)

^{1}H NMR (DMSO-d$_6$, 300 MHz, 298 K)

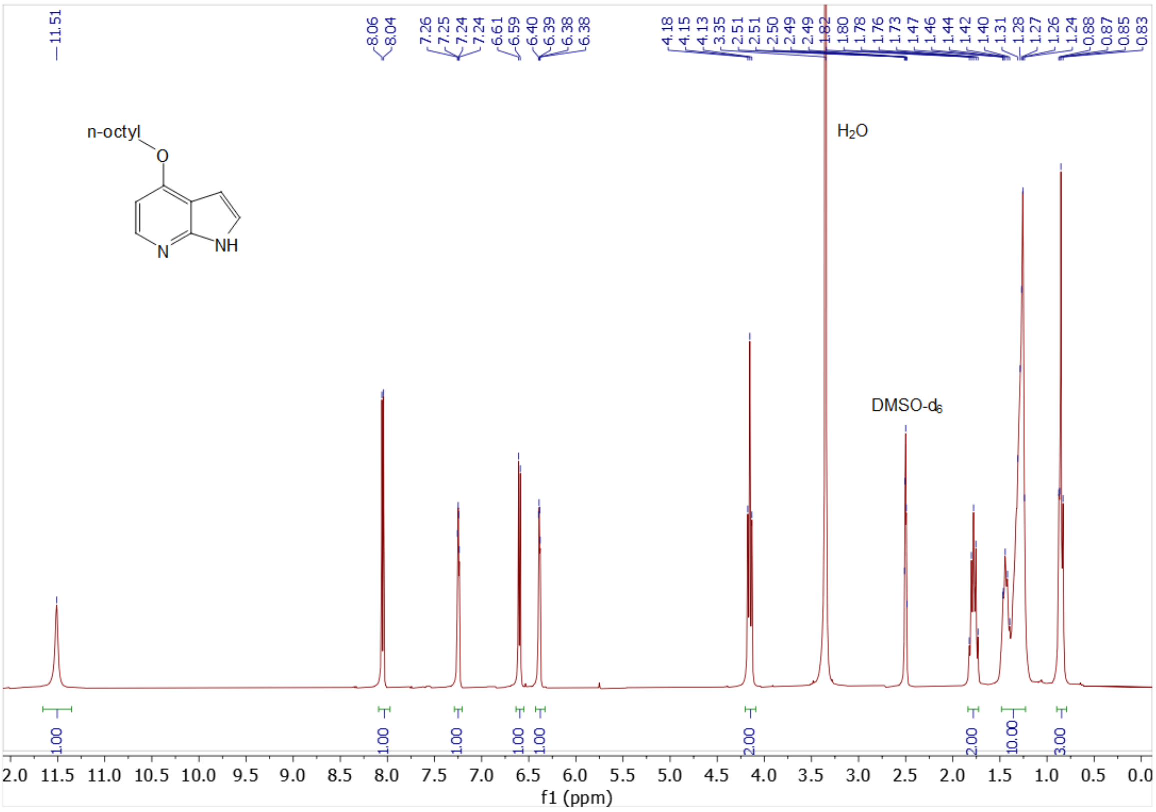

^{13}C NMR (DMSO-d$_6$, 75 MHz, 298 K)

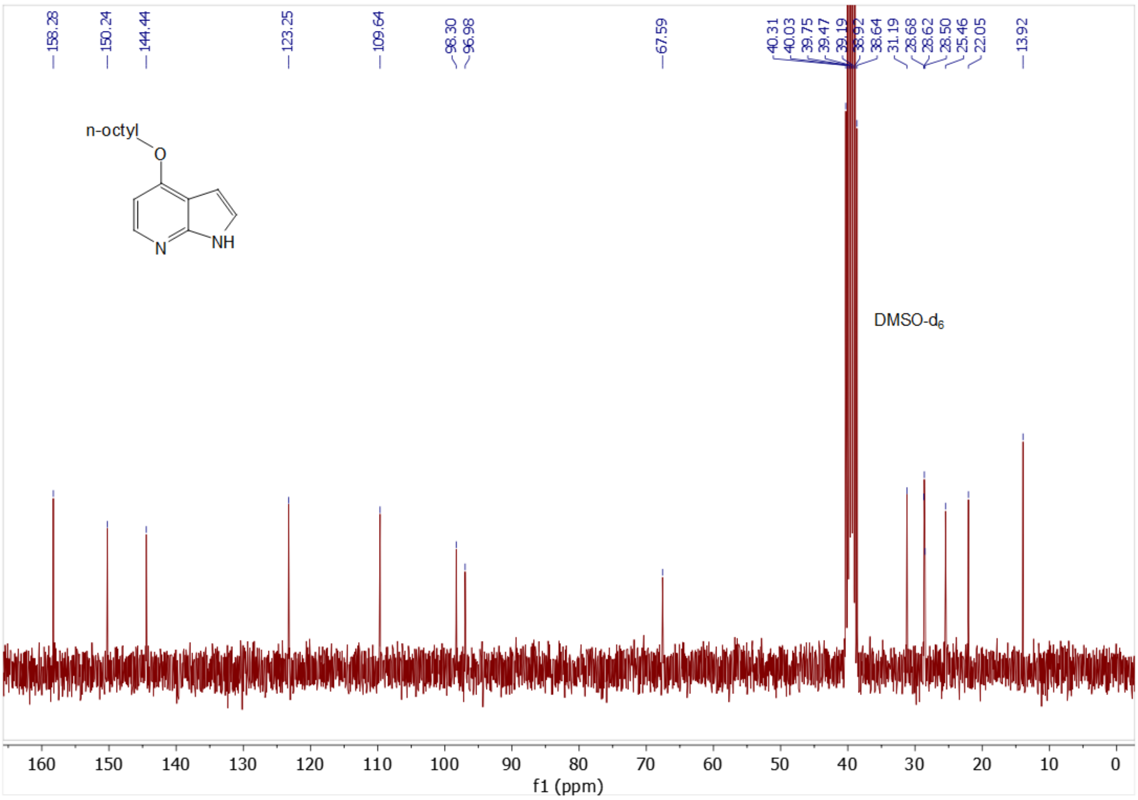

4-(Dodecyloxy)-1*H*-pyrrolo[2,3-b]pyridine (1i)

^{1}H NMR (DMSO-d_6, 600 MHz, 298 K)

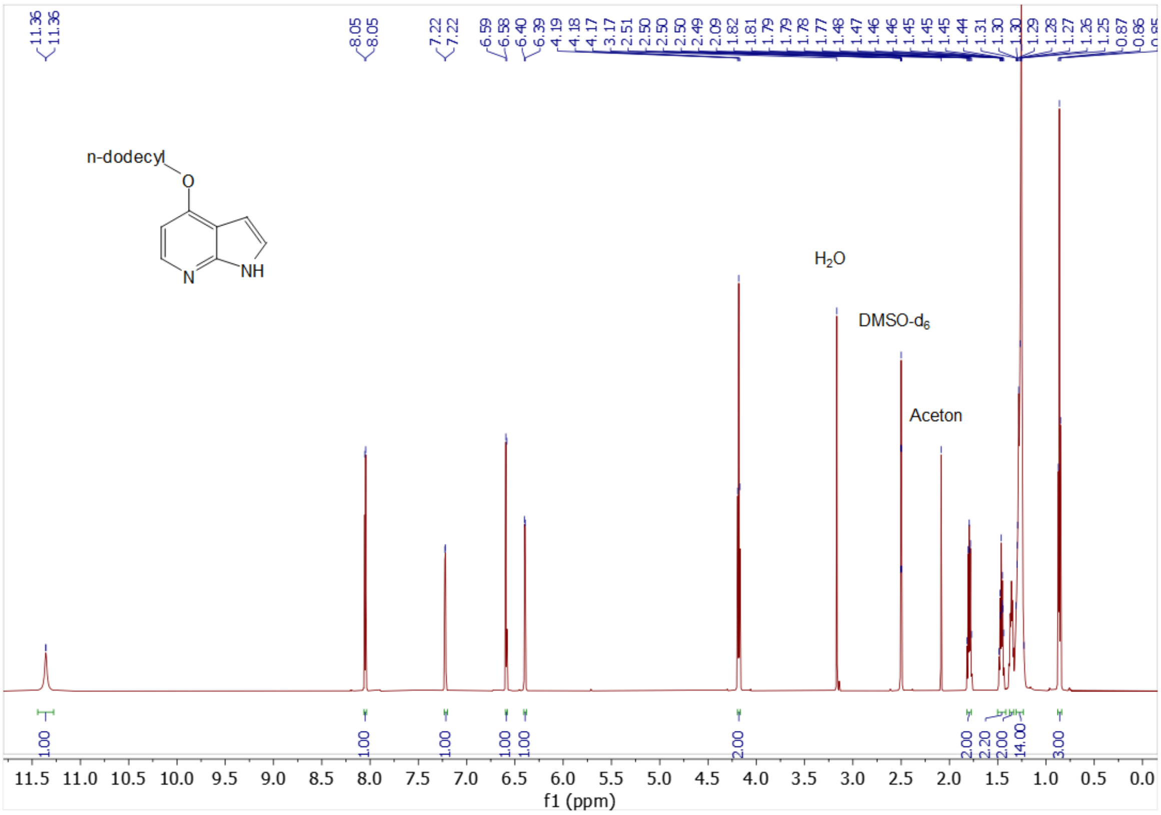

^{13}C NMR (DMSO-d_6, 150 MHz, 298 K)

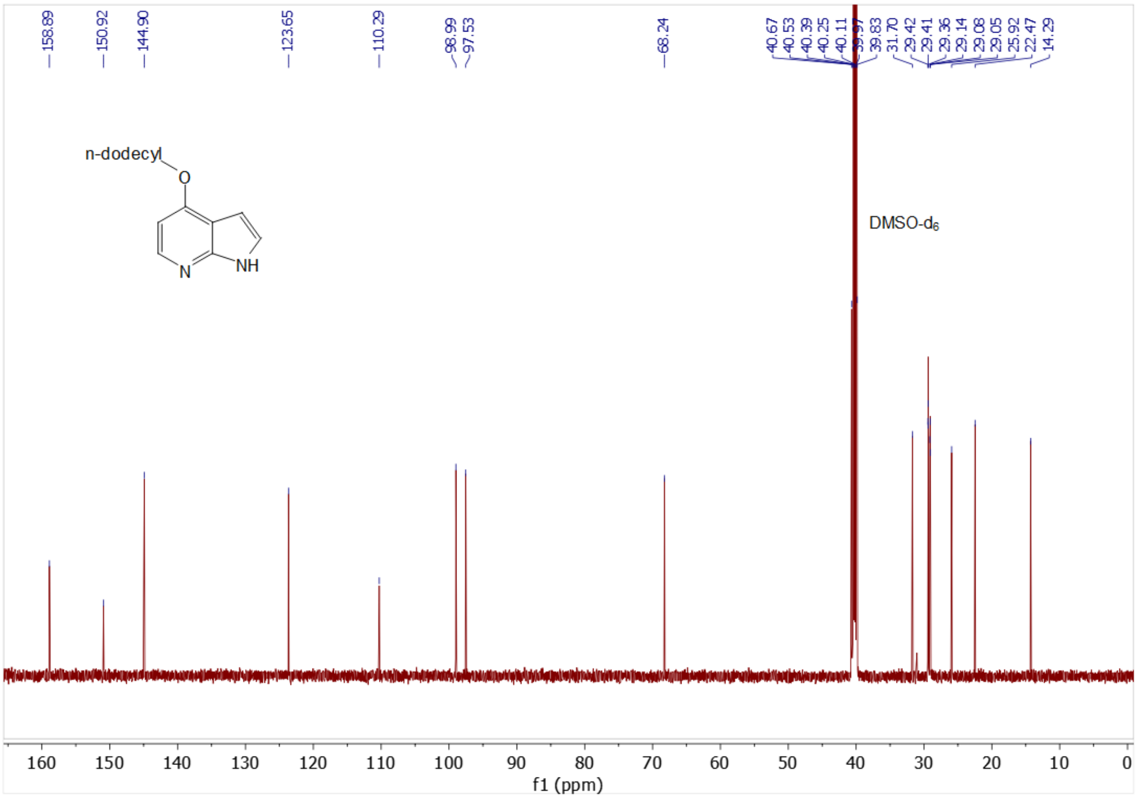

4-Chloro-3-iodo-1-tosyl-1H-pyrrolo[2,3-b]pyridine (2a)

^{1}H NMR (DMSO-d$_6$, 300 MHz, 298 K)

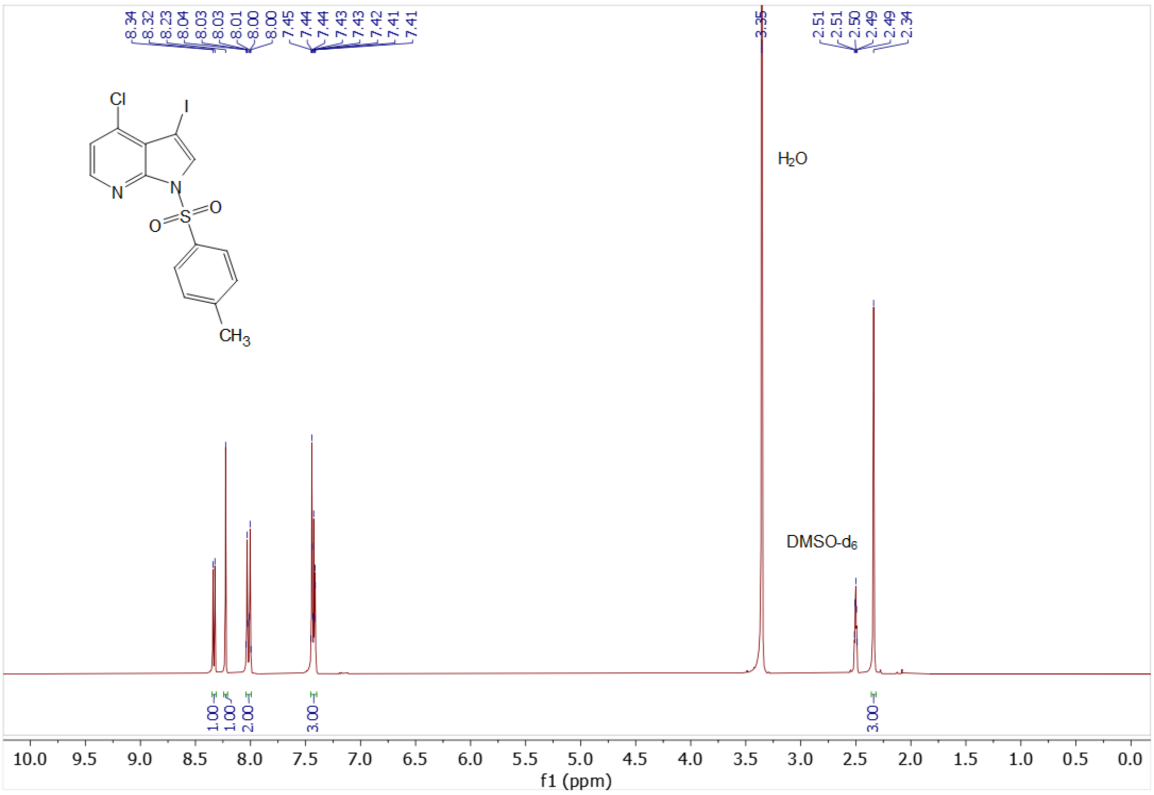

^{13}C NMR (DMSO-d$_6$, 75 MHz, 298 K)

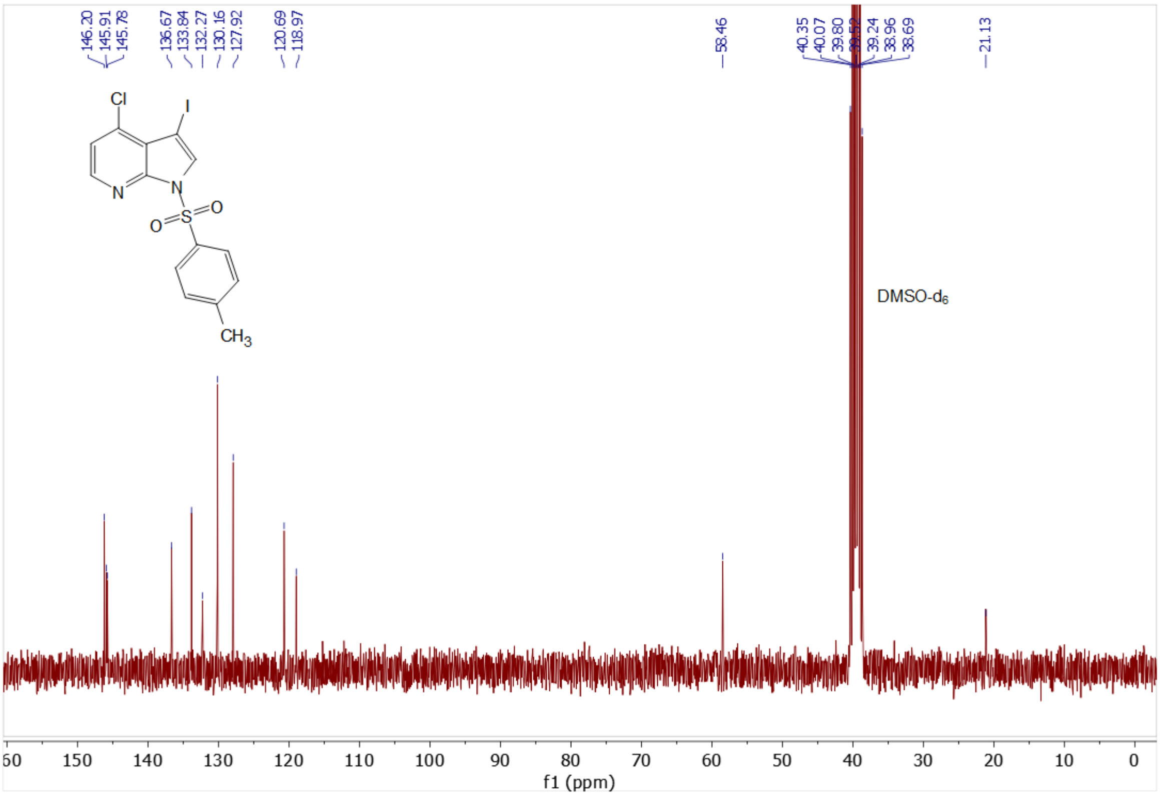

3-Iodo-4-methoxy-1-tosyl-1*H*-pyrrolo[2,3-b]pyridine (2b)

^{1}H NMR (DMSO-d$_6$, 300 MHz, 298 K)

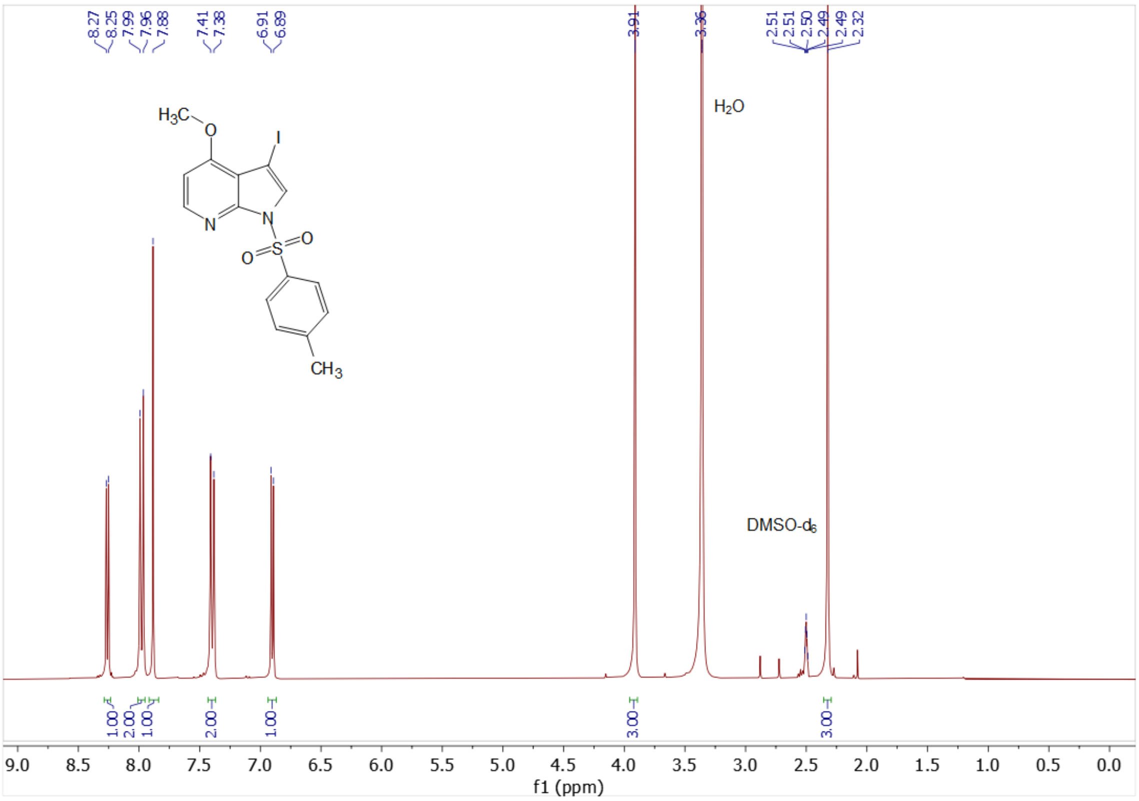

^{13}C NMR (DMSO-d$_6$, 75 MHz, 298 K)

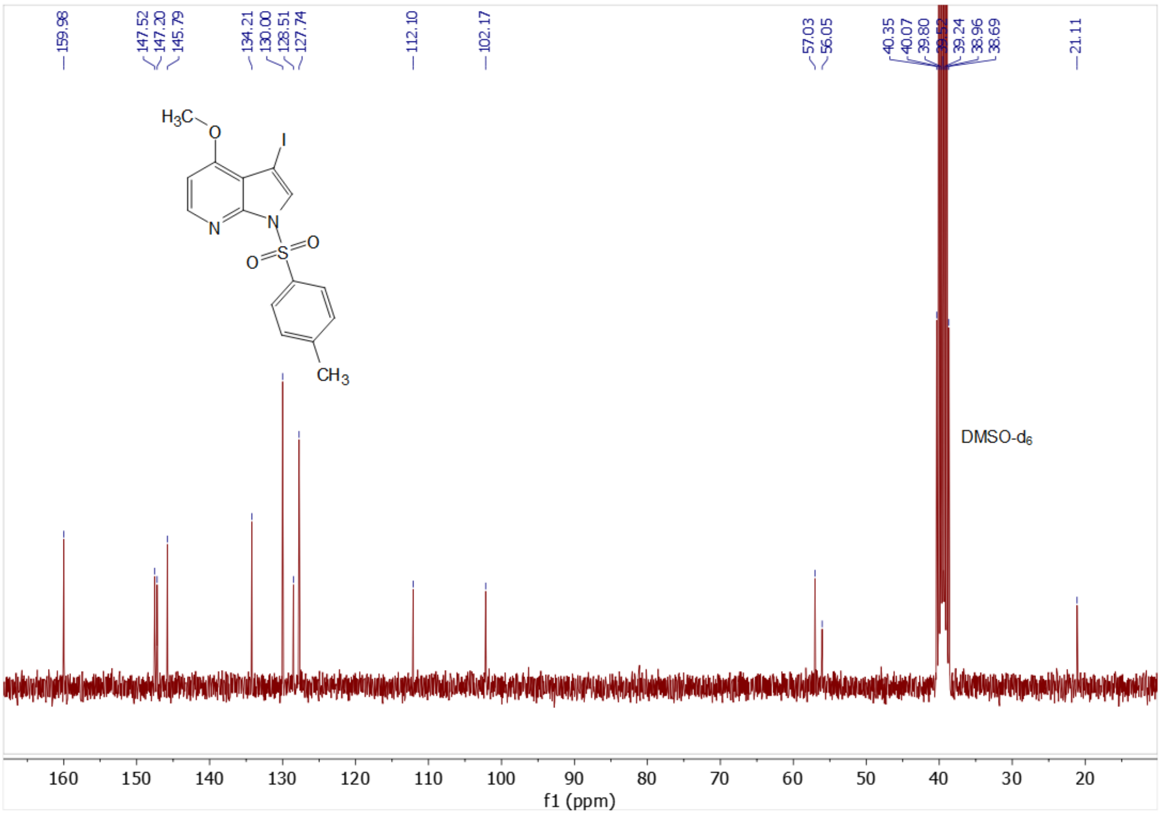

4-Ethoxy-3-iodo-1-tosyl-1*H*-pyrrolo[2,3-b]pyridine (2c)

^{1}H NMR (DMSO-d$_6$, 300 MHz, 298 K)

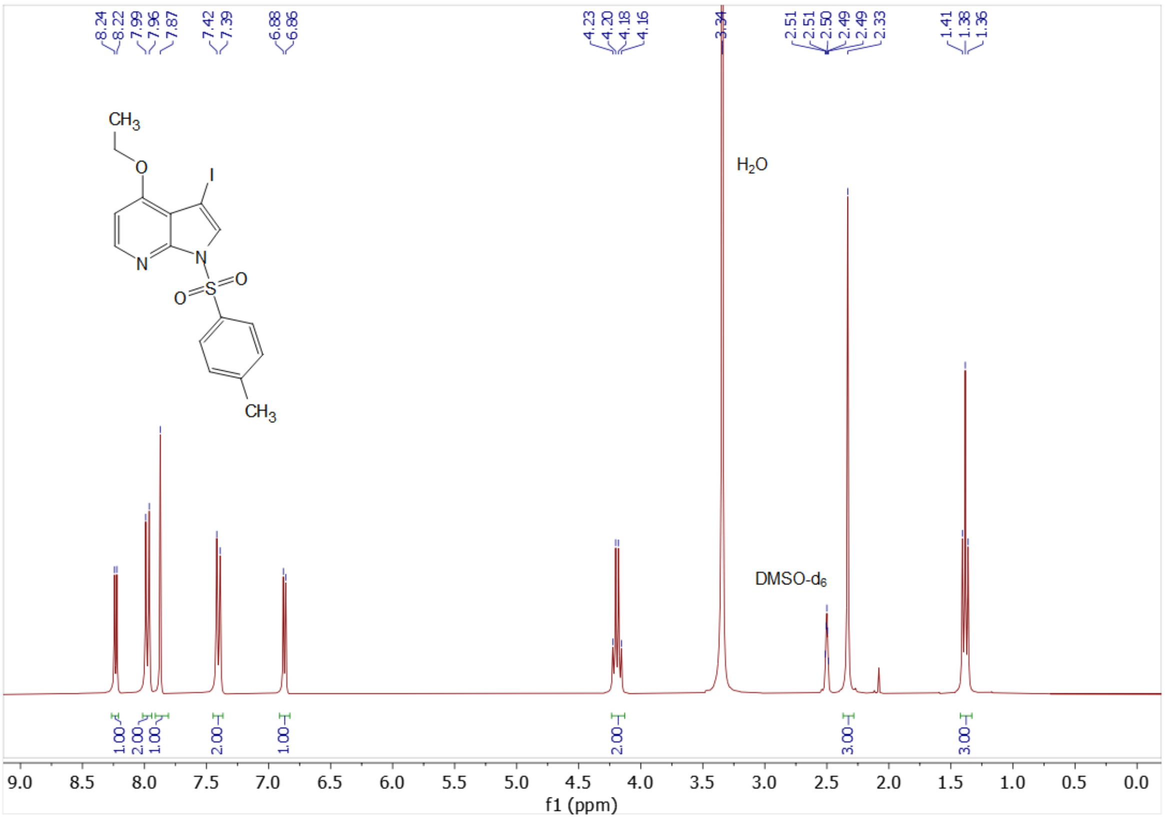

^{13}C NMR (DMSO-d$_6$, 75 MHz, 298 K)

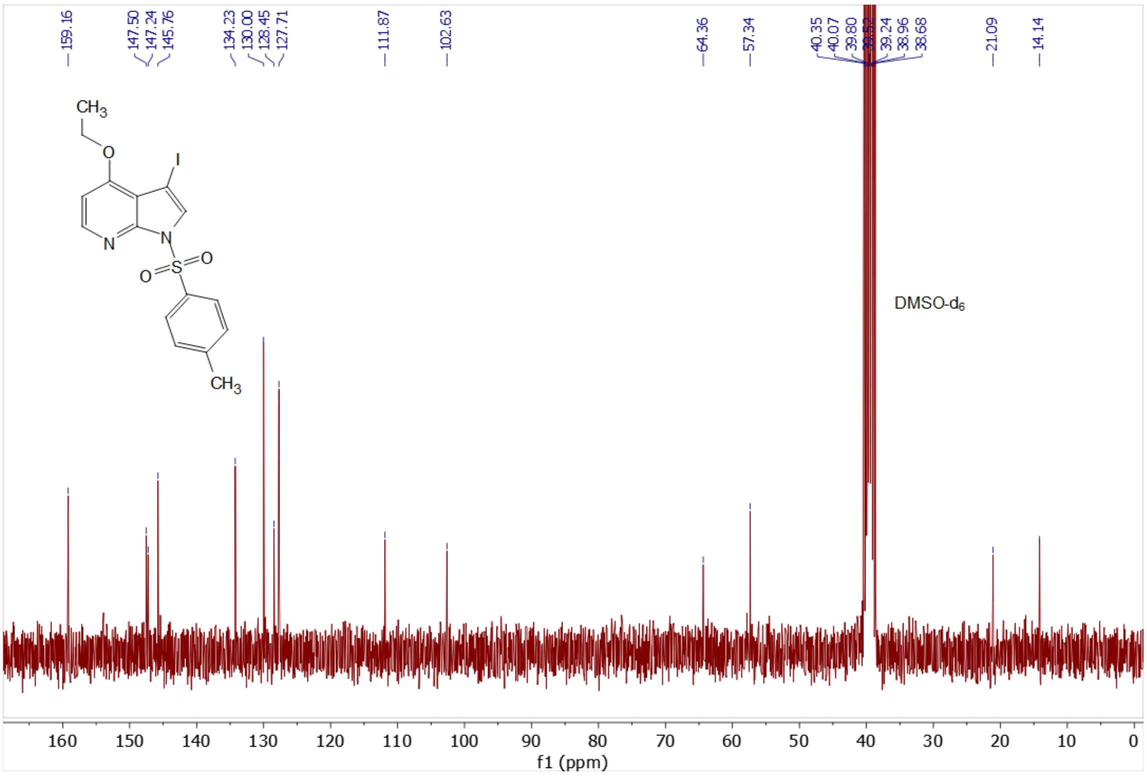

3-Iodo-4-propoxy-1-tosyl-1*H*-pyrrolo[2,3-b]pyridine (2d)

^{1}H NMR (DMSO-d$_6$, 300 MHz, 298 K)

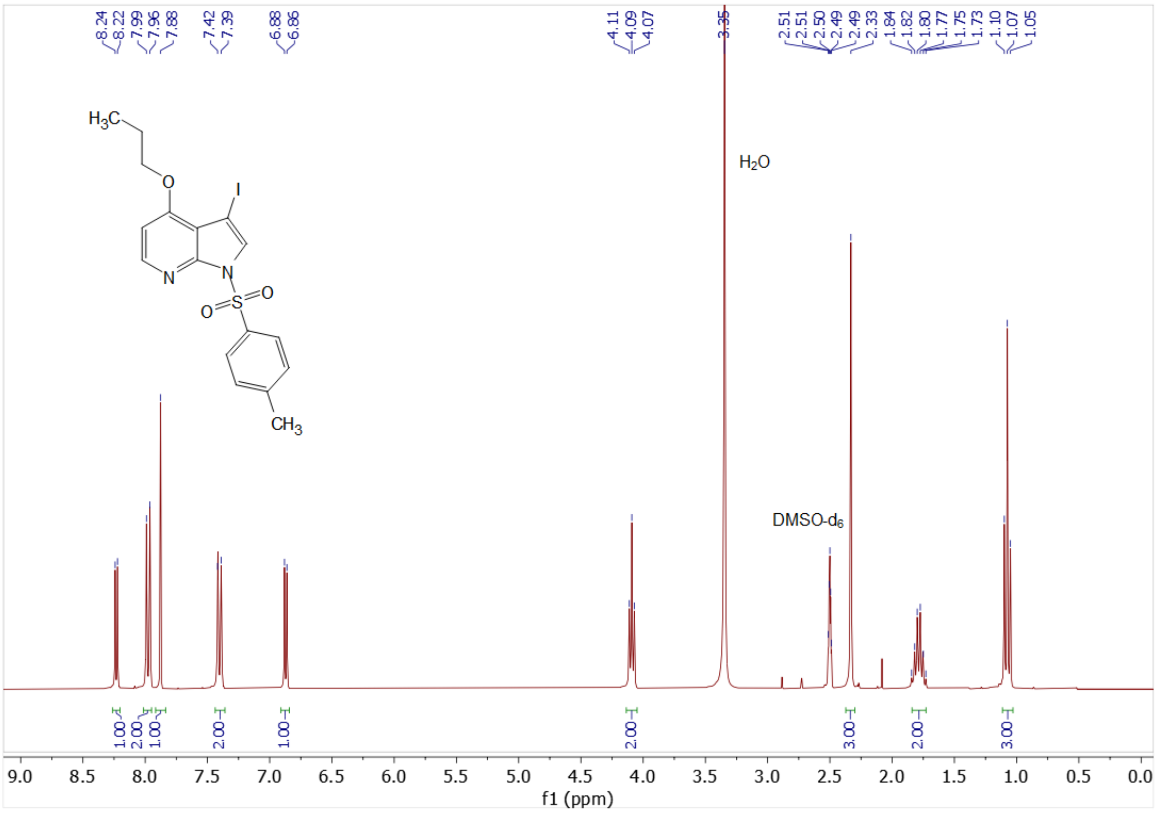

^{13}C NMR (DMSO-d$_6$, 75 MHz, 298 K)

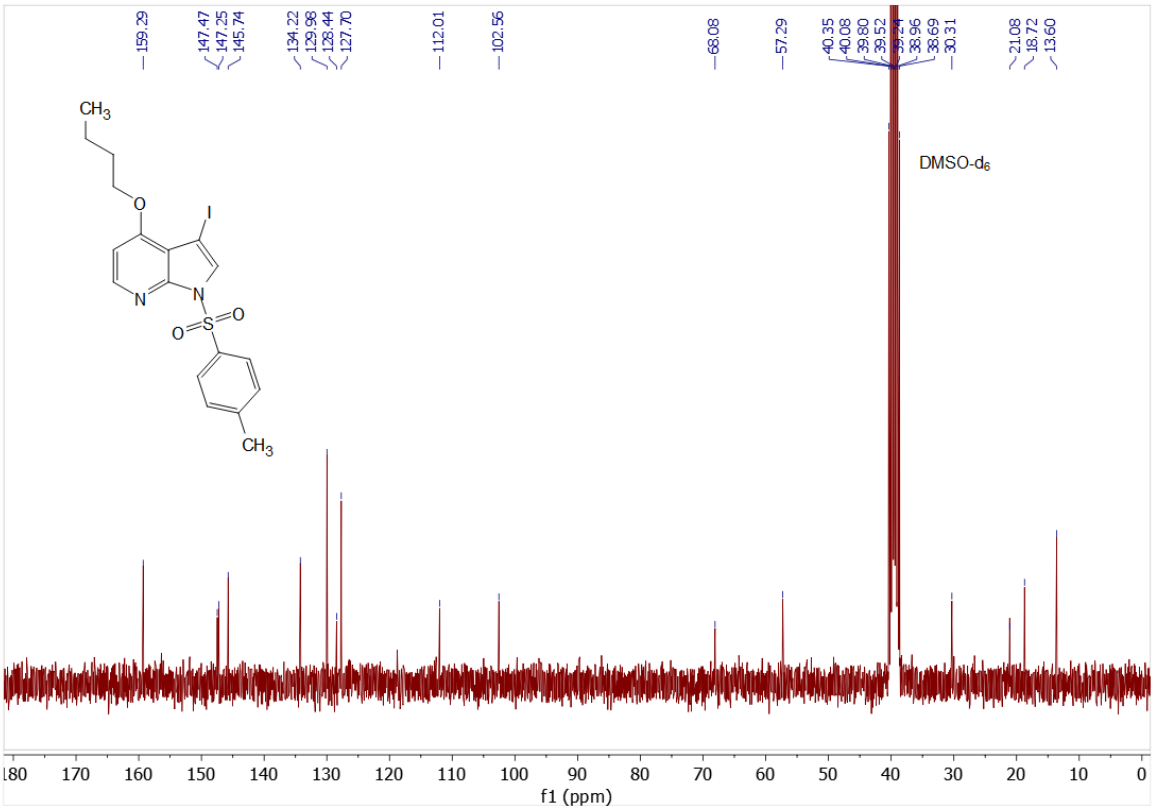

4-Butoxy-3-iodo-1-tosyl-1*H*-pyrrolo[2,3-b]pyridine (2e)

^{1}H NMR (DMSO-d$_6$, 300 MHz, 298 K)

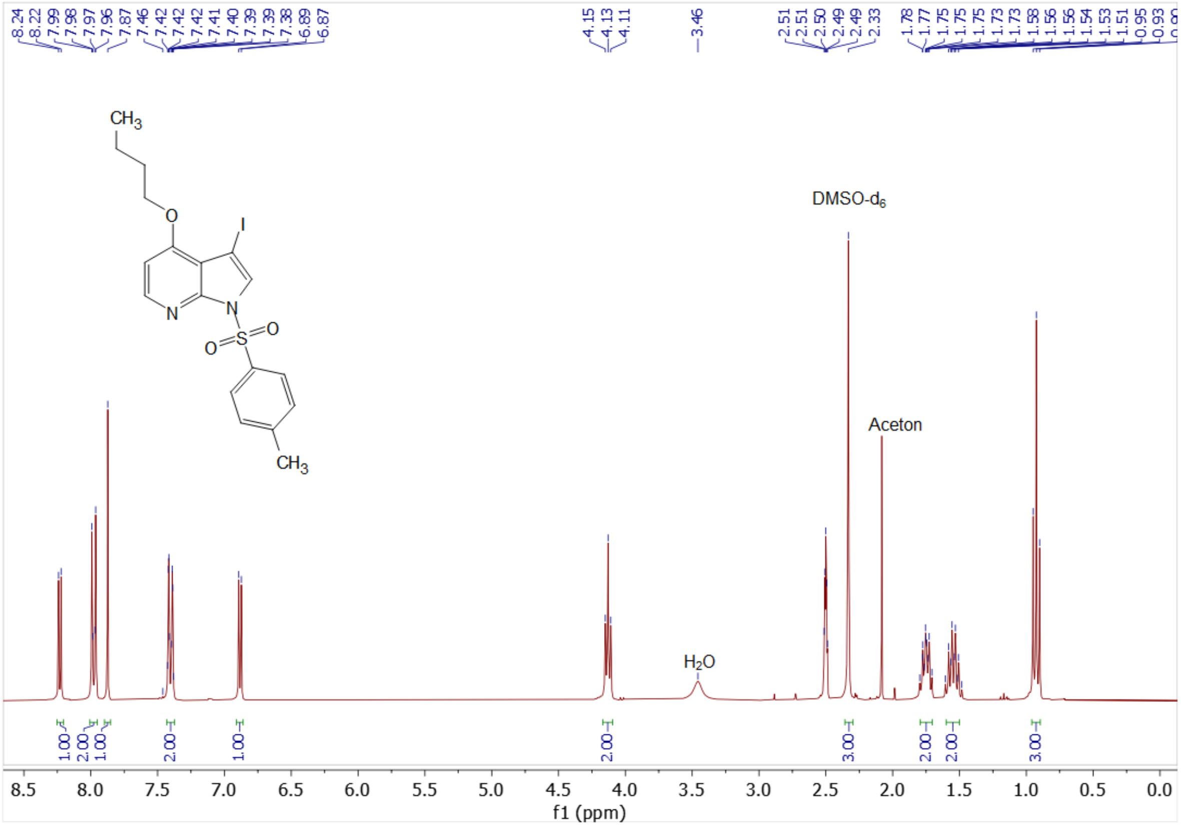

^{13}C NMR (DMSO-d$_6$, 75 MHz, 298 K)

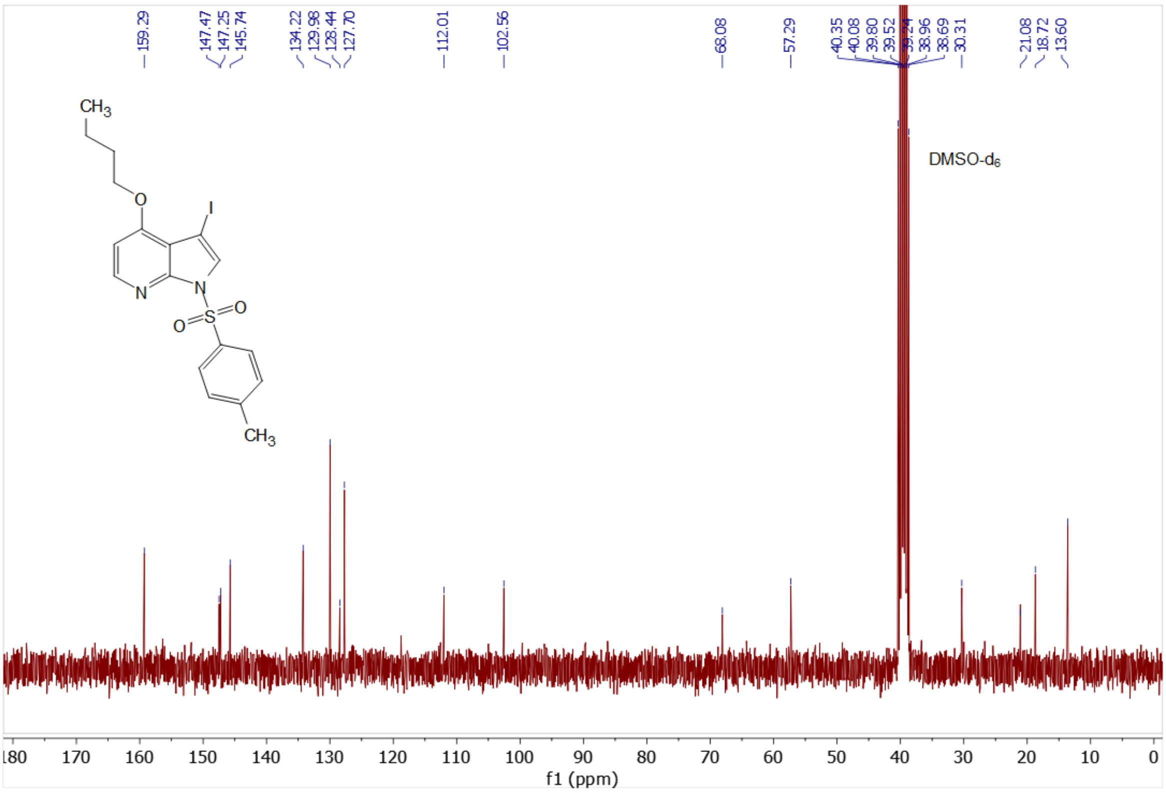

3-Iodo-4-(2-methoxyethoxy)-1-tosyl-1*H*-pyrrolo[2,3-b]pyridine (2f)

^{1}H NMR (DMSO-d$_6$, 300 MHz, 298 K)

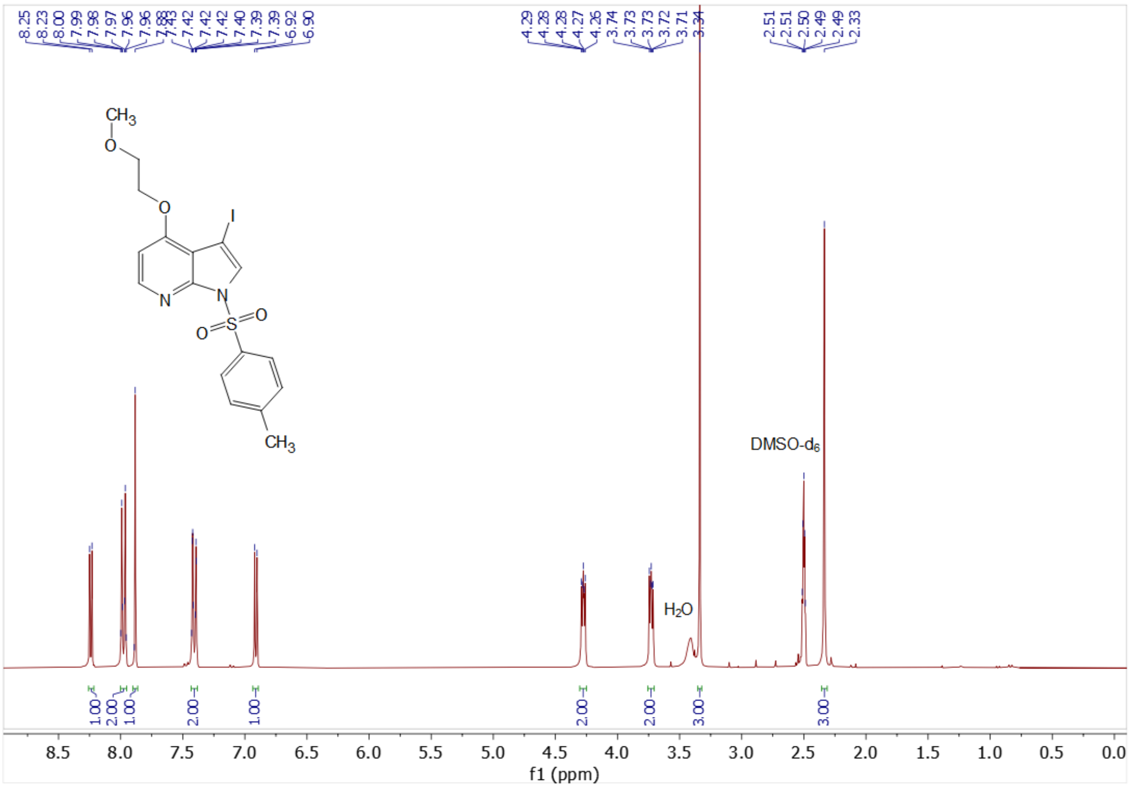

^{13}C NMR (DMSO-d$_6$, 75 MHz, 298 K)

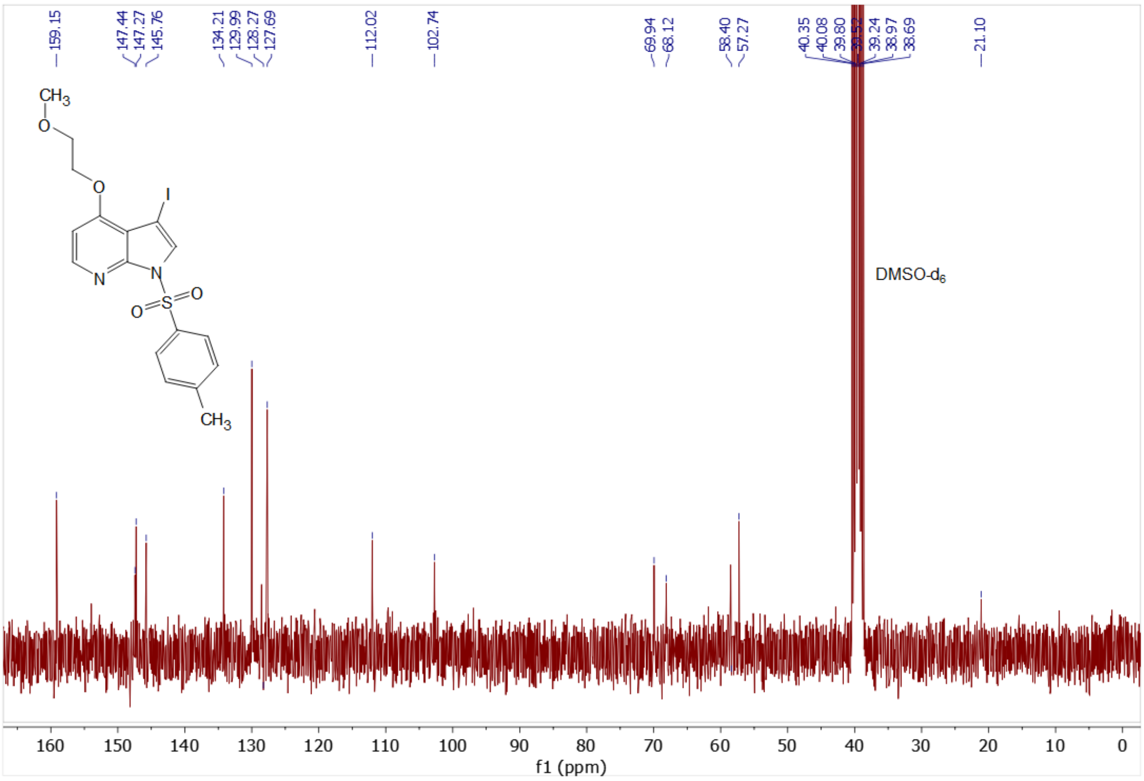

4-(Hexyloxy)-3-iodo-1-tosyl-1*H*-pyrrolo[2,3-b]pyridine (2g)

^{1}H NMR (DMSO-d_6, 600 MHz, 298 K)

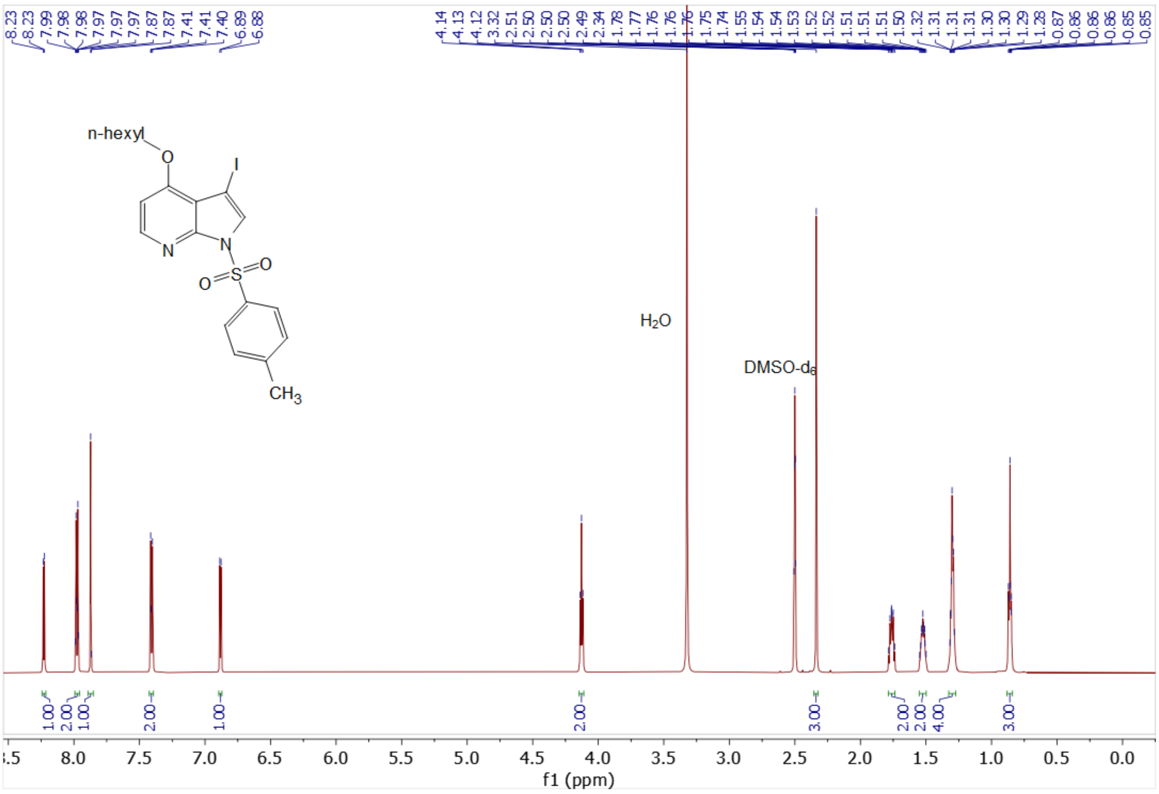

^{13}C NMR (DMSO-d_6, 150 MHz, 298 K)

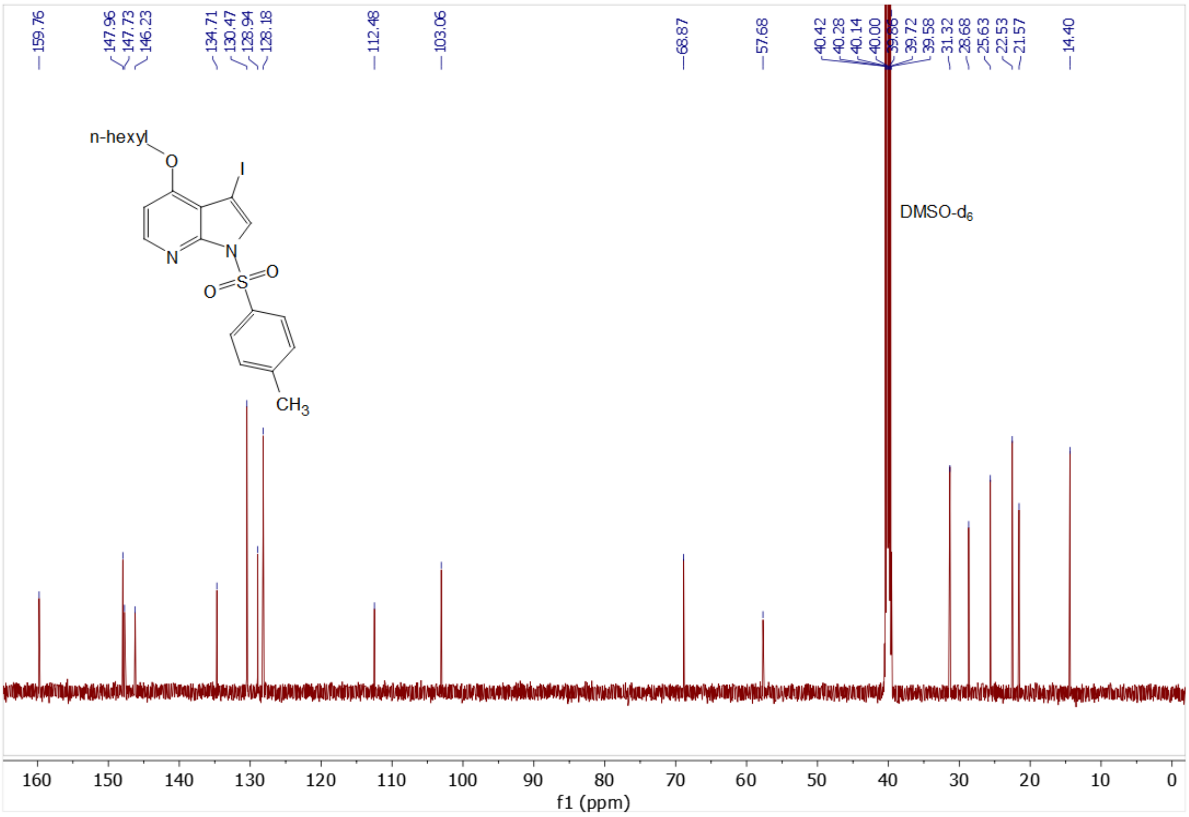

3-Iodo-4-(octyloxy)-1-tosyl-1*H*-pyrrolo[2,3-b]pyridine (3h)

^{1}H NMR (DMSO-d$_6$, 300 MHz, 298 K)

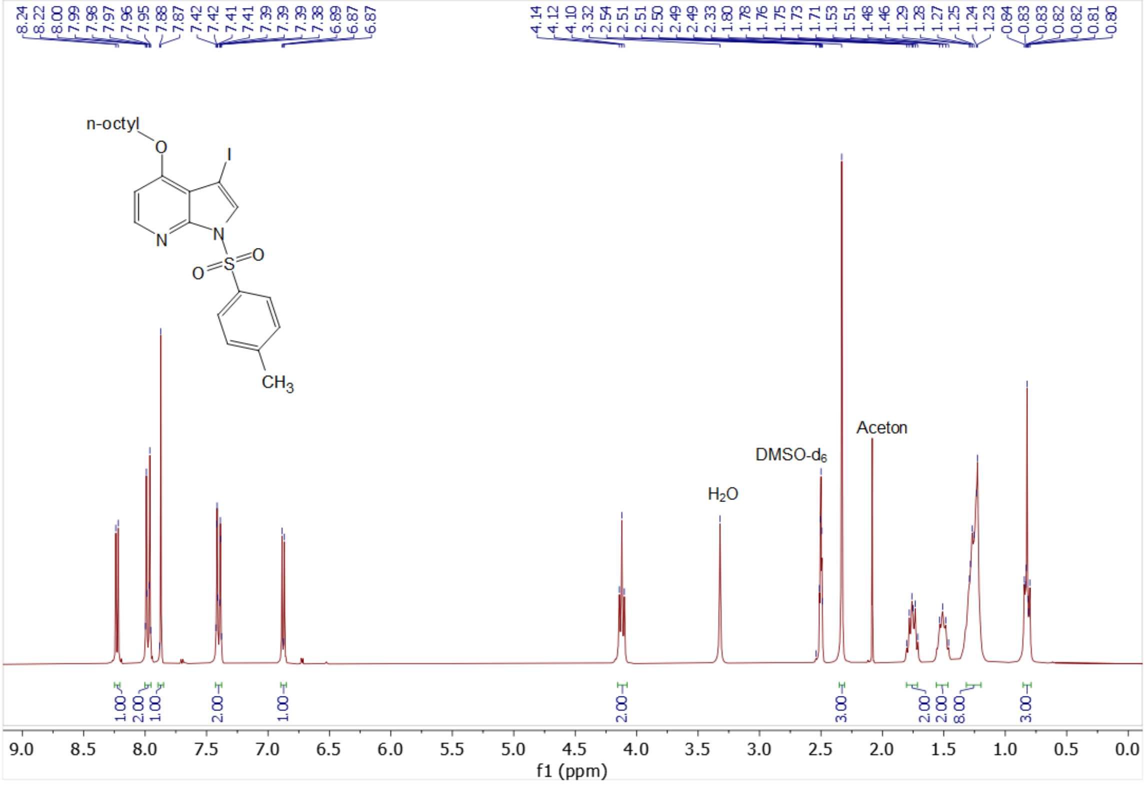

^{13}C NMR (DMSO-d$_6$, 75 MHz, 298 K)

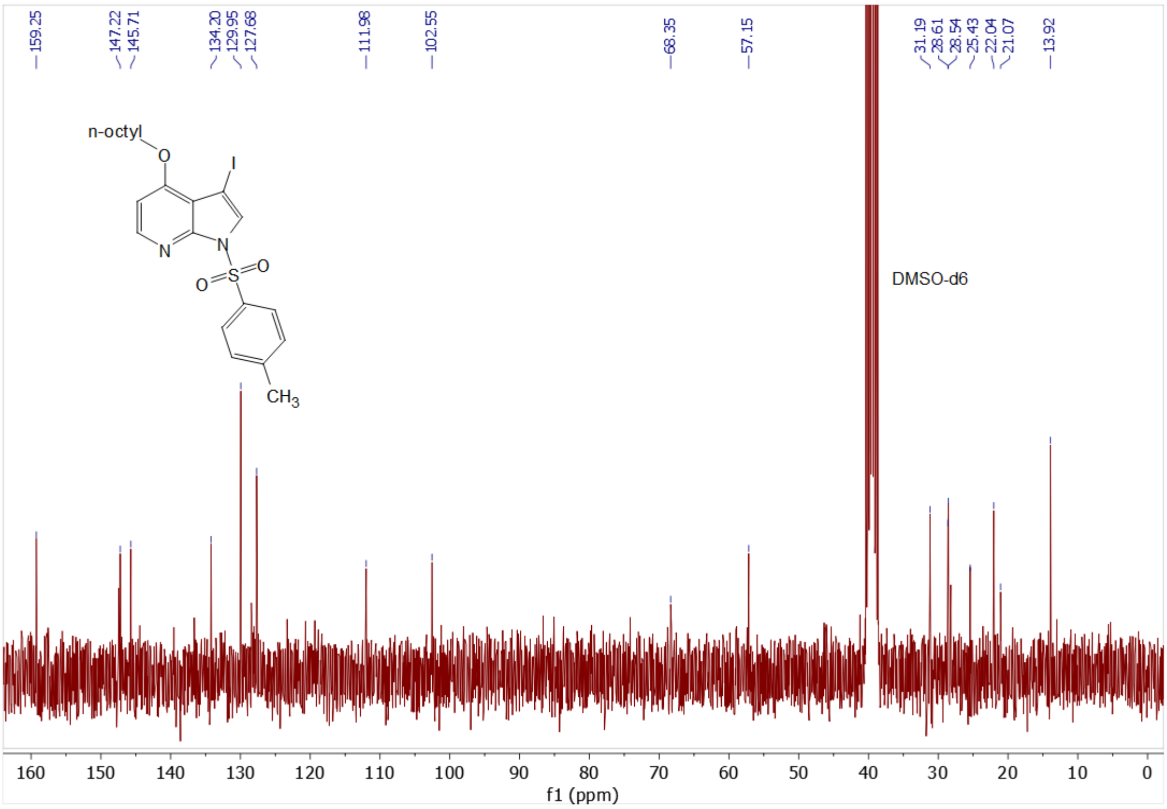

4-(Dodecyloxy)-3-iodo-1-tosyl-1*H*-pyrrolo[2,3-b]pyridine (2i)

^{1}H NMR (DMSO-d$_6$, 300 MHz, 298 K)

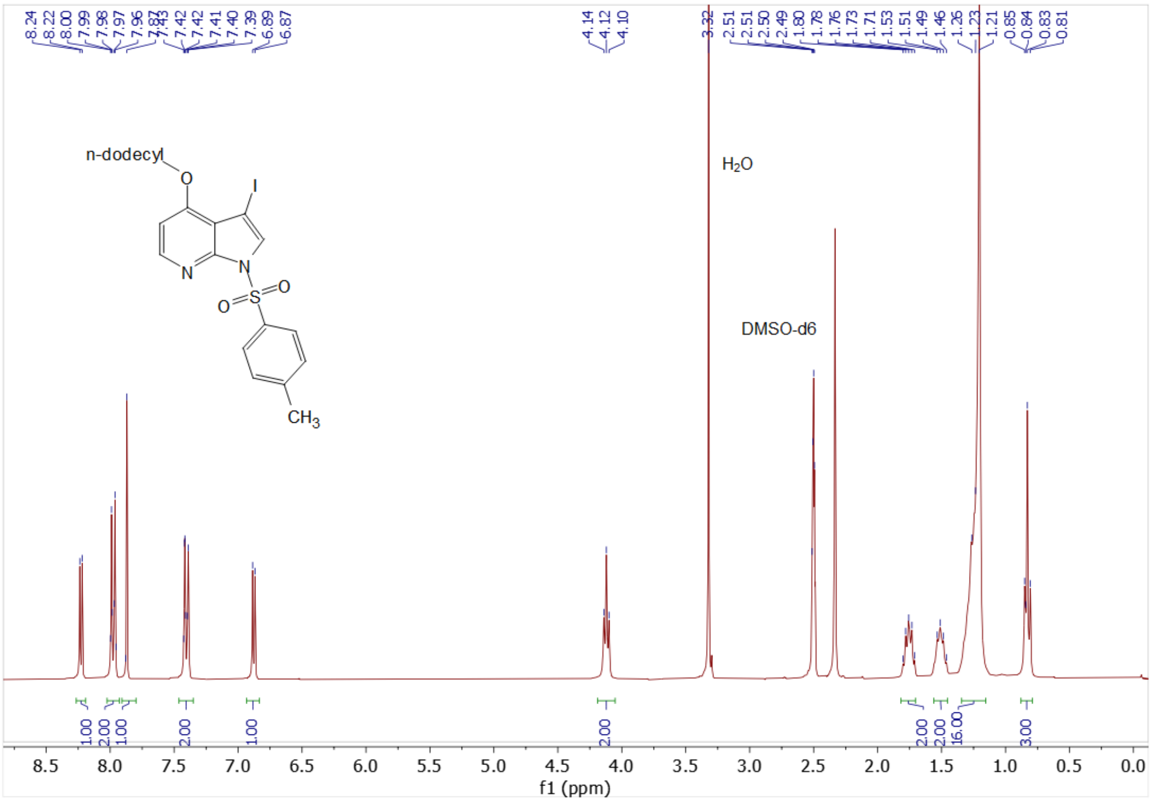

^{13}C NMR (DMSO-d$_6$, 150 MHz, 298 K)

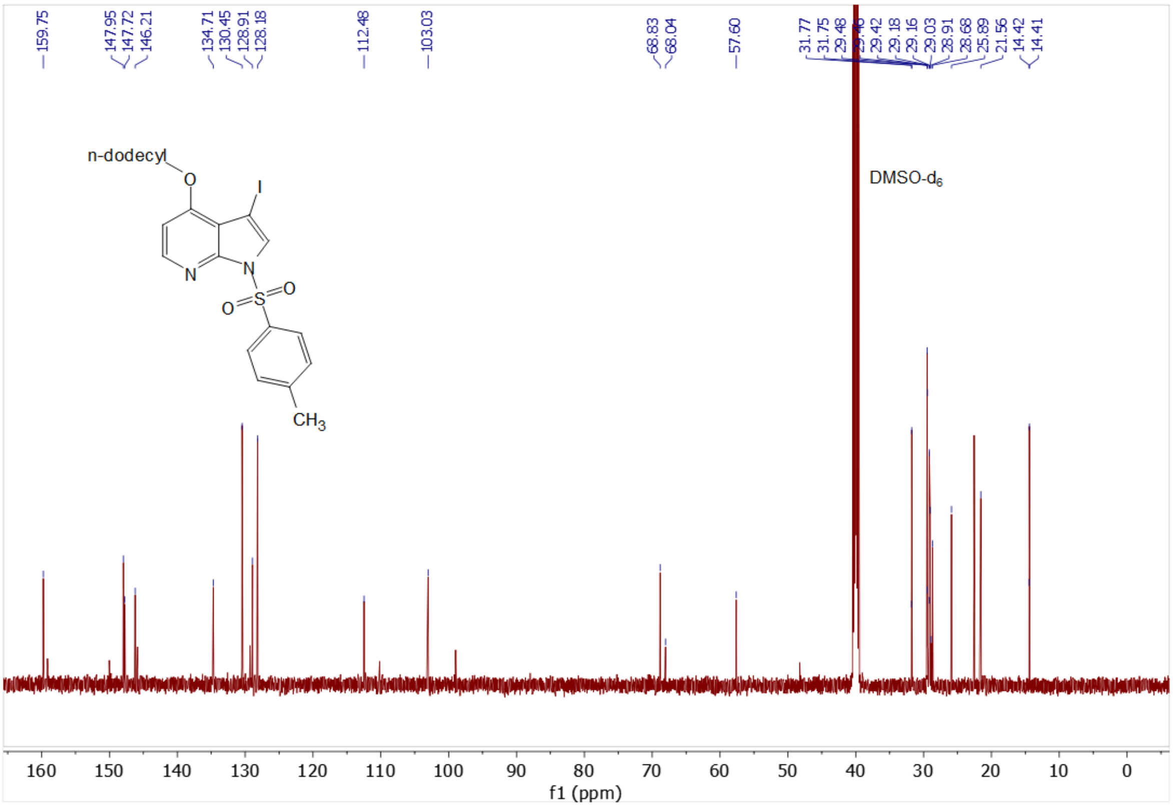

4-(4-Chloro-1*H*-pyrrolo[2,3-b]pyridin-3-yl)pyridin-2-amine (3a)

^{1}H NMR (DMSO-d$_6$, 300 MHz, 298 K)

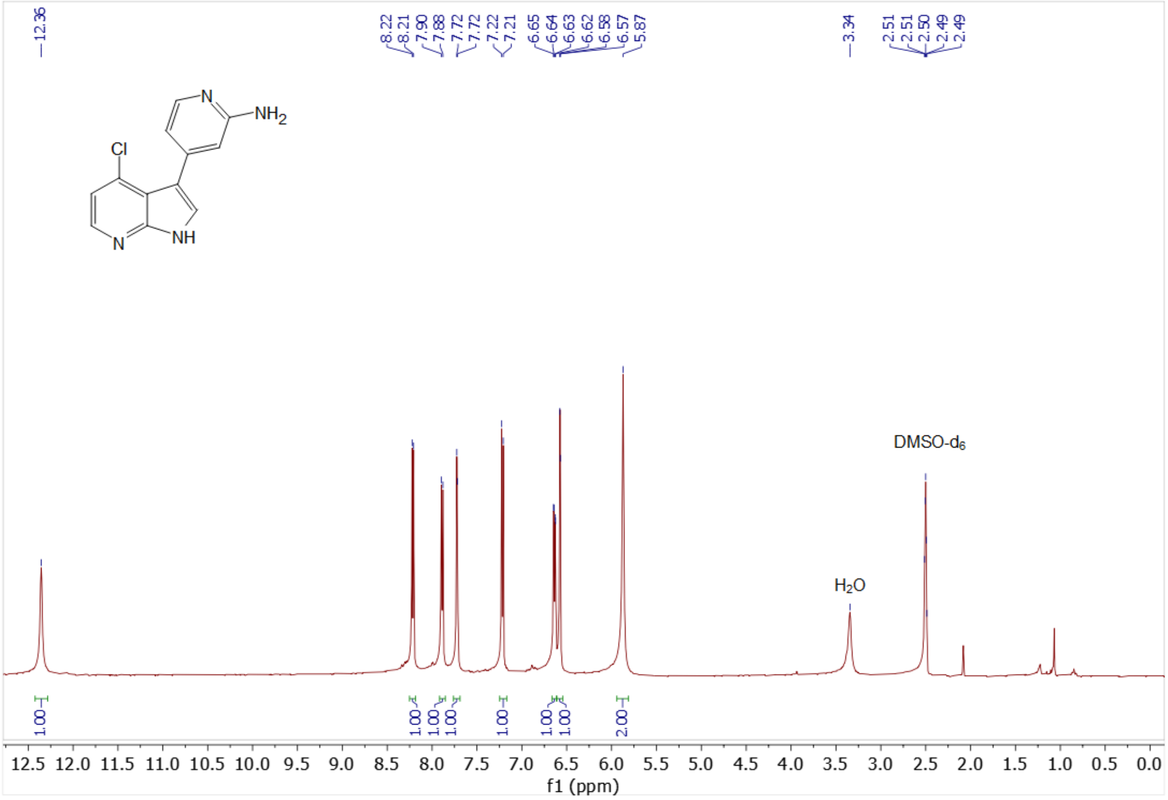

^{13}C NMR (DMSO-d$_6$, 75 MHz, 298 K)

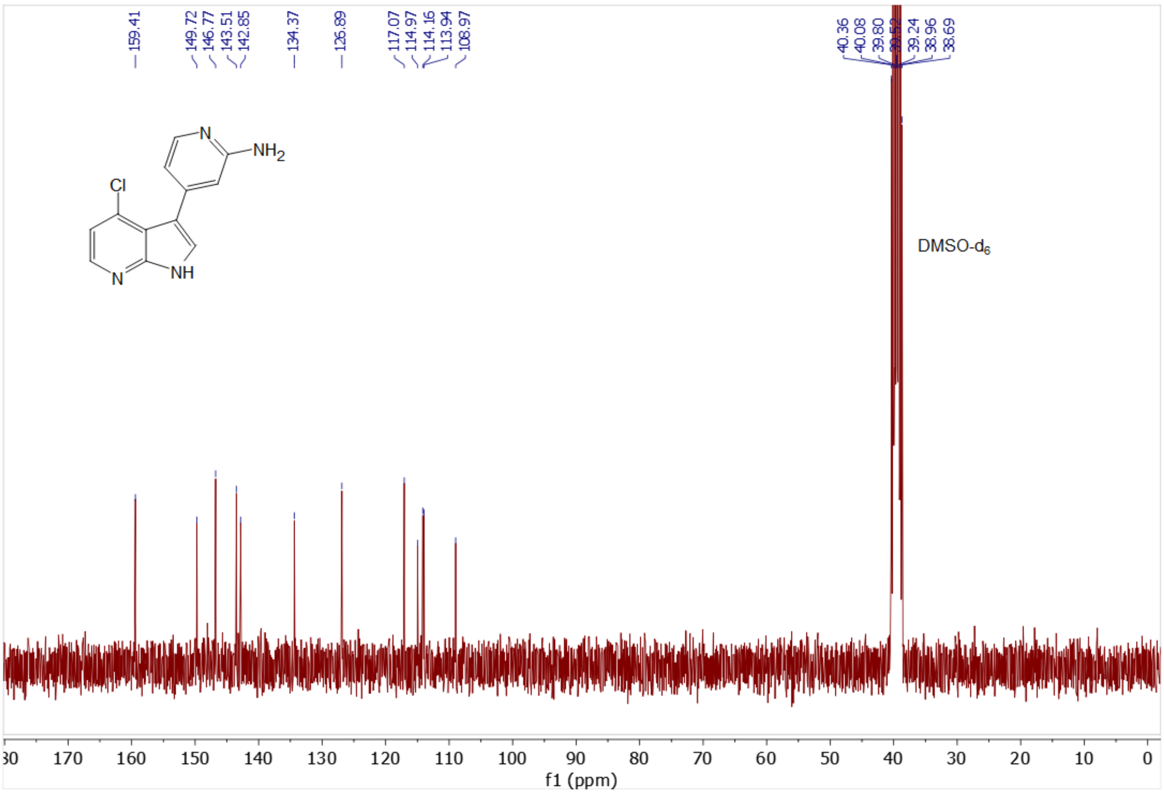

4-(4-Methoxy-1*H*-pyrrolo[2,3-b]pyridin-3-yl)pyridin-2-amine (3b)

^{1}H NMR (DMSO-d$_6$, 300 MHz, 298 K)

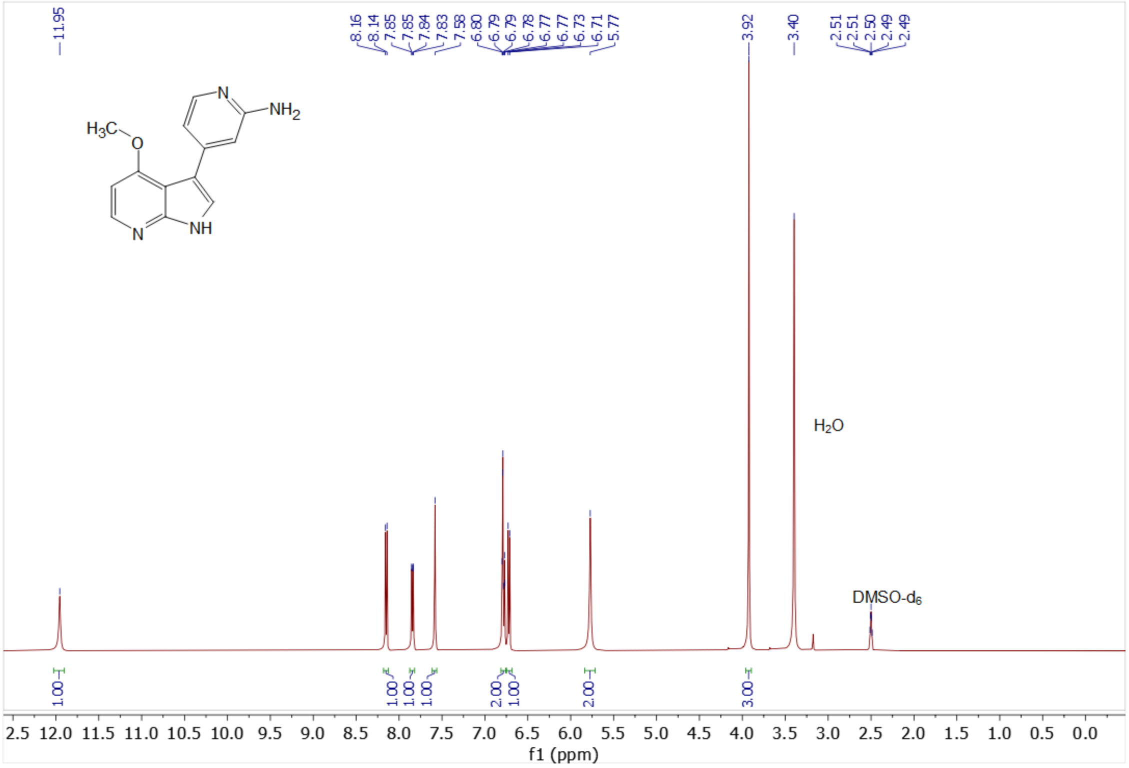

^{13}C NMR (DMSO-d$_6$, 75 MHz, 298 K)

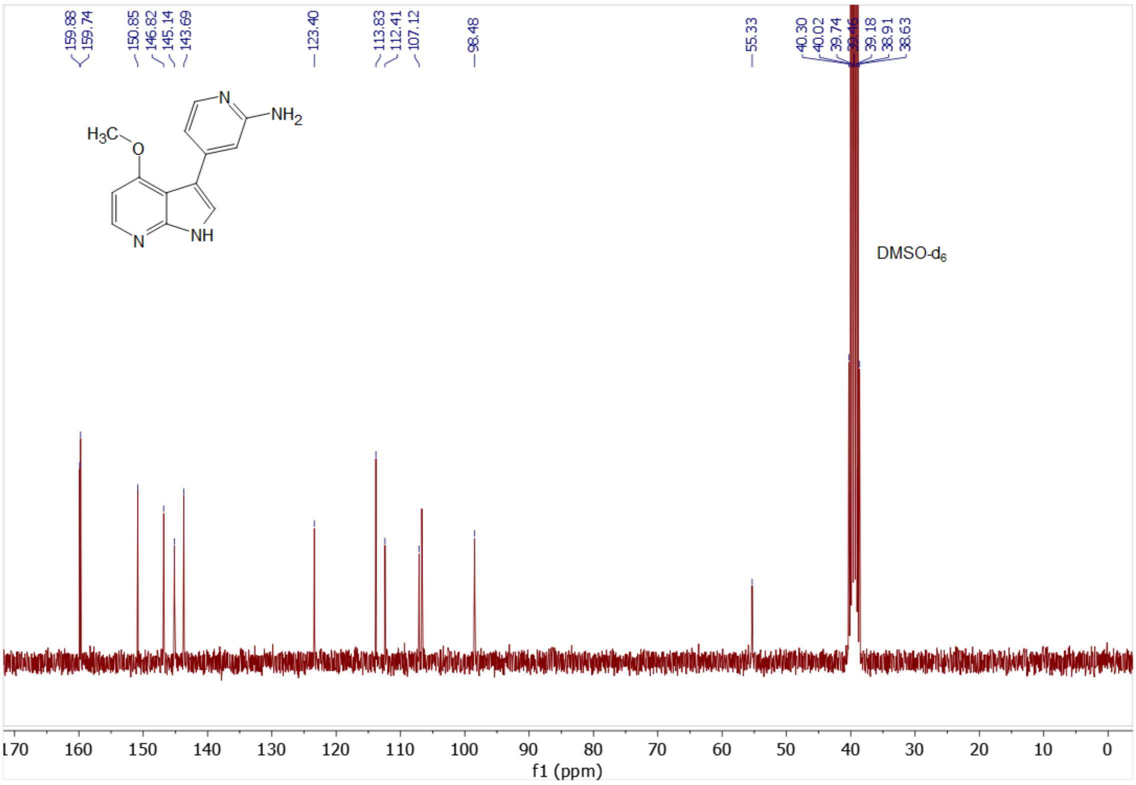

4-(4-Ethoxy-1*H*-pyrrolo[2,3-b]pyridin-3-yl)pyridin-2-amine (3c)

^{1}H NMR (DMSO-d$_6$, 300 MHz, 298 K)

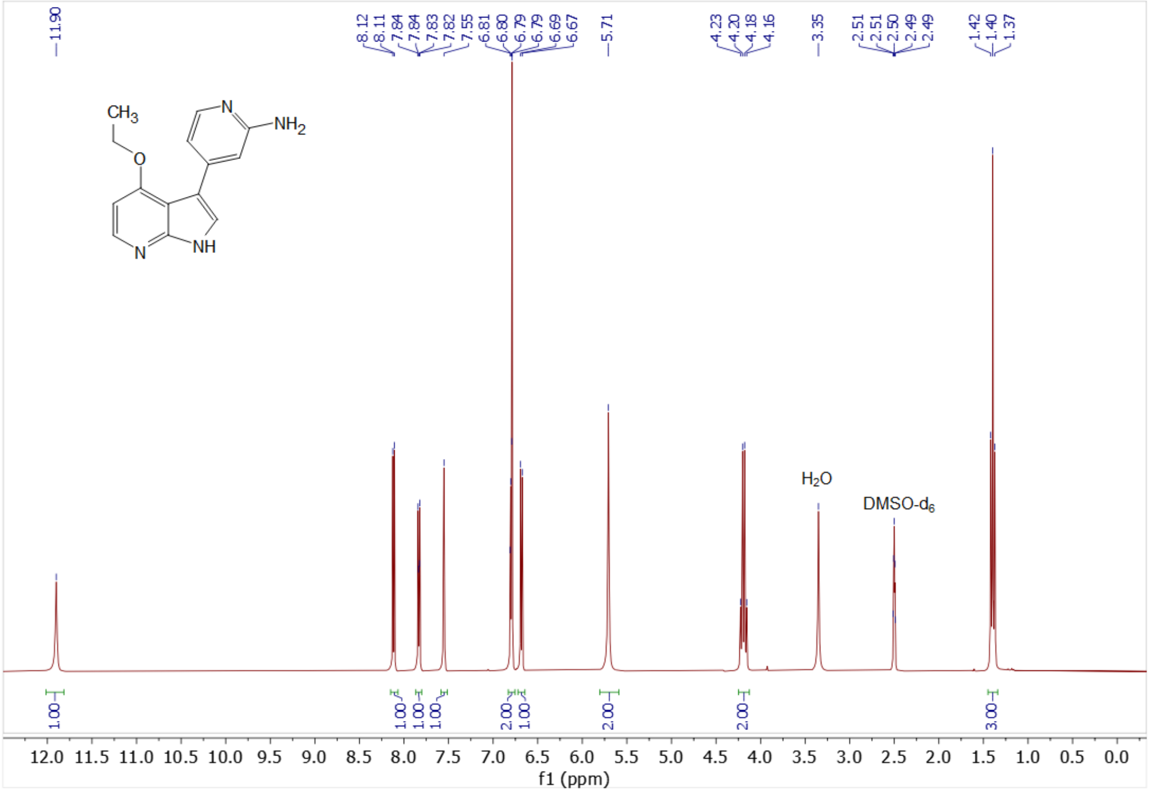

^{13}C NMR (DMSO-d$_6$, 75 MHz, 298 K)

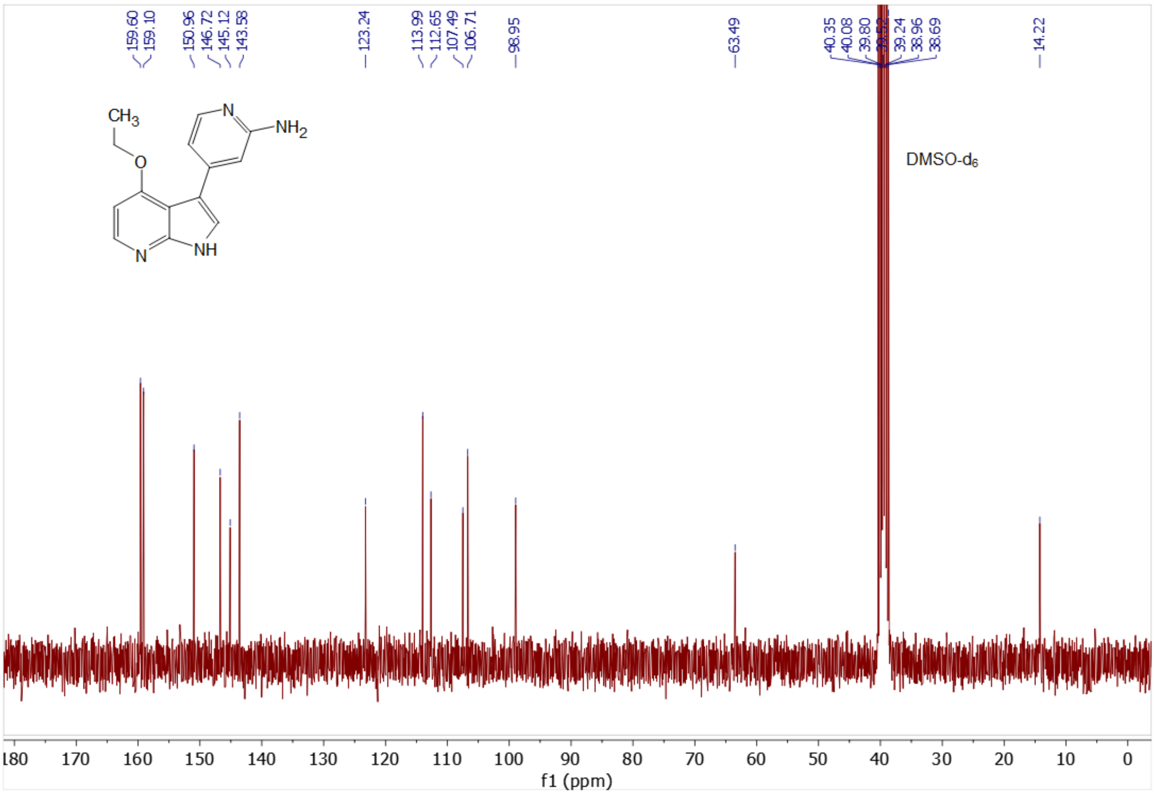

4-(4-Propoxy-1*H*-pyrrolo[2,3-b]pyridin-3-yl)pyridin-2-amine (3d)

^{1}H NMR (DMSO-d$_6$, 300 MHz, 298 K)

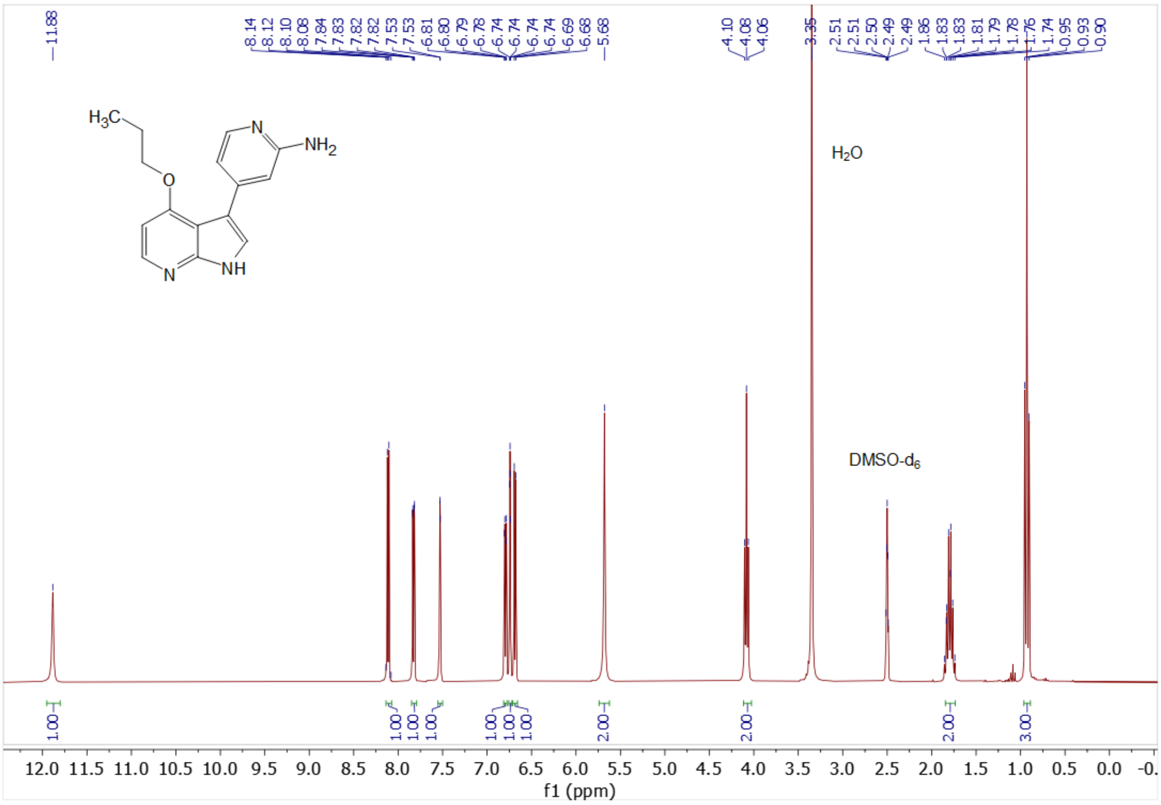

^{13}C NMR (DMSO-d$_6$, 75 MHz, 298 K)

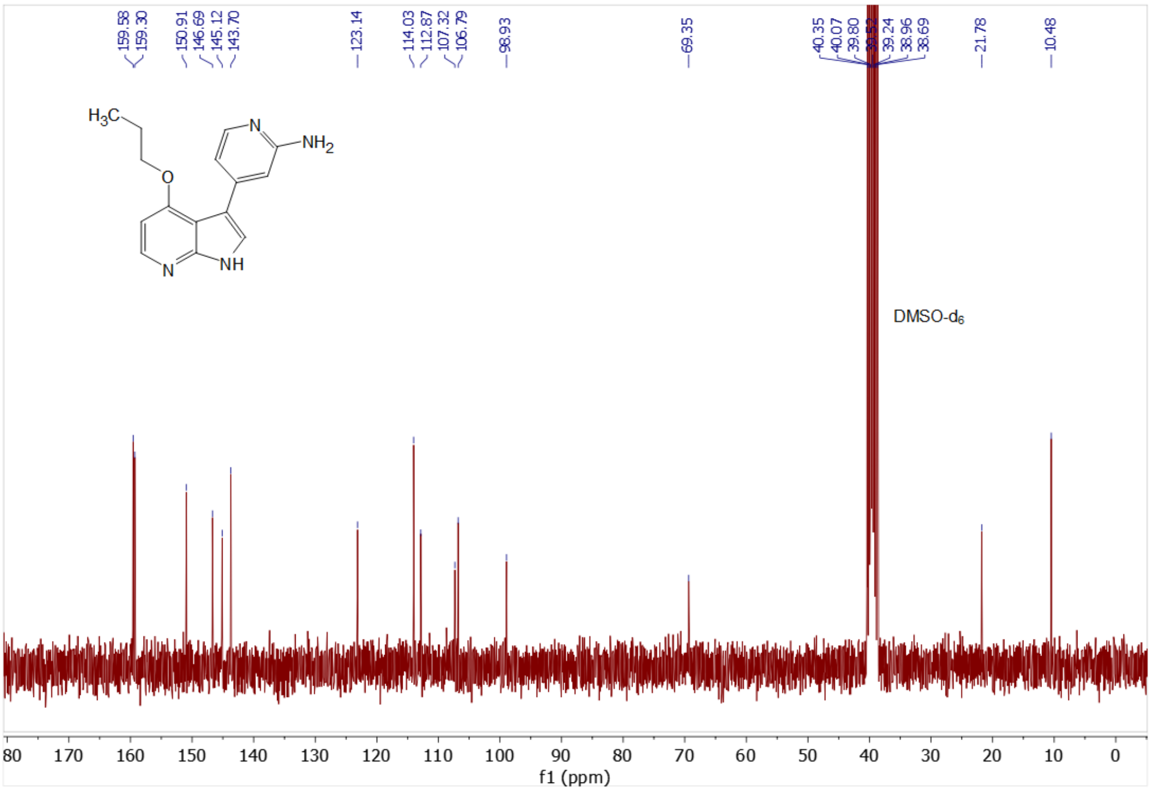

4-(4-Butoxy-1*H*-pyrrolo[2,3-b]pyridin-3-yl)pyridin-2-amine (3e)

^{1}H NMR (DMSO-d$_6$, 300 MHz, 298 K)

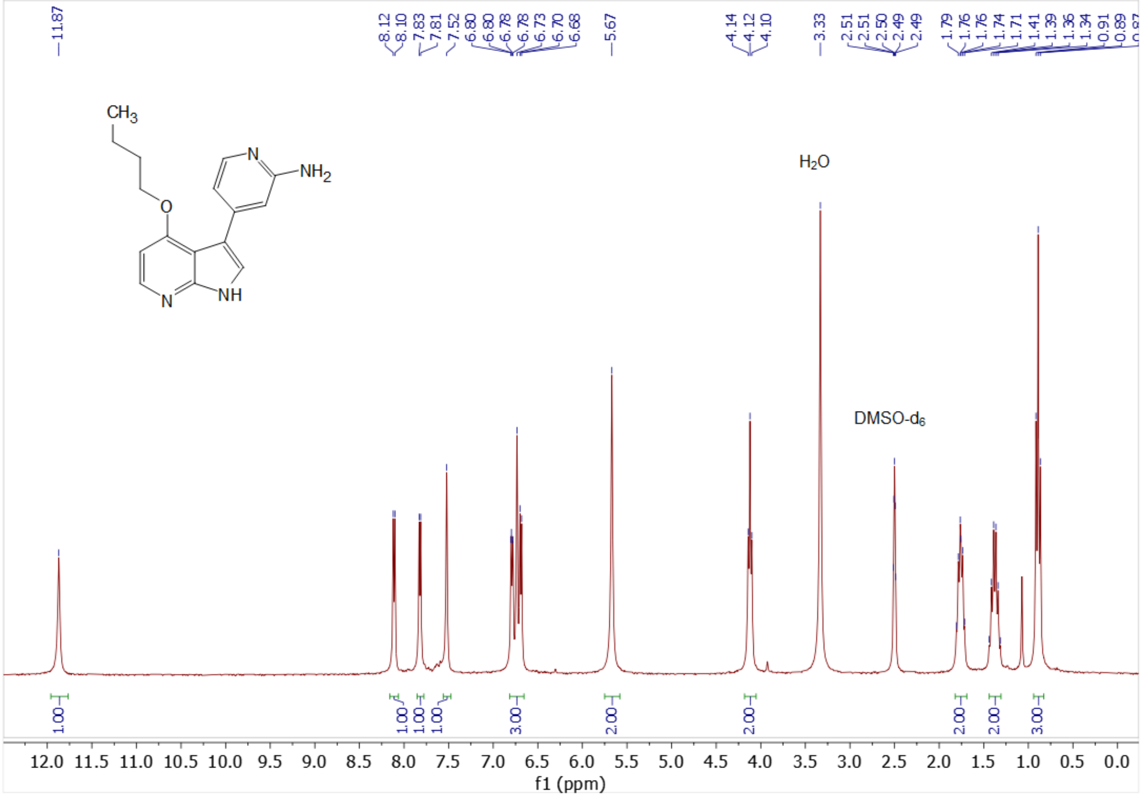

^{13}C NMR (DMSO-d$_6$, 75 MHz, 298 K)

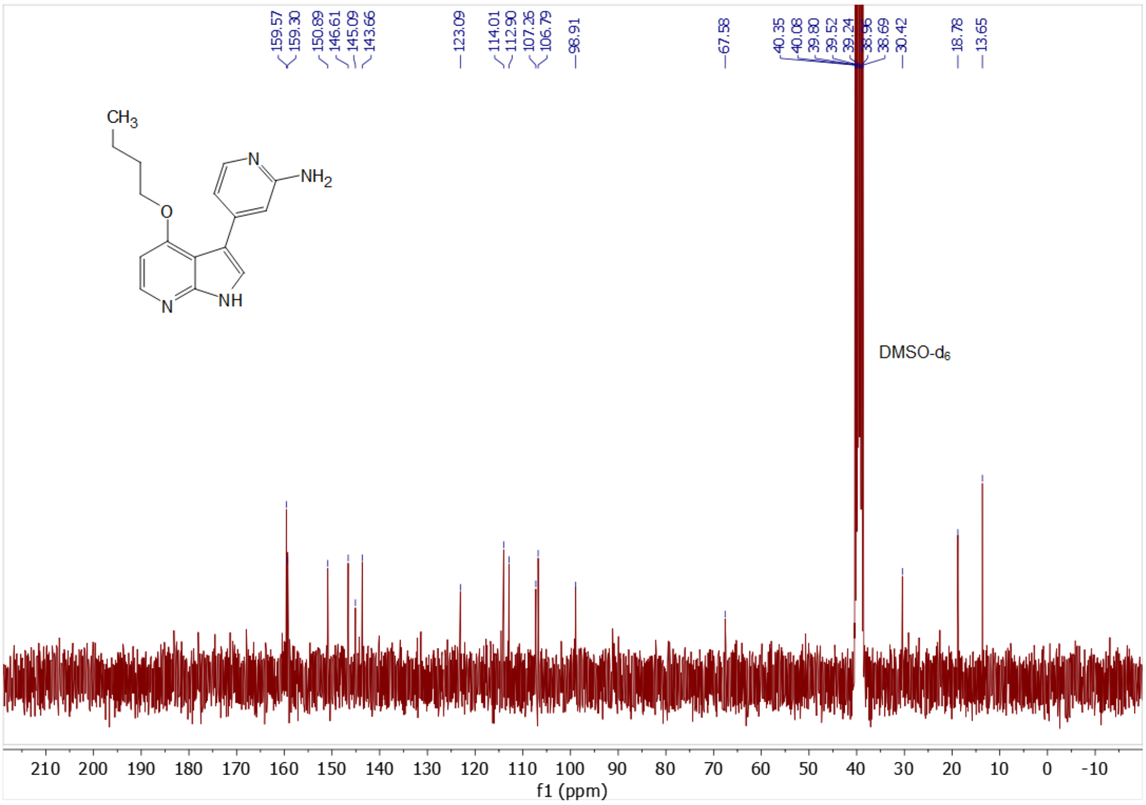

4-(4-(2-Methoxyethoxy)-1*H*-pyrrolo[2,3-b]pyridin-3-yl)pyridin-2-amine (3f)

^{1}H NMR (DMSO-d$_6$, 300 MHz, 298 K)

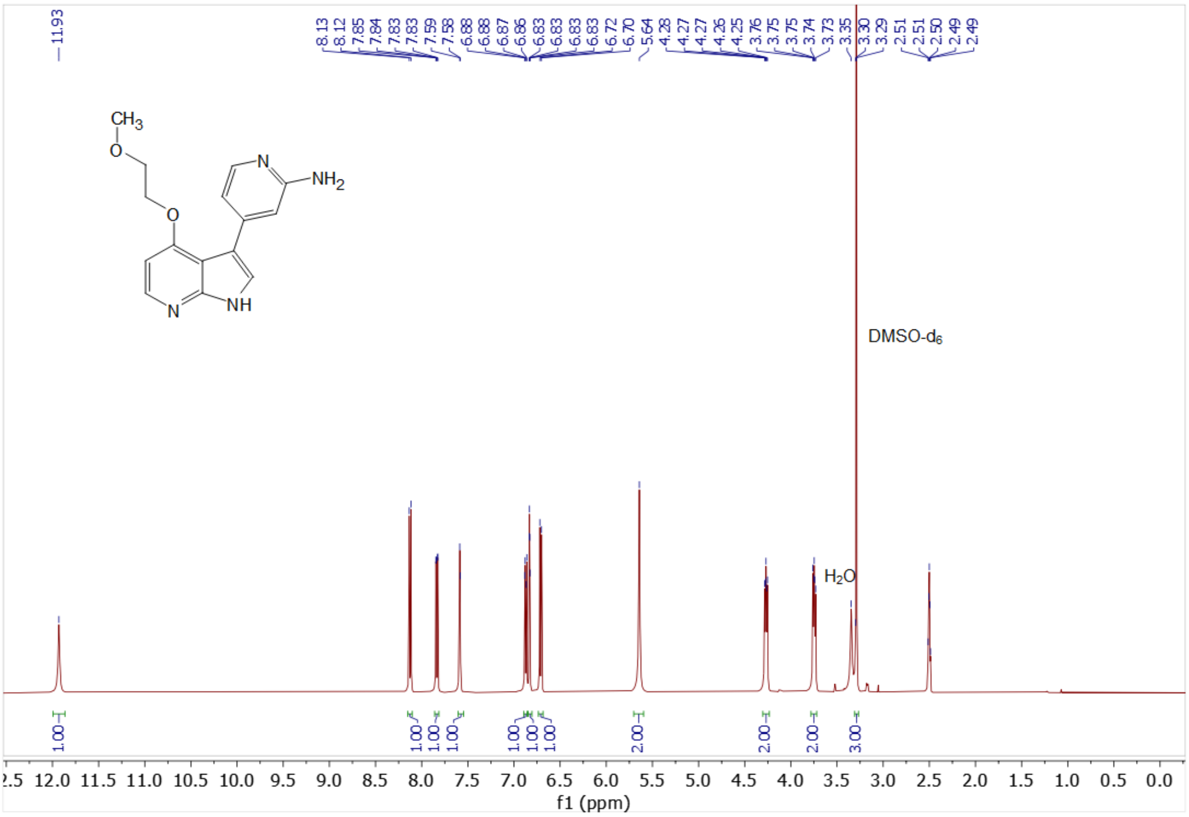

^{13}C NMR (DMSO-d$_6$, 75 MHz, 298 K)

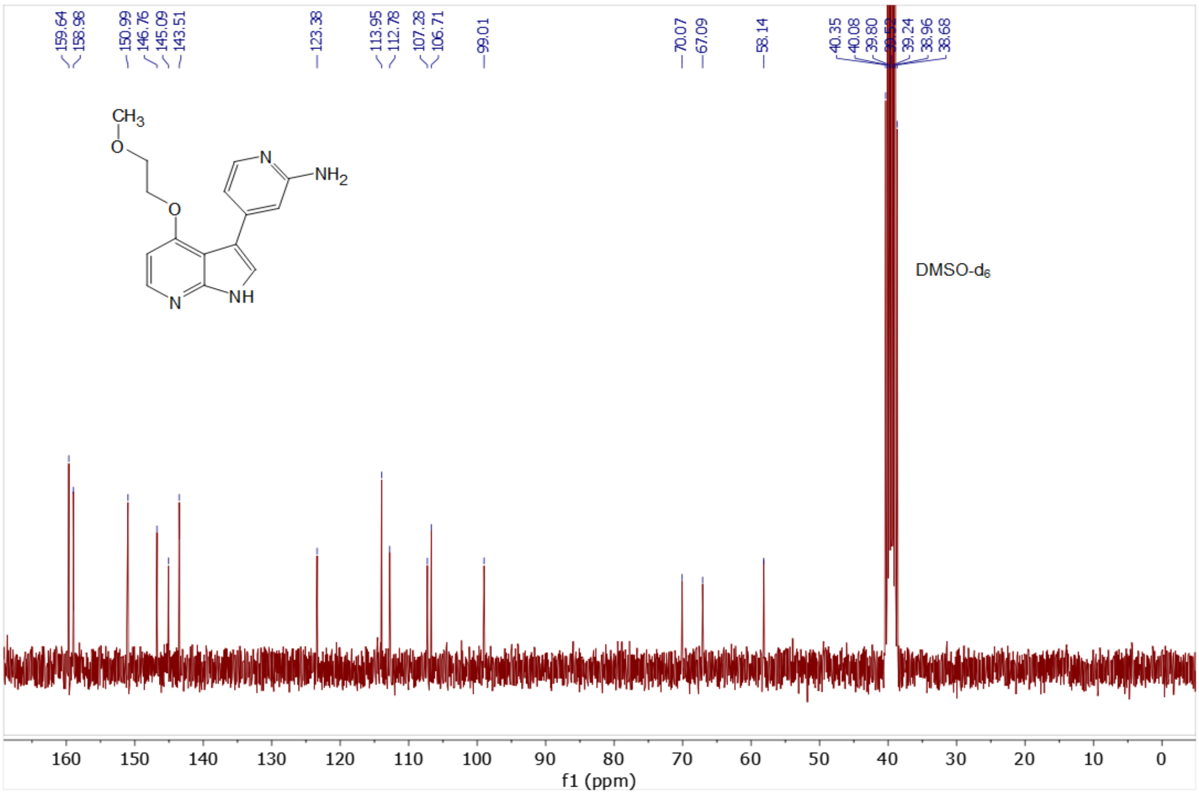

4-(4-(Hexyloxy)-1*H*-pyrrolo[2,3-b]pyridin-3-yl)pyridin-2-amine (3g)

^{1}H NMR (DMSO-d$_6$, 600 MHz, 298 K)

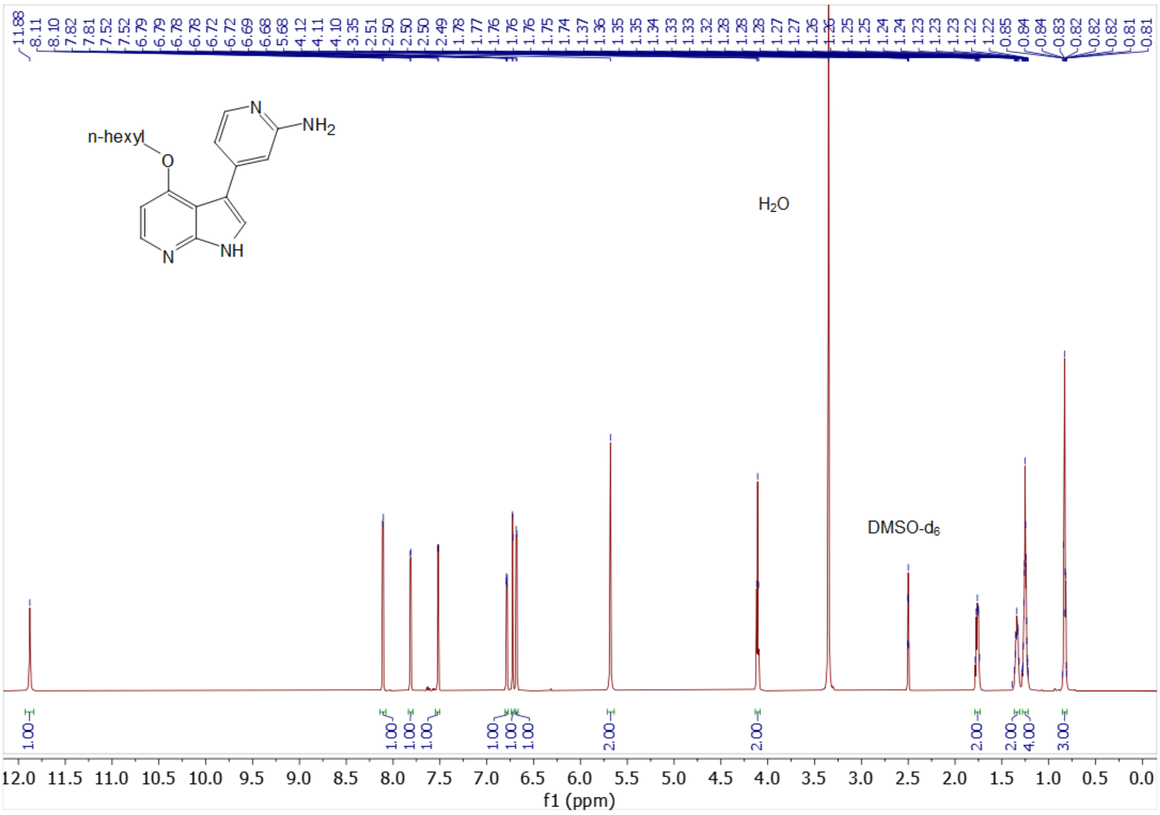

^{13}C NMR (DMSO-d$_6$, 150 MHz, 298 K)

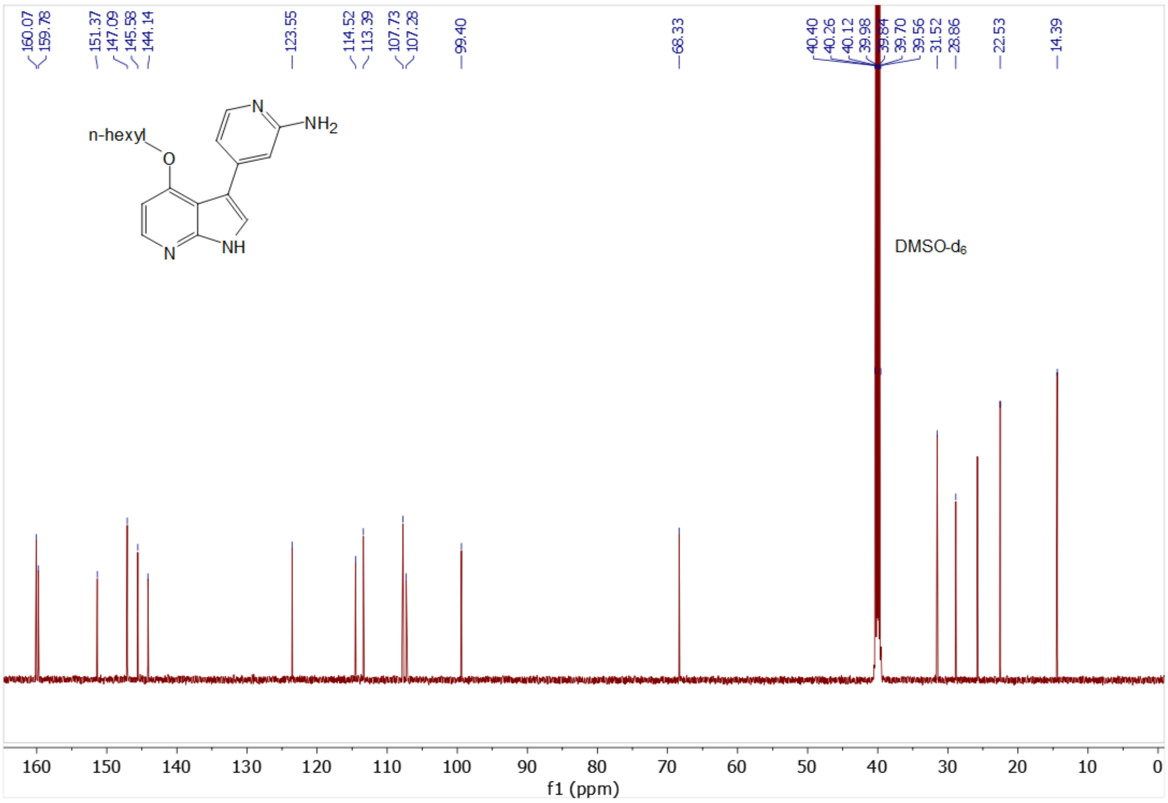

4-(4-(Octyloxy)-1*H*-pyrrolo[2,3-b]pyridin-3-yl)pyridin-2-amine (3h)

^{1}H NMR (DMSO-d_6, 600 MHz, 298 K)

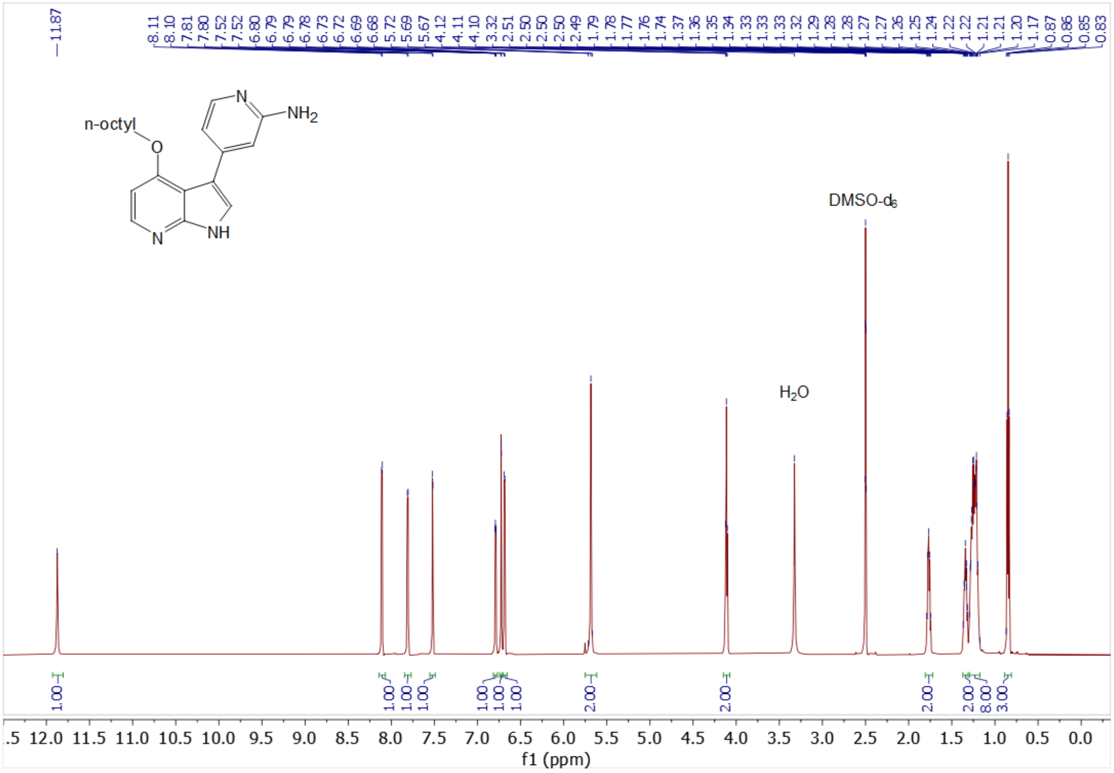

^{13}C NMR (DMSO-d_6, 150 MHz, 298 K)

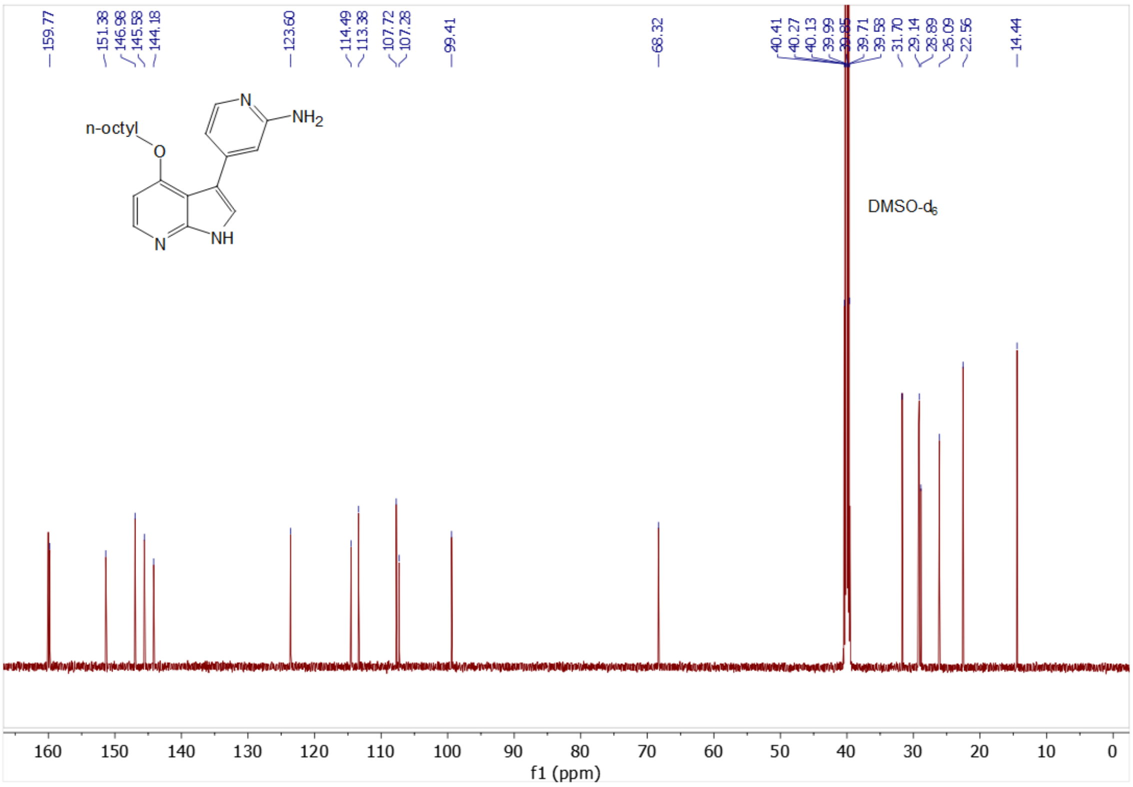

4-(4-(Dodecyloxy)-1*H*-pyrrolo[2,3-b]pyridin-3-yl)pyridin-2-amine (3i)

^{1}H NMR (DMSO-d$_6$, 600 MHz, 298 K)

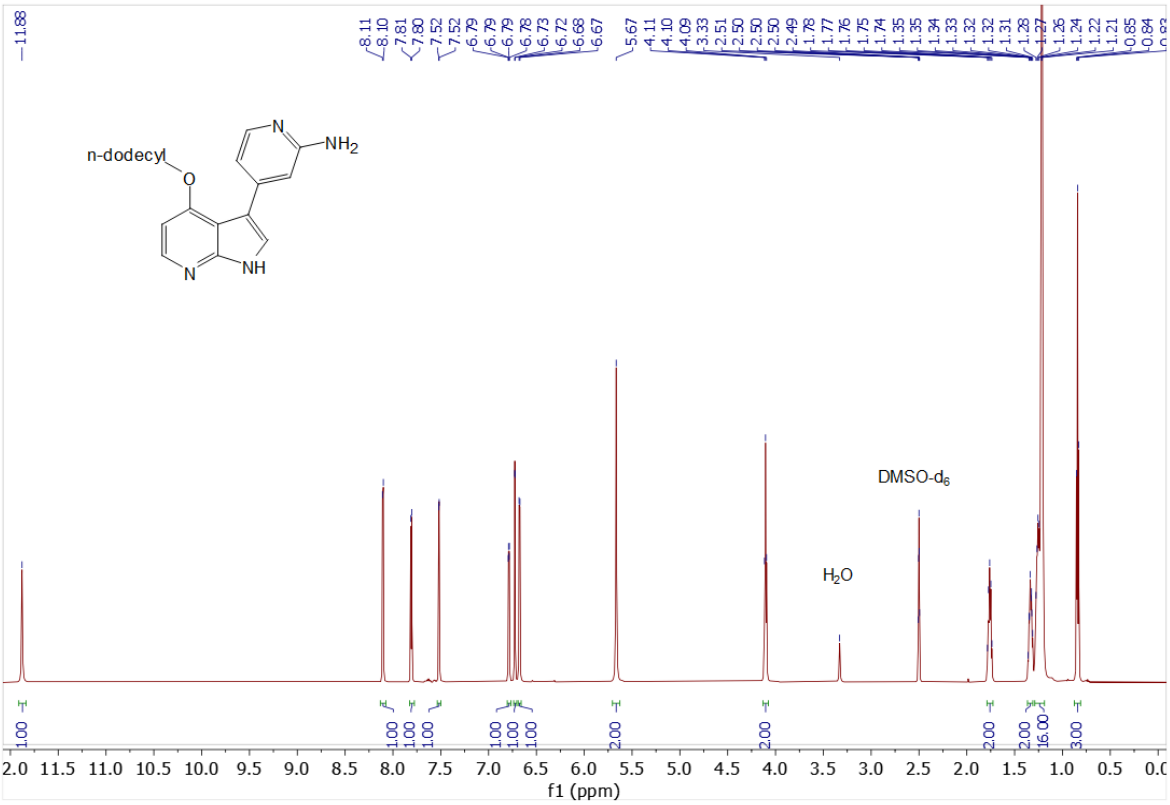

^{13}C NMR (DMSO-d$_6$, 150 MHz, 298 K)

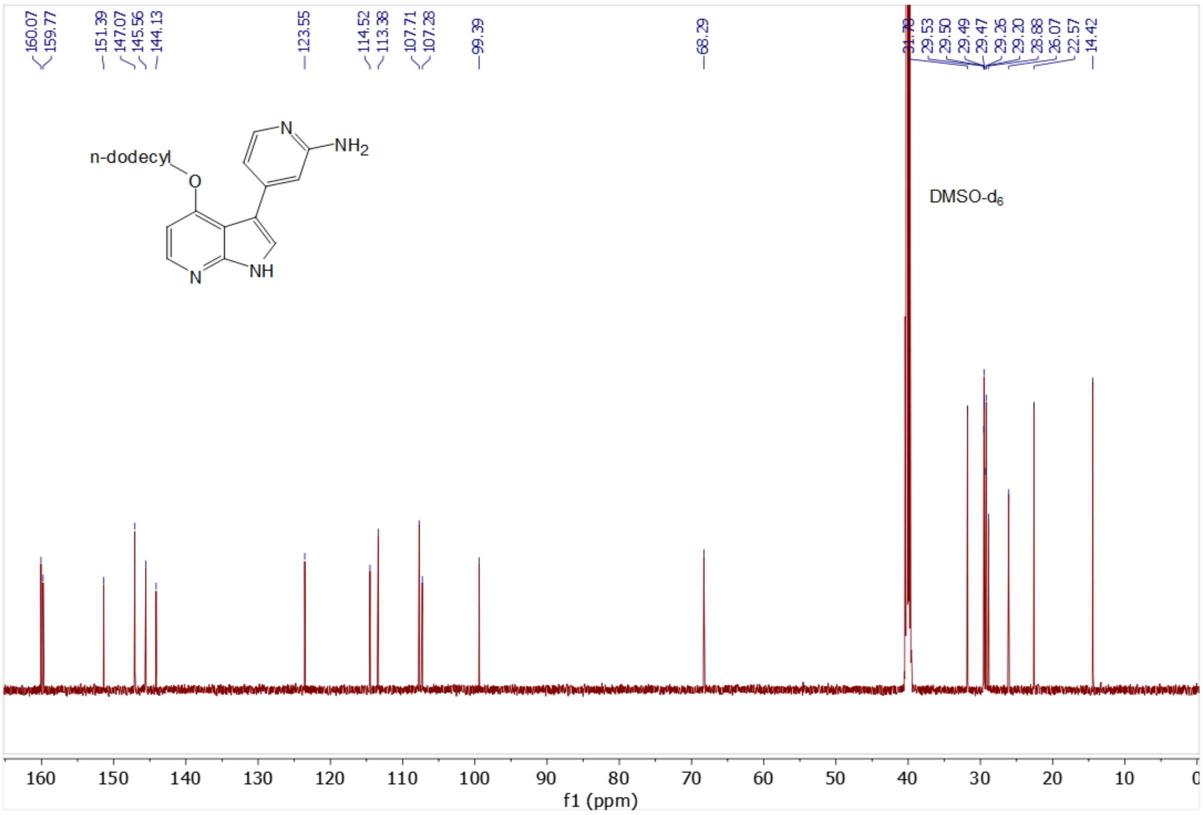

6. References

1. Echalier, A.; Bettayeb, K.; Ferandin, Y.; Lozach, O.; Clément, M.; Valette, A.; Liger, F.; Marquet, B.; Morris, J. C.; Endicott, J. A.; Joseph, B.; Meijer, L., *Meriolins* (3-(Pyrimidin-4-yl)-7-azaindoles): Synthesis, Kinase Inhibitory Activity, Cellular Effects, and Structure of a CDK2/Cyclin A/*Meriolin* Complex. *J. Med. Chem.* **2008**, *51*, 737-751. DOI: 10.1021/jm700940h.
2. Drießen, D.; Stuhldreier, F.; Frank, A.; Stark, H.; Wesselborg, S.; Stork, B.; Müller, T. J. J., Novel *meriolin* derivatives as rapid apoptosis inducers. *Bioorg. Med. Chem.* **2019**, *27*, 3463-3468. DOI: 10.1016/j.bmc.2019.06.029.
